# Morbidity and mortality from road injuries: results from the Global Burden of Disease Study 2017

**DOI:** 10.1136/injuryprev-2019-043302

**Published:** 2020-01-08

**Authors:** Spencer L James, Lydia R Lucchesi, Catherine Bisignano, Chris D Castle, Zachary V Dingels, Jack T Fox, Erin B Hamilton, Zichen Liu, Darrah McCracken, Molly R Nixon, Dillon O Sylte, Nicholas L S Roberts, Oladimeji M Adebayo, Teamur Aghamolaei, Suliman A Alghnam, Syed Mohamed Aljunid, Amir Almasi-Hashiani, Alaa Badawi, Masoud Behzadifar, Meysam Behzadifar, Eyasu Tamru Bekru, Derrick A Bennett, Jens Robert Chapman, Kebede Deribe, Bereket Duko Adema, Yousef Fatahi, Belayneh K Gelaw, Eskezyiaw Agedew Getahun, Delia Hendrie, Andualem Henok, Hagos de Hidru, Mehdi Hosseinzadeh, Guoqing Hu, Mohammad Ali Jahani, Mihajlo Jakovljevic, Farzad Jalilian, Nitin Joseph, Manoochehr Karami, Abraham Getachew Kelbore, Md Nuruzzaman Khan, Yun Jin Kim, Parvaiz A Koul, Carlo La Vecchia, Shai Linn, Reza Majdzadeh, Man Mohan Mehndiratta, Peter T N Memiah, Melkamu Merid Mengesha, Hayimro Edemealem Merie, Ted R Miller, Mehdi Mirzaei-Alavijeh, Aso Mohammad Darwesh, Naser Mohammad Gholi Mezerji, Roghayeh Mohammadibakhsh, Yoshan Moodley, Maziar Moradi-Lakeh, Kamarul Imran Musa, Bruno Ramos Nascimento, Rajan Nikbakhsh, Peter S Nyasulu, Ahmed Omar Bali, Obinna E Onwujekwe, Sanghamitra Pati, Reza Pourmirza Kalhori, Farkhonde Salehi, Saeed Shahabi, Seifadin Ahmed Shallo, Morteza Shamsizadeh, Zeinab Sharafi, Sharvari Rahul Shukla, Mohammad Reza Sobhiyeh, Joan B Soriano, Bryan L Sykes, Rafael Tabarés-Seisdedos, Degena Bahray Bahrey Tadesse, Yonatal Mesfin Tefera, Arash Tehrani-Banihashemi, Boikhutso Tlou, Roman Topor-Madry, Taweewat Wiangkham, Mehdi Yaseri, Sanni Yaya, Muluken Azage Yenesew, Mustafa Z Younis, Arash Ziapour, Sanjay Zodpey, David M Pigott, Robert C Reiner, Simon I Hay, Alan D Lopez, Ali H Mokdad

**Affiliations:** 1 Institute for Health Metrics and Evaluation, University of Washington, Seattle, Washington, USA; 2 College of Medicine, University College Hospital, Ibadan, Nigeria; 3 Department of Health Education and Health Promotion, Hormozgan University of Medical Sciences, Bandar Abbas, Iran; 4 Department of Population Health Research, King Abdullah International Medical Research Center, Riyadh, Saudi Arabia; 5 Department of Health Policy and Management, Kuwait University, Safat, Kuwait; 6 International Centre for Casemix and Clinical Coding, National University of Malaysia, Bandar Tun Razak, Malaysia; 7 Department of Epidemiology, Arak University of Medical Sciences, Arak, Iran; 8 Public Health Risk Sciences Division, Public Health Agency of Canada, Toronto, Ontario, Canada; 9 Department of Nutritional Sciences, University of Toronto, Toronto, Ontario, Canada; 10 Social Determinants of Health Research Center, Lorestan University of Medical Sciences, Khorramabad, Iran; 11 Hepatitis Research Center, Lorestan University of Medical Sciences, Khorramabad, Iran; 12 College of Health Science and Medicine, Wolaita Sodo University, Sodo, Ethiopia; 13 Nuffield Department of Population Health, University of Oxford, Oxford, UK; 14 Swedish Neuroscience Institute, Swedish Brain and Spine Specialists, Seattle, Washington, USA; 15 Department of Global Health and Infection, Brighton and Sussex Medical School, Brighton, UK; 16 School of Public Health, Addis Ababa University, Addis Ababa, Ethiopia; 17 Department of Public Health, Hawassa University, Hawassa, Ethiopia; 18 Department of Pharmaceutical Nanotechnology, Tehran University of Medical Sciences, Tehran, Iran; 19 School of Pharmacy, Debre Tabor University, Ambo, Ethiopia; 20 Department of Public Health, Arba Minch University, Arba Minch City, Ethiopia; 21 School of Public Health, Curtin University, Perth, Western Australia, Australia; 22 Department of Public Health, Mizan-Tepi University, Teppi, Ethiopia; 23 Department of Biostatistics and Epidemiology, Adigrat University, Adigrat, Ethiopia; 24 Health Management and Economics Research Center, Iran University of Medical Sciences, Tehran, Iran; 25 Computer Science Department, University of Human Development, Sulaimaniyah, Iraq; 26 Department of Epidemiology and Health Statistics, Central South University, Changsha, China; 27 Faculty of Medicine, Babol University of Medical Sciences, Babol, Iran; 28 N.A.Semashko Department of Public Health and Healthcare, I.M. Sechenov First Moscow State Medical University (Sechenov University), Moscow, Russia; 29 Social Development & Health Promotion Research Center, Kermanshah University of Medical Sciences, Kermanshah, Iran; 30 Department of Community Medicine, Kasturba Medical College, Manipal University, Mangalore, India; 31 Department of Epidemiology, Hamadan University of Medical Sciences, Hamadan, Iran; 32 Department of Dermatology, Wolaita Sodo University, Wolaita Sodo, Ethiopia; 33 Department of Population Sciences, Jatiya Kabi Kazi Nazrul Islam University, Mymensingh, Bangladesh; 34 Department of Public Health, University of Newcastle, Newcastle, New South Wales, Australia; 35 School of Medicine, Xiamen University Malaysia, Sepang, Malaysia; 36 Department of Internal and Pulmonary Medicine, Sheri Kashmir Institute of Medical Sciences, Srinagar, India; 37 Department of Clinical Medicine and Community Health, University of Milan, Milano, Italy; 38 School of Public Health, University of Haifa, Haifa, Israel; 39 Community-Based Participatory-Research (CBPR) Center, Tehran University of Medical Sciences, Tehran, Iran; 40 Knowledge Utilization Research Center (KURC), Tehran University of Medical Sciences, Tehran, Iran; 41 Neurology Department, Janakpuri Super Specialty Hospital Society, New Delhi, India; 42 Department of Neurology, New Delhi, India; 43 Department of Public Health, University of West Florida, Pensacola, Florida, USA; 44 Department of Epidemiology and Biostatistics, Haramaya University, Harar, Ethiopia; 45 Department of Statistics, Debre Markos University, Debre Markos, Ethiopia; 46 Pacific Institute for Research and Evaluation, Calverton, Maryland, United States; 47 Information Technology Department, University of Human Development, Sulaimaniyah, Iraq; 48 Department of Biostatistics, Hamadan University of Medical Sciences, Hamadan, Iran; 49 Hamadan University of Medical Sciences, Hamadan, Iran; 50 Department of Public Health Medicine, University of KwaZulu-Natal, Durban, South Africa; 51 Preventive Medicine and Public Health Research Center, Iran University of Medical Sciences, Tehran, Iran; 52 School of Medical Sciences, Science University of Malaysia, Kubang Kerian, Malaysia; 53 Hospital of the Federal University of Minas Gerais, Federal University of Minas Gerais, Belo Horizonte, Brazil; 54 Obesity Research Center, Research Institute for Endocrine Science, Shahid Beheshti University of Medical Sciences, Tehran, Iran; 55 Faculty of Medicine & Health Sciences, Stellenbosch University, Cape Town, South Africa; 56 Department of Diplomacy and Public Relations, University of Human Development, Sulaimaniyah, Iraq; 57 Department of Pharmacology and Therapeutics, University of Nigeria Nsukka, Enugu, Nigeria; 58 Regional Medical Research Centre, Indian Council of Medical Research, Bhubaneswar, India; 59 Paramedic Department, Kermanshah University of Medical Sciences, Kermanshah, Iran; 60 Taleghani Hospital, Kermanshah University of Medical Sciences, Kermanshah, Iran; 61 Health Policy Research Center, Institute of Health, Shiraz University of Medical Sciences, Shiraz, Iran; 62 Department of Public Health, Ambo University, Ambo, Ethiopia; 63 Chronic Diseases (Home Care) Research Center, Hamadan University of Medical Sciences, Hamadan, Iran; 64 Razi Herbal Medicines Research Center, Lorestan University of Medical Sciences, Khorramabad, Iran; 65 Symbiosis Institute of Health Sciences, Symbiosis International University, Pune, India; 66 Departments of Vascular & Endovascular Surgery, General Surgery, Kermanshah University of Medical Sciences, Kermanshah, Iran; 67 Peripheral Vascular Intervention Department, Kermanshah University of Medical Sciences, Kermanshah, Iran; 68 Hospital Universitario de la Princesa, Autonomous University of Madrid, Madrid, Spain; 69 Respiratory Diseases Networking Biomedical Research Centre (CIBERES), Institute of Health Carlos III, Madrid, Spain; 70 Department of Criminology, Law and Society, University of California Irvine, Irvine, California, USA; 71 Department of Medicine, University of Valencia, Valencia, Spain; 72 Carlos III Health Institute, Biomedical Research Networking Center for Mental Health Network (CiberSAM), Madrid, Spain; 73 Nursing Department, Institute of Tropical Medicine, Aksum, Ethiopia; 74 Axum College of Health Science, Mekelle, Ethiopia; 75 School of Public Health, University of Adelaide, Adelaide, South Australia, Australia; 76 Department of Environmental Health, Wollo University, Dessie, Ethiopia; 77 Department of Community Medicine, Iran University of Medical Sciences, Tehran, Iran; 78 Department of Public Health, University of KwaZulu-Natal, Durban, South Africa; 79 Faculty of Health Sciences, Jagiellonian University Medical College, Krakow, Poland; 80 The Agency for Health Technology Assessment and Tariff System, Warsaw, Poland; 81 Department of Physical Therapy, Faculty of Allied Health Sciences, Naresuan University, Meung District, Thailand; 82 Department of Epidemiology and Biostatistics, Tehran University of Medical Sciences, Tehran, Iran; 83 Ophthalmic Research Center, Shahid Beheshti University of Medical Sciences, Tehran, Iran; 84 School of International Development and Global Studies, University of Ottawa, Ottawa, Ontario, Canada; 85 School of Public Health, Bahir Dar University, Bahir Dar, Ethiopia; 86 Health Economics & Finance, Jackson State University, Jackson, Mississippi, USA; 87 Research Center for Public Health, Tsinghua University, Peking, China; 88 Health Promotion Research Center, Iran University of Medical Sciences, Tehran, Iran; 89 Indian Institute of Public Health, Public Health Foundation of India, Gurugram, India; 90 Department of Health Metrics Sciences, School of Medicine, University of Washington, Seattle, Washington, USA; 91 School of Population and Global Health, University of Melbourne, Melbourne, Queensland, Australia

**Keywords:** burden of disease, motorcycle, descriptive epidemiology, road traffic accident, road injuries, pedestrian injuries, traumatic brain injury

## Abstract

**Background:**

The global burden of road injuries is known to follow complex geographical, temporal and demographic patterns. While health loss from road injuries is a major topic of global importance, there has been no recent comprehensive assessment that includes estimates for every age group, sex and country over recent years.

**Methods:**

We used results from the Global Burden of Disease (GBD) 2017 study to report incidence, prevalence, years lived with disability, deaths, years of life lost and disability-adjusted life years for all locations in the GBD 2017 hierarchy from 1990 to 2017 for road injuries. Second, we measured mortality-to-incidence ratios by location. Third, we assessed the distribution of the natures of injury (eg, traumatic brain injury) that result from each road injury.

**Results:**

Globally, 1 243 068 (95% uncertainty interval 1 191 889 to 1 276 940) people died from road injuries in 2017 out of 54 192 330 (47 381 583 to 61 645 891) new cases of road injuries. Age-standardised incidence rates of road injuries increased between 1990 and 2017, while mortality rates decreased. Regionally, age-standardised mortality rates decreased in all but two regions, South Asia and Southern Latin America, where rates did not change significantly. Nine of 21 GBD regions experienced significant increases in age-standardised incidence rates, while 10 experienced significant decreases and two experienced no significant change.

**Conclusions:**

While road injury mortality has improved in recent decades, there are worsening rates of incidence and significant geographical heterogeneity. These findings indicate that more research is needed to better understand how road injuries can be prevented.

## Introduction

In the original 1971 formulation of the epidemiological transition, Abdel Omran suggested that a country could be expected to pass through three phases of health loss patterns as its economy improved.^1^ A country would experience, first, an ‘age of pestilence and famine’ and, second, an ‘age of receding pandemics’. The third phase would include increased burden from ‘degenerative and man-made diseases’, a phase that in their 2002 review Salomon and Murray summarised as health loss from ‘cancers, cardiovascular diseases, and accidents’.^2^ This work on the epidemiological transition provides a starting point for reviewing the current global burden of road injuries and for investigating the relationship between road injuries and economic development. The burden of road injuries has become an area of particular focus across global forums in recent years. In March 2010, the United Nations (UN) General Assembly proclaimed 2011–2020 as the Decade of Action for Road Safety.^3^ In 2015, the UN General Assembly established Sustainable Development Goal 3.6 as the target of reducing road traffic deaths and injuries by 50% by 2020.^4^ More recently, the WHO published the Global Status Report on Road Safety 2018 and established focus on road safety goals with performance targets in the WHO’s General Programme of Work 2019–2023.^5^ Efforts such as Vision Zero have developed cross-setting efforts ranging from countries in Europe to states in India to cities in the USA to develop a road safety paradigm focused on reducing road injury burden to zero.^6^ The European Transport Safety Council has developed evidence-based guidance on transport safety improvements in Europe, while the Insurance Institute for Highway Safety in the USA has conducted research on the science of highway safety and on safety profiles of different vehicles. Globally, the International Transport Forum has developed important resources to guide transport safety improvements on a global basis across multiple modes of transport. The complexity of road safety science has advanced such that entire textbooks now focus on the elements of road safety ranging from behavioural science to economic relationships.^7^ Across these efforts, it is evident that it is now more critical than ever for legislative policymakers, ministries of health, transportation sectors, academic research groups and other agencies to work collaboratively with a Safe System paradigm on improving road safety.^8^ Measurement of road injury burden is a critical component of advancing these initiatives.

Many other studies have measured road injury burden using different methods and data sources including updates to the Global Burden of Disease (GBD) Study, road safety reports by the WHO and reports or studies published by other groups.^9–12^ While past research has been instrumental in advancing road safety initiatives, it is also important to produce regular updates of road injury burden estimates. Updates that include recent years are critical to ensuring that the effects of economic development, new policies and new safety technologies can be observed and discussed with minimal latency. Timeliness of updating road injury burden estimates helps ensure that policymakers and health resources researchers appropriately focus their efforts, and historically evidence-informed policies regarding road injuries have been impactful. For example, research on road injury burden in Iran in the early 2000s led to new policies being enacted to address the growing burden, while elsewhere in countries such as the USA and Australia, legislation focused on intoxicated driving, seatbelt requirements, speed controls and vehicle safety have likely contributed to decreasing mortality rates from road injuries in select areas.^13–16^ In cases where road safety legislation has been passed, successful implementation of such policies is also critical, and it is also not clear the extent to which successful policy in one location can be equally successful elsewhere. Road injuries are a unique cause of morbidity and mortality on the global landscape because unlike diseases and injuries for which there may be considerable lag between burden measurement, policy implementation and burden improvement, road injury burden can change rapidly if measures such as seatbelt laws, intoxicated driving laws and infrastructure improvements are implemented.^17–20^ Hence, it is important to continue regular updates of health assessments that measure morbidity and mortality from road injuries, as preventing and treating road injuries is of critical importance for sustainable improvements in population health outcomes and warrants detailed analysis to understand sociodemographic patterns as well as geographical trends over time.

The Global Burden of Diseases, Injuries, and Risk Factors Study (GBD) is a comprehensive assessment of health loss to measure morbidity and mortality from a wide array of diseases, injuries and risk factors.^11 12 21–24^ The study involves a global network of over 3500 collaborators who provide broad expertise on diseases, injuries, risk factors and locations. The study is published on an annual basis, so estimates are frequently updated with new input data and methodological improvements. GBD 2017 was published in 2018 and included road injuries as one of 30 mutually exclusive, collectively exhaustive injury-related causes of death and disability. In the GBD, road injuries encompass injuries involving motor vehicles, pedestrians, motorcyclists and cyclists. GBD 2017 included estimates of road injury morbidity and mortality in terms of incidence, prevalence, years lived with disability (YLDs), cause-specific mortality, years of life lost (YLLs) and disability-adjusted life years (DALYs) for 195 countries and territories, all age groups and both sexes, for years between 1990 and 2017.

The objective of this paper is to use the GBD 2017 results and framework to provide an updated assessment of the global burden of road injuries and to identify trends and patterns that may be useful by policymakers, organisations and the private sector for preventing future road injury burden.

## Methods

### GBD 2017

GBD 2017 methods and results are described in extensive detail in GBD literature, including descriptions of the analytical estimation framework used to measure deaths, YLLs, incidence, prevalence, YLDs and DALYs for every cause in GBD including injuries.^11 12 21–24^ A review of key GBD methods is summarised in online supplementary appendix 1. The methodological components specific to injuries and road injuries estimation within the GBD framework are as follows. All key analytical steps are conducted across 1000 draws, and the ordered 25th and 975th values of the final estimates are used to determine the 95% uncertainty interval (UI).

10.1136/injuryprev-2019-043302.supp1 Supplementary data

### GBD injury classification

Our case definition for a road injury is ‘interaction, as a pedestrian on the road, with an automobile, motorcycle, pedal cycle, or other vehicles resulting in bodily damage or death’. The GBD cause hierarchy includes road injuries as an external cause of injury, similar to falls or poisoning. These external cause-of-injury codes or ‘E codes’ are designated as mutually exclusive and collectively exhaustive in the cause hierarchy, meaning that they include every possible cause of death or disability either as specific injuries or as residual (‘other’) injuries. These external cause-of-injury codes cause nature-of-injury codes, which specify the bodily injury that is caused by an external cause of injury. In terms of the nature-of-injury codes (eg, the traumatic brain injury (TBI) that might be due to a road injury), injuries were categorised into 47 mutually exclusive and collectively exhaustive nature-of-injury categories using chapters S and T in the International Classification of Diseases (ICD), 10th revision, and codes 800–999 in ICD-9. Since it is possible that an external cause of injury including a road injury may not actually lead to bodily harm, we only include injuries in our morbidity analysis that warranted some form of healthcare, which is typically indicated in survey data for road injuries and can be inferred from our use of hospital records. For example, a low-speed collision (‘fender bender’) that did not lead to any bodily injury to drivers, passengers or bystanders would not be considered an injury in GBD.

### Mortality and YLLs from road injuries

GBD methods for cause of death estimation is provided in GBD literature.^11 12 21–25^ A brief overview of this process is as follows. First, all available data sources were accessed and mapped into the GBD cause list and cause hierarchy. Road injuries data sources included vital registration, verbal autopsy studies, mortality surveillance, censuses, surveys, hospital records and mortuary data. For road injuries, we used ICD-9 codes E800.3, E801.3, E802.3, E803.3, E804.3, E805.3, E806.3, E807.3, E810.0-E810.6, E811.0-E811.7, E812.0-E812.7, E813.0-E813.7, E814.0-E814.7, E815.0-E815.7, E816.0-E816.7, E817.0-E817.7, E818.0-E818.7, E819.0-E819.7, E820.0-E820.6, E821.0-E821.6, E822.0-E822.7, E823.0-E823.7, E824.0-E824.7, E825.0-E825.7, E826.0-E826.1, E826.3-E826.4, E827.0, E827.3-E827.4, E828.0, E828.4 and E829.0-E829.4, and ICD-10 codes V01-V04.99, V06-V80.929, V82-V82.9 and V87.2-V87.3. Second, we redistributed ill-defined causes of death to specific underlying causes, including road injuries, via a process known as garbage code redistribution.^12 26^ Third, ensemble models for road injuries and each subtype were conducted using the GBD Cause of Death Ensemble modelling (CODEm) software. CODEm employs five principles to build a cause of death model based on testing a variety of possible models that have been run through several modelling classes using an array of covariates.^27^ Next, an ensemble of best-performing models is constructed based on out-of-sample validity testing. The covariates used in the models included lag-distributed income per capita (a smoothed series of GDP per capita), education per capita in years, alcohol use in litres per capita, an indicator for opium cultivation, population density over 1000 per square kilometre, a summary exposure value for violent injuries, Socio-demographic Index (SDI) and Healthcare Access and Quality Index. Deaths for each cause are then rescaled such that the sum of deaths across causes equals the total deaths, which enforces internal consistency across GBD estimates. As a final step, YLLs due to road injuries and each subtype are calculated by multiplying deaths by the residual life expectancy at the age of death from GBD 2017 standard model life table. YLLs measure the number of years of life are lost when a death occurs at an age less than the life expectancy; for example, if the residual life expectancy at age 25 years is 60, then 60 years of life were lost when a person dies at age 25 years.

### Incidence, prevalence and YLDs due to road injuries

Estimation of non-fatal injury outcomes (incidence, prevalence and YLDs) in GBD is described in detail in related publications.^11^ A summary is as follows. We used DisMod-MR 2.1 (a descriptive epidemiological meta-regression tool) to model incidence data for road injuries from emergency department and hospital records and survey data to estimate incidence by location, year, age and sex. These models were conducted for each subtype of road injuries. We used cause-specific mortality rates and incidence data to compute excess mortality rates following an injury since DisMod-MR 2.1 functions in a compartmental framework such that all incident cases of injury must be explained by dying, remaining prevalent or going into remission. Our assumption that case fatality rates are higher in lower income setting is implemented by adding lag-distributed income per capita as a covariate on excess mortality, which causes a negative relationship between income and mortality. This assumption is based on the observation that more sophisticated forms of treatment such as intensive care units (ICU), ventilator support and surgery may be required for higher acuity injuries resulting from road injuries.

After incidence cause models were conducted for each type of road injury, we split the cause incidence into inpatient and outpatient incidence based on a coefficient derived in DisMod-MR 2.1 in locations that had both types of data. Both of these series then went through the following steps. We developed a severity hierarchy of nature-of-injury types by using pooled datasets of follow-up studies from China, the Netherlands and the USA where health status 1 year after injury could be mapped to existing GBD disability weights.^28–34^ This severity hierarchy was used to identify the injury that would cause the most disability in the event that a road injury lead to multiple types of injuries (eg, a spinal cord transection and a wrist fracture).

Next, recognising that injury disability is determined by nature of injury rather than cause of injury, we estimated the proportion of road injuries that would lead to each nature-of-injury type being the most severe. We computed these proportions using Dirichlet regression methods in dual-coded hospital and emergency department data where both cause and nature could be identified. This process and the data sources used are described in more detail in other GBD studies.^35^ Each cause–nature matrix was specific to hospital admission versus injury warranting other healthcare, high/low income countries and territories, male/female and age category. Deriving these matrices separately in this manner allows variation by these variables. We then applied these proportions to our cause-of-injury incidence from DisMod-MR 2.1 in order to estimate cause–nature incidence. We converted these estimates to prevalence using the average duration for each nature of nature of injury and for inpatient and outpatient injuries from the Dutch Injury Surveillance System with supplementation from expert-driven estimates of short-term duration for nature of injury categories that had insufficient numbers in the Dutch dataset and for untreated injuries.^31^ We measured the probability of long-term (permanent) disability to account for the permanence of conditions such as spinal cord injury as opposed to the shorter term recovery for conditions such as a fibular fracture. The probability of long-term disability was based on analysis of long-term follow-up studies.^28–34^ Long-term prevalence was then calculated based on the ordinary differential equation solver used in DisMod-MR 2.1 to incorporate the parameters of incidence and long-term mortality risk for nature-of-injury conditions with increased mortality risk (eg, traumatic brain injury) such that prevalence is correctly estimated after accounting for excess mortality risk. Finally, we calculated YLDs by multiplying the prevalence of a health state, as defined in this process as the nature of injury, and a disability weight, which has been mapped to these injuries in previous GBD research.^36^ Finally, across all causes in GBD, a comorbidity correction is applied to account for comorbidity distributions in the population.^11^

### Socio-demographic Index

SDI is an indicator based on the human development index that includes income per capita, average educational attainment and total fertility rate under 25. Low SDI values correspond to low income per capita, low educational attainment and high fertility under 25 years, while high values correspond to higher income per capita, greater educational attainment and lower fertility under 25 years. We tabulate some results in this study by SDI quintile in order to identify socioeconomic patterns in road injury burden.

### Guidelines for Accurate and Transparent Health Estimates Reporting (GATHER) compliance

This study complies with the GATHER recommendations (see online supplementary appendix 2). Analyses were completed using Python version 2.7, Stata V.13.1 or R version 3.3. Statistical code used for GBD estimation is publicly available online at healthdata.org.

10.1136/injuryprev-2019-043302.supp2 Supplementary data

## Results

Summary results are as follows. Additional results by age, sex, year, location and injury cause and nature are available online at healthdata.org. Online resources also allow for measuring changes between different years, for example, between 2007 and 2017 as opposed to 1990 and 2017 as well as reviewing sources of data used in GBD 2017.

### Incidence

**Figure 1:**
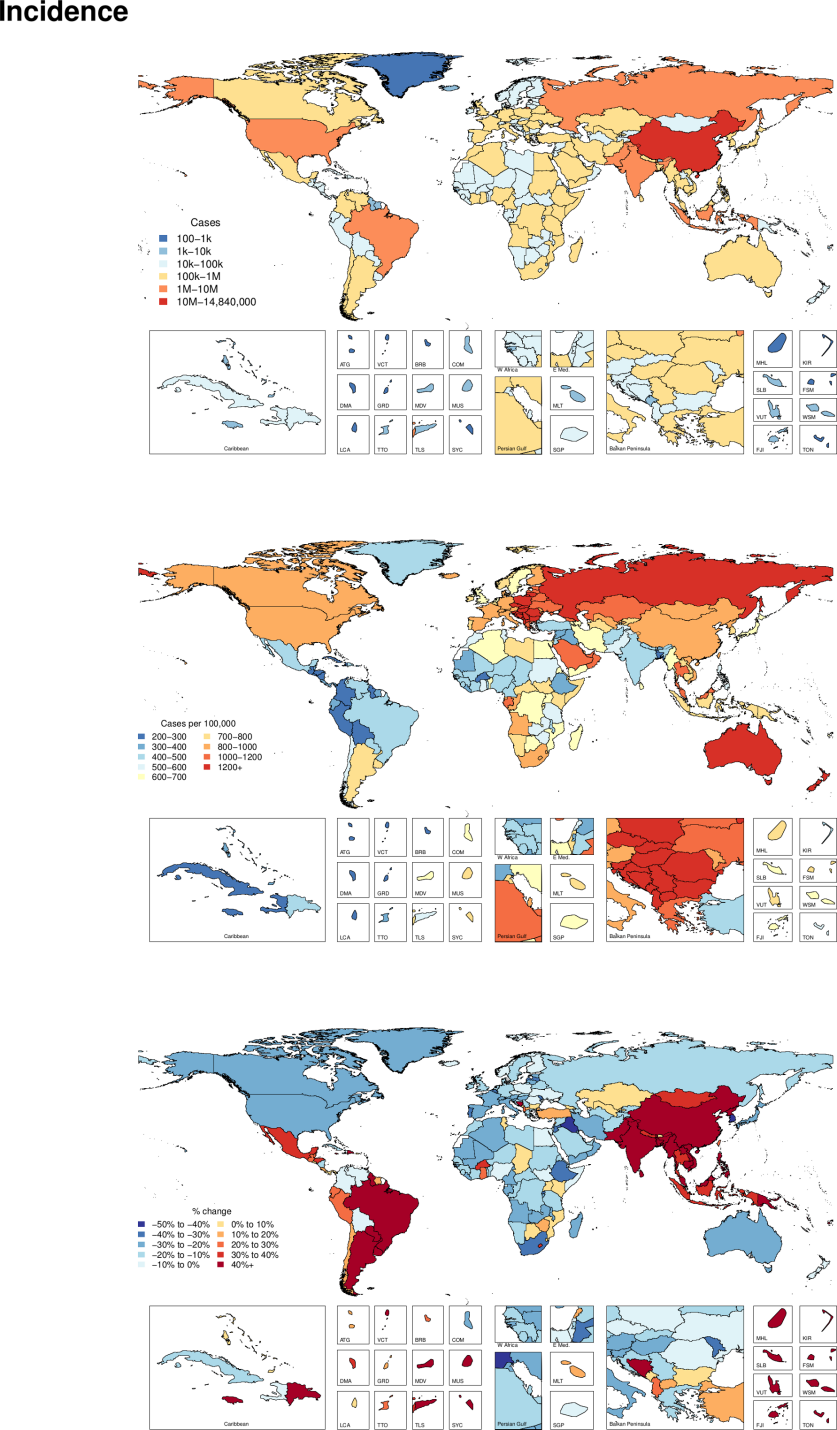
Incident cases, age-standardised incidence rates, and per cent change between 1990 and 2017 by country for road injuries.

Online supplementary appendix table 1 shows all ages incidence counts and age-standardised incidence rates for 2017 as well as the percentage change in age-standardised rates from 1990 to 2017 for overall road injuries. Countries in the middle SDI quintile experienced the highest increase of incidence rates from 1990 to 2017, with a 53.3% (95% UI 47.1 to 59.4) increase. High SDI was the only quintile that had decreased incidence rates during that time period, with a decrease of 16.5% (11.9 to 21.0). Figure 1 shows the new cases and age-standardised incidence rates of road injuries for 2017 and the per cent change between 1990 and 2017 for age-standardised incidence rates by country and territory. Globally, the age-standardised incidence rate was 692 (605 to 786) per 100 000 in 2017, representing an increase of 11.3% (6.4 to 15.8) from 1990 to 2017 and corresponding to 54 192 330 (47 381 583 to 61 645 891) new cases in 2017. Age-standardised incidence rates decreased from 1990 to 2017 in 109 out of 195 countries and territories, with the largest declines in South Korea, Iraq and Portugal, which decreased by 40.6% (33.3 to 46.6), 40.4% (34.5 to 45.2) and 38.8% (31.9 to 45.5), respectively.

10.1136/injuryprev-2019-043302.supp3 Supplementary data

**Figure 2:**
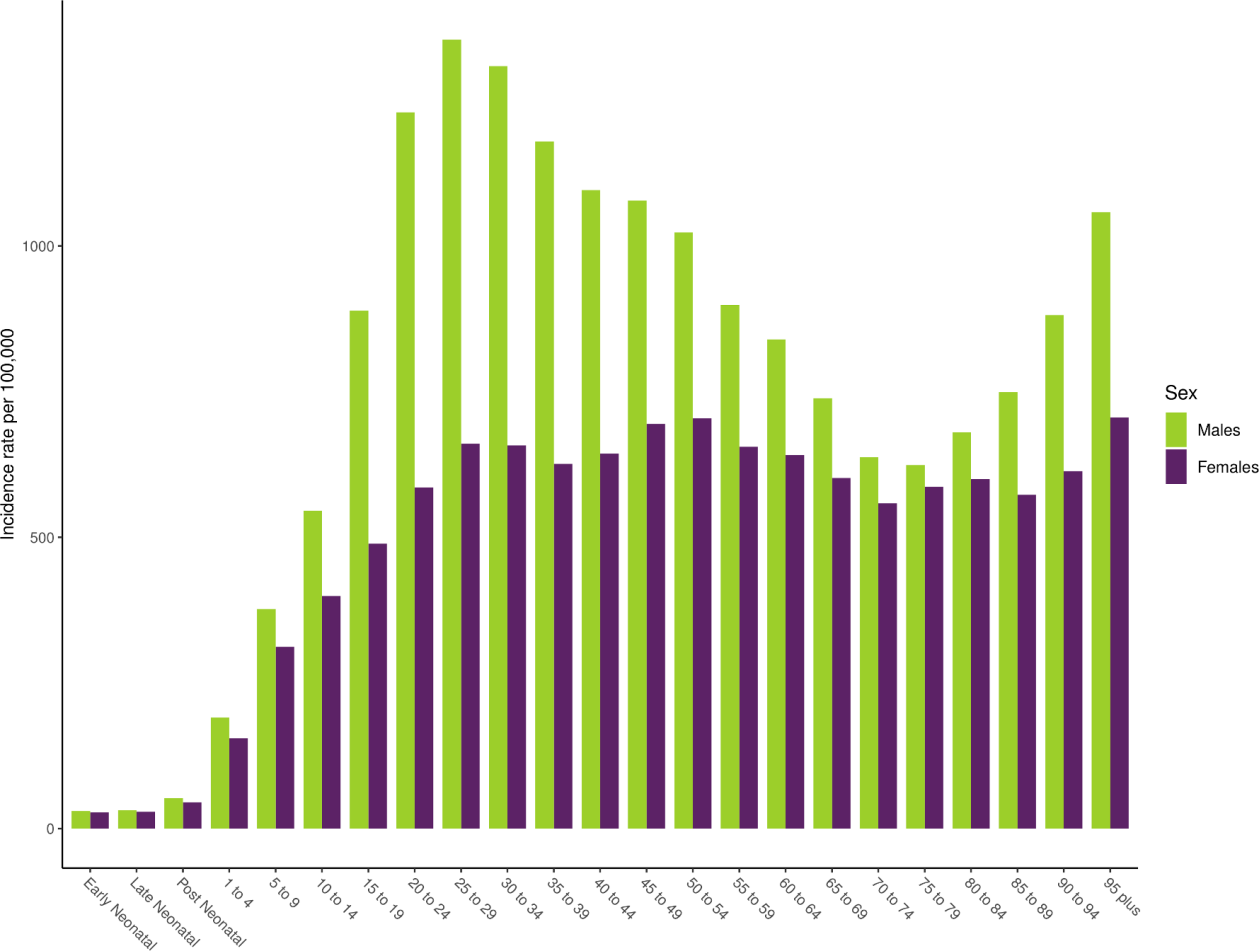
Age-specific and sex-specific incidence of road injuries globally in 2017.

The regions with the highest age-standardised incidence rates in 2017 were Central Europe (1467 (1297 to 1687)), Australasia (1304 (1157 to 1480)) and Eastern Europe (1193 (1022 to 1405)). Among the 21 GBD regions, 10 experienced significant decreases in age-standardised incidence rates, 9 regions experienced significant increases in age-standardised incidence rates (with the greatest increases found in East Asia and Oceania) and the remaining two regions experienced no significant change in age-standardised incidence rates (Central Europe and Central Asia). Age-standardised incidence rates decreased the most from 1990 to 2017 in High-income Asia Pacific, decreasing by 28.3% (23.5 to 33.2) and had the greatest increase in East Asia, where it increased by 111.2% (101.4 to 120.8). In terms of an age pattern, figure 2 shows global age-specific incidence rates for each age group by sex in 2017. This figure emphasises how road injury incidence is heavily concentrated in young to middle age groups and that males experience higher incidence rates than females, particularly in young adulthood.

### Cause-specific mortality

**Figure 3:**
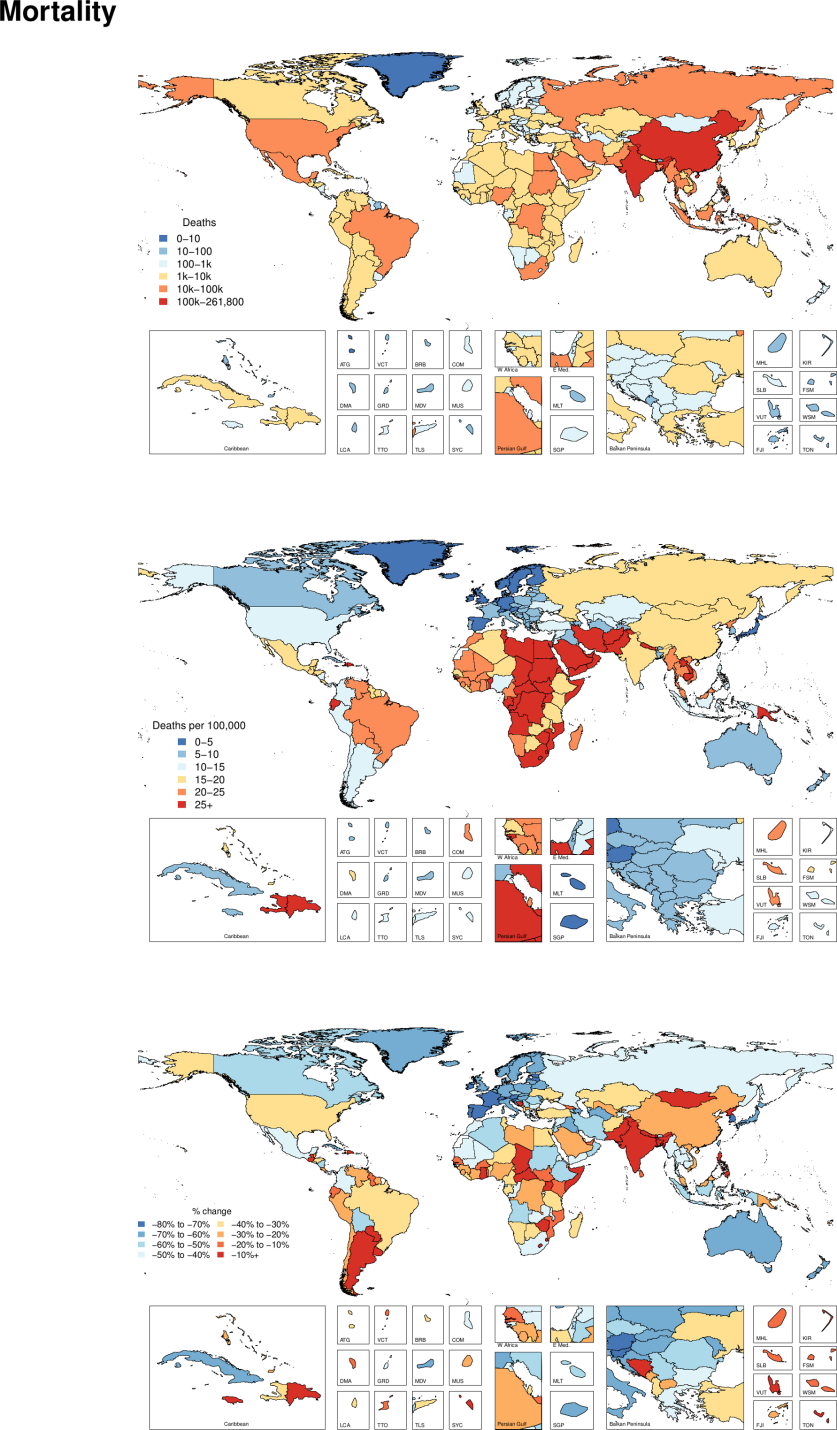
Deaths, age-standardised mortality rates and per cent change between 1990 and 2017 by country for road injuries.

Online supplementary appendix table 2 shows all ages deaths and age-standardised mortality rates for 2017 as well as the percentage change in age-standardised rates from 1990 to 2017. Globally, the age-standardised mortality rate was 15.8 (15.2 to 16.3) per 100 000 in 2017, which corresponded to 1 243 068 (1 191 889 to 1 276 940) deaths in 2017 and represented a 29.0% (25.0 to 33.6) decrease in age-standardised mortality rate from 1990 to 2017. Geographically, figure 3 shows the deaths and age-standardised mortality rate from road injuries in 2017 and the per cent change between 1990 and 2017. This figure reveals the general pattern that mortality rates from road injuries is highest in select countries in North Africa, the Middle East and Southern sub-Saharan Africa in 2017. The countries with the highest age-standardised mortality rates were Central African Republic (85.5 (50.7 to 111.2) deaths per 100 000), Somalia (51.1 (27.8 to 72.0)) and United Arab Emirates (49.9 (39.5 to 61.1)). China had the highest number of total deaths, with 261 802 (247 924 to 273 651) deaths estimated in 2017.

10.1136/injuryprev-2019-043302.supp4 Supplementary data

### YLDs, YLLs and DALYs

Online supplementary appendix table 3 shows the counts, age-standardised rates and per cent change from 1990 to 2017 of YLDs, YLLs and DALYs for road injuries. Globally, in 2017, road injuries resulted in 57 638 366 (55 500 786 to 59 369 191) YLLs, 10 159 667 (7 272 042 to 13 618 818) YLDs and 67 798 033 (64 337 599 to 71 454 968) DALYs, reflecting age-standardised rates of 745 (718 to 767) per 100 000, 126 (90 to 169) and 871 (828 to 917), respectively. Age-standardised YLLs and DALYs decreased by 34.4% (30.4 to 38.5) and 30.8% (26.9 to 35.0), respectively, between 1990 and 2017, while age-standardised YLDs increased 2.2% (0.3 to 4.0). The region with the highest age-standardised DALY rate was Central sub-Saharan Africa with 1720 (1448 to 1999) DALYs per 100 000, which represented 1564 (1302 to 1834) YLLs and 156 (114 to 204) YLDs.

10.1136/injuryprev-2019-043302.supp5 Supplementary data

### Mortality-to-incidence ratios (MIRs)

**Figure 4:**
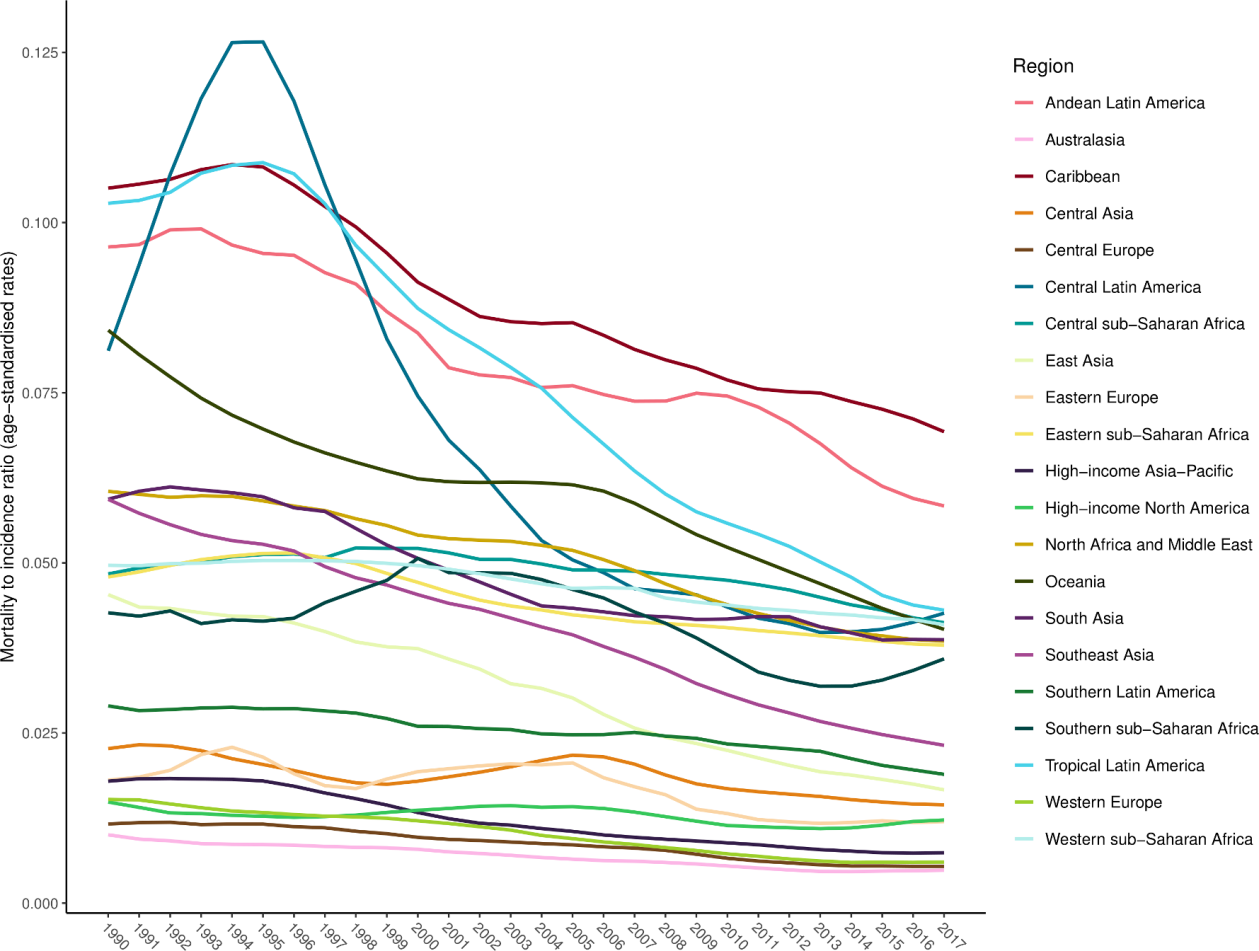
Changes in mortality-to-incidence ratios by GBD region from 1990 to 2017. GBD, Global Burden of Disease.

Figure 4 shows the ratios of age-standardised mortality rates to age-standardised incidence rates by region in 1990 and 2017, which approximates the risk of death given a road injury. This figure shows how the MIRs vary by both time and location. The Caribbean had the highest MIR in 2017, while Australasia had the lowest, following the pattern of percentage DALYs caused by YLDs described above. While MIR varied substantially across regions, it also declined in every region from 1990 to 2017.

### Nature of injuries caused by road injuries

**Figure 5:**
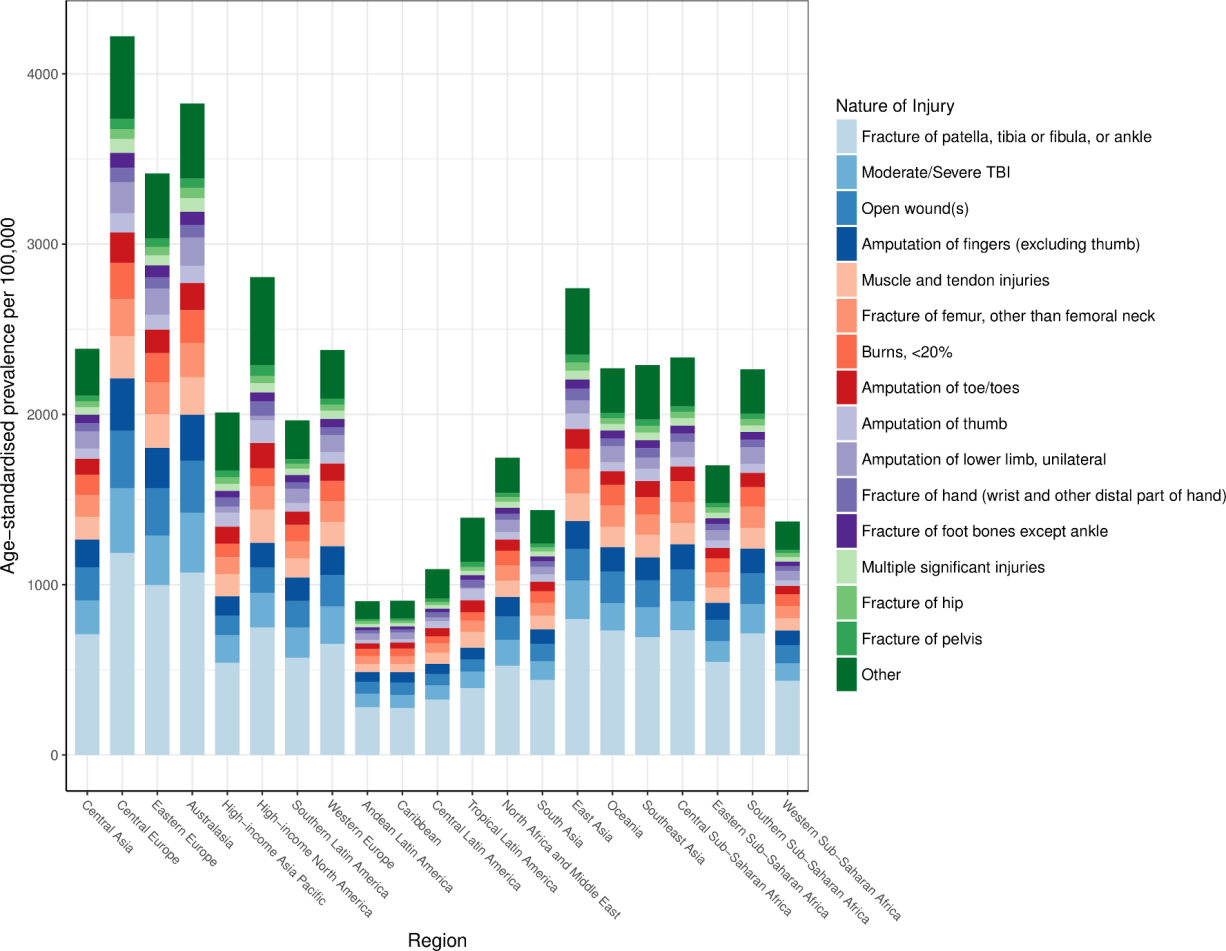
Distribution of most severe nature of injury sustained in road injuries by region in 2017. TBI, traumatic brain injury.

The average global disability weight used in computing YLDs after comorbidity adjustment was 5.8%. Figure 5 shows the distribution of natures of injury in terms of age-standardised prevalence by region. This figure shows that the category of injury that includes fractures of patella, tibia or fibula or ankle is the leading cause of disability for victims of road injuries. TBI is also an important contributor to health loss from road injuries in all regions of the world.

## Discussion

The Global Status Report on Road Safety in 2018 published by the WHO cites important progress in road safety initiatives that have made at the country level, such as new legislation oriented to road safety, updated vehicle standards and technology and access to trauma care.^5^ For example, 123 out of 175 countries included in the report were noted to have best-practice road safety laws implemented for at least one of the key risk factors for road injuries, and the report notes progress such as additional countries passing legislation and policy related to drink driving, motorcycle helmet use and child restraint systems. In this study, we found that despite global increases in road injuries incidence between 1990 and 2017, cause-specific mortality has decreased over the same time period, which likely reflects many of these underlying country-level improvements as described by the WHO. From this summary finding, several important themes emerge.

First, the observation of incidence increasing and mortality decreasing on the global level implies that while road injuries are becoming more frequent, individuals experiencing road injuries are less likely to die. It is likely that at least part of the increases in incidence can be explained by broadly increasing access to and utilisation of motorised transport in all locations of the world over the time period of this study, including shifts in types of motorised transit (eg, from bicycles to motorbikes) being used. This observation may also imply general improvements in case fatality rates. Improvements in case fatality rates may be affected by two general processes. First, it is possible that improvements in infrastructure, driving laws such as seatbelt laws and vehicle safety improvements have led to the types of disability sustained in road injuries decreasing in severity over time. For example, a driver who was in a road incident in 1990 may have been less likely to be wearing a seatbelt than a driver in 2017, which could have increased the probability of more severe injuries and death in 1990 relative to 2017, all else being equal. Similarly, infrastructure improvements such as improved roads, guard rails and streetlights, particularly in developing economies, may have led to less morbidity and mortality in each road injury case, even if the total number of cases is increasing due to factors such as increased rates of driving.^37 38^ The second possible factor that could lead to improvements in case fatality is improvements in access to medical care following a road injury. For example, adding ambulance services, building trauma centres and ensuring access to emergency medical care in all populations is likely to be beneficial in terms of improving survival for road injury cases, which has been shown in locations that advance trauma systems.^39^ Advances in trauma care over the past three decades have led to improved imaging and diagnostic technologies being more readily available to global populations, and research in trauma resuscitation has led to better understanding of the pathology that can occur in a road injury, though postincident care in road injuries remains an ongoing area of research.^40 41^ It is likely that the implied improvements in case fatality have also been affected by improved quality and access to medical care on a global scale. Among SDI quintiles, one exception to this trend occurred in the highest SDI quintile, which experienced decreases both in incidence and mortality, suggesting that concomitant improvements may be possible as socioeconomic development continues globally.

Second, while we found global improvements in mortality despite increases in incidence, we also observed considerable heterogeneity by country and region. Despite global improvements in mortality, multiple countries experienced increases in age-standardised cause-specific mortality from road injuries during this study period. For example, Paraguay, Chad, Lesotho, Pakistan, Mongolia and North Korea experienced increases in road injuries mortality, emphasising that despite global improvements, it is important for health policy research to be conducted in areas where fatal burden from road injuries is still increasing. For example, patients with moderate-to-severe injury that received treatment at a level 1 trauma centre in the USA were shown to be at a 25% decreased risk of death when compared with those who accessed a non-trauma centre, raising the question of whether medical infrastructure development could produce similar improvements in lower income settings.^40^ It is possible that portions of road injury burden may be mitigated by legislation (eg, seatbelt laws), infrastructure and engineering (eg, road construction) and behavioural modifications (eg, intoxicated driving). Yet it is also likely that there are still unidentified factors that lead to road injury incidence and mortality, particularly as these trends are likely governed by a wide array of factors ranging from trauma pathophysiology to vehicle engineering to social behaviours. Future road injury research may benefit from more comprehensive syntheses of how various causes and modifiers affect these outcomes, similar to how our understanding of cancer, infectious disease and cardiovascular disease has benefited from laboratory-based, translational-based and population-based research studies.

Third, we found that changes in incidence and mortality varied by development. Specifically, countries and territories in the middle SDI quintile experienced the greatest increases in age-standardised incidence between 1990 and 2017, while low SDI quintile locations increased less, and high SDI quintile locations actually decreased in terms of incidence. These findings are reminiscent of the transition phases described in literature on the epidemiological transition, where a country’s burden of disease and injury is modulated by where the country is on the development spectrum. For example, Papua New Guinea and Myanmar, low and low-middle SDI countries, respectively, have experienced significant economic growth in the past decade.^42^ Both countries have also experienced increased incidence of road injuries over the past 10 years, while the burden of communicable diseases decreased. These country experiences support the idea that while countries transition to more stable economies, road injuries predictively become more burdensome. Interestingly, there is evidence that reductions in road traffic injuries have positive effects on GDP per capita, so there is incentive for developing countries to prioritise road safety initiatives and injury prevention.^43^

Fourth, for the first time in GBD research, we were able to estimate the burden of road injuries in terms of the types of disability that road injuries caused. Specifically, we found that the most common nature of injury sustained in a road injury in all regions was fracture of patella, tibia/fibula or ankle and that in most regions, moderate/severe TBIs were the next leading cause of disability in road injuries. These are important findings for two reasons. Lower extremity fractures can require surgical management in order to avoid longer term disability, which emphasises the importance of modern medical services including surgical services being available in all areas of the world. In addition, these findings show how disability from road injuries can lead to lifelong health loss in the form of conditions like TBI that can have irreversible health consequences, emphasising the importance of preventative strategies in reducing future burden from road injuries.

There were several limitations to this study. First, similar to other analyses in GBD research, the uncertainty of road injury morbidity and mortality rates is affected by data availability. In countries and territories with absent or sparse data, the modelling framework relies more on covariates and other locations that do have data, which leads to greater uncertainty around the point estimates. Greater UIs mean that readers and policymakers should use more caution when acting on these results. To address this limitation, health systems in the future should prioritise good data collection strategies in order to help improve the accuracy of future research in road injury burden. Current data limitations, modelling differences and garbage code redistribution, particularly for data-sparse or data-absent locations likely account for much of the difference between global mortality estimates from the WHO, which estimated 1.35 million deaths in 2016, and GBD 2017, which estimated 1.25 million deaths in 2016. Second, as described in other GBD literature on injury estimation, the current process for assigning disability to a road injury case requires predicting the most disabling injury that results from a road injury, without taking into consideration the possibility that multiple natures of injury can result from a road injury. In future GBD research, developing methods to capture all forms of disability that result from road injuries could help measure the total health loss burden from these conditions. Finally, a general limitation of non-fatal injury estimation in GBD 2017 was that long-term follow-up studies used for injury severity hierarchies and probabilities of long-term disability are only available in select countries. Future GBD updates should focus on adding more data to inform this analytical process.

## Conclusion

This study further substantiates the key messages highlighted in the Global Status Report on Road Safety 2018 by the WHO. In particular, despite improvements in mortality, road injuries remain critically important cause of morbidity and mortality globally, and more research is needed to better measure and understand how road injuries can be prevented, particularly in developing economies. Investing in preventative measures as well as ensuring that victims of road injuries have access to first response trauma and medical care could help drive improvements in road injury burden in the future.

**What is already known on the subject**

- Road injuries are known to be a major cause of health loss globally, both in terms of morbidity and mortality.
- While progress on mitigating health loss from road injuries has been made in some locations, there is still considerable morbidity and mortality in all areas of the world, including in low-income and middle-income regions.

**What this study adds**

- Road injury incidence has increased globally from 1990 to 2017, while mortality has decreased.
- Trends in mortality-to-incidence ratios for road injuries have varied depending on region of the world between 1990 and 2017.
- The specific type of bodily injury occurring in road injuries is now estimated, with the most common nature of injury sustained in road injuries being a fracture of the patella, tibia or fibula, or ankle.

## References

[1] Omran AR The epidemiologic transition. A theory of the epidemiology of population change. Milbank Mem Fund Q 1971;49:509–38. 10.2307/3349375 5155251

[2] Salomon JA, Murray CJL The epidemiologic transition revisited: compositional models for causes of death by age and sex. Popul Dev Rev 2002;28:205–28. 10.1111/j.1728-4457.2002.00205.x

[3] WHO Decade of action for road safety 2011-2020. WHO. Available: http://www.who.int/roadsafety/decade_of_action/en/ [Accessed 27 Jul 2019].

[4] Sustainable Development Goals Sustainable development knowledge platform. Available: https://sustainabledevelopment.un.org/?menu=1300 [Accessed 27 Jul 2019].

[5] WHO Global status report on road safety 2018. WHO. Available: http://www.who.int/violence_injury_prevention/road_safety_status/2018/en/ [Accessed 26 Jul 2019].

[6] Kim E, Muennig P, Rosen Z Vision zero: a toolkit for road safety in the modern era. Inj Epidemiol 2017;4 10.1186/s40621-016-0098-z PMC5219975 28066870

[7] Shinar D Traffic safety and human behavior. 2nd edn Emerald Group Publishing, 2017.

[8] The Safe System - Towards Zero Foundation. Available: http://www.towardszerofoundation.org/thesafesystem/ [Accessed 27 Jul 2019].

[9] WHO Road traffic injuries publications and resources. WHO. Available: http://www.who.int/violence_injury_prevention/publications/road_traffic/en/ [Accessed 27 Jul 2019].

[10] Peden M, Scurﬁeld R World report on road traffic injury prevention. Geneva: World Health Organization, 2004.

[11] James SL, Abate D, Abate KH, et al Global, regional, and national incidence, prevalence, and years lived with disability for 354 diseases and injuries for 195 countries and territories, 1990–2017: a systematic analysis for the global burden of disease study 2017. Lancet 2018;392:1789–858. 10.1016/S0140-6736(18)32279-7 30496104 PMC6227754

[12] Roth GA, Abate D, Abate KH, et al Global, regional, and national age-sex-specific mortality for 282 causes of death in 195 countries and territories, 1980–2017: a systematic analysis for the global burden of disease study 2017. Lancet 2018;392:1736–88. 10.1016/S0140-6736(18)32203-7 30496103 PMC6227606

[13] The burden of disease and injury in Iran 2003 | Population health metrics | Full text. Available: https://pophealthmetrics.biomedcentral.com/articles/10.1186/1478-7954-7-9 [Accessed 5 Feb 2019]. 10.1186/1478-7954-7-9 PMC2711041 19527516

[14] Trends of fatal road traffic injuries in Iran (2004–2011). Available: https://www.ncbi.nlm.nih.gov/pmc/articles/PMC3665536/ [Accessed 5 Feb 2019]. 10.1371/journal.pone.0065198 PMC3665536 23724132

[15] Achievements in public health, 1900-1999 motor-vehicle safety: a 20th century public health achievement. Available: https://www.cdc.gov/mmwr/preview/mmwrhtml/mm4818a1.htm [Accessed 10 May 2019].

[16] McDermott FT, Hough DE Reduction in road fatalities and injuries after legislation for compulsory wearing of seat belts: experience in Victoria and the rest of Australia. Br J Surg 1979;66:518–21. 10.1002/bjs.1800660721 466050

[17] Ameratunga S, Hijar M, Norton R Road-Traffic injuries: confronting disparities to address a global-health problem. Lancet 2006;367:1533–40. 10.1016/S0140-6736(06)68654-6 16679167

[18] Petridou E, Moustaki M Human factors in the causation of road traffic crashes. Eur J Epidemiol 2000;16:819–26. 10.1023/A:1007649804201 11297224

[19] Suriyawongpaisal P, Kanchanasut S Road traffic injuries in Thailand: trends, selected underlying determinants and status of intervention. Inj Control Saf Promot 2003;10:95–104. 10.1076/icsp.10.1.95.14110 12772492

[20] Global status report on road safety | Injury prevention. Available: https://injuryprevention.bmj.com/content/15/4/286.short [Accessed 5 Feb 2019].

[21] Dicker D, Nguyen G, Abate D, et al Global, regional, and national age-sex-specific mortality and life expectancy, 1950–2017: a systematic analysis for the global burden of disease study 2017. Lancet 2018;392:1684–735. 10.1016/S0140-6736(18)31891-9 30496102 PMC6227504

[22] Kyu HH, Abate D, Abate KH, et al Global, regional, and national disability-adjusted life-years (DALYs) for 359 diseases and injuries and healthy life expectancy (HALE) for 195 countries and territories, 1990–2017: a systematic analysis for the global burden of disease study 2017. Lancet 2018;392:1859–922. 10.1016/S0140-6736(18)32335-3 30415748 PMC6252083

[23] Murray CJL, Callender CSKH, Kulikoff XR, et al Population and fertility by age and sex for 195 countries and territories, 1950–2017: a systematic analysis for the global burden of disease study 2017. Lancet 2018;392:1995–2051. 10.1016/S0140-6736(18)32278-5 30496106 PMC6227915

[24] Stanaway JD, Afshin A, Gakidou E, et al Global, regional, and national comparative risk assessment of 84 behavioural, environmental and occupational, and metabolic risks or clusters of risks for 195 countries and territories, 1990–2017: a systematic analysis for the global burden of disease study 2017. Lancet 2018;392:1923–94. 10.1016/S0140-6736(18)32225-6 30496105 PMC6227755

[25] Haagsma JA, Graetz N, Bolliger I, et al The global burden of injury: incidence, mortality, disability-adjusted life years and time trends from the global burden of disease study 2013. Inj Prev 2016;22:3–18. 10.1136/injuryprev-2015-041616 26635210 PMC4752630

[26] Foreman KJ, Naghavi M, Ezzati M Improving the usefulness of US mortality data: new methods for reclassification of underlying cause of death. Popul Health Metr 2016;14:14 10.1186/s12963-016-0082-4 27127419 PMC4848792

[27] Foreman KJ, Lozano R, Lopez AD, et al Modeling causes of death: an integrated approach using CODEm. Popul Health Metr 2012;10:1 10.1186/1478-7954-10-1 22226226 PMC3315398

[28] Mackenzie EJ, Rivara FP, Jurkovich GJ, et al The national study on costs and outcomes of trauma. J Trauma 2007;63(6 Suppl):S54–67. 10.1097/TA.0b013e31815acb09 18091213

[29] Traumatic Brain Injury Follow-Up registry and surveillance of TBI in the emergency department (ED); notice of availability of funds. federal register, 2002 Available: https://www.federalregister.gov/documents/2002/05/08/02-11359/traumatic-brain-injurytbi-follow-up-registry-and-surveillance-of-tbi-in-the-emergency-department-ed [Accessed 14 May 2018].

[30] China Zhuhai Study 2006-2007 - China CDC | GHDx. Available: http://ghdx.healthdata.org/record/china-zhuhai-study-2006-2007-china-cdc [Accessed 15 May 2018].

[31] Functional outcome at 2.5, 5, 9, and 24 months after injury in the Netherlands | GHDx. Available: http://ghdx.healthdata.org/record/functional-outcome-25-5-9-and-24-months-after-injury-netherlands [Accessed 15 May 2018].

[32] Health-Related quality of life after burns: a prospective multicentre cohort study with 18 months follow-up | GHDx. Available: http://ghdx.healthdata.org/record/health-related-quality-life-after-burns-prospective-multicentre-cohort-study-18-months-follow [Accessed 15 May 2018]. 10.1097/ta.0b013e3182199072 22439227

[33] Netherlands injury surveillance system 2007 | GHDx. Available: http://ghdx.healthdata.org/record/netherlands-injury-surveillance-system-2007 [Accessed 15 May 2018].

[34] Netherlands injury surveillance system 2010 | GHDx. Available: http://ghdx.healthdata.org/record/netherlands-injury-surveillance-system-2010 [Accessed 14 May 2018].

[35] GBD 2016 Traumatic Brain Injury and Spinal Cord Injury Collaborators Global, regional, and national burden of traumatic brain injury and spinal cord injury, 1990-2016: a systematic analysis for the global burden of disease study 2016. Lancet Neurol 2019;18:56–87. 10.1016/S1474-4422(18)30415-0 30497965 PMC6291456

[36] Salomon JA, Haagsma JA, Davis A, et al Disability weights for the global burden of disease 2013 study. Lancet Glob Health 2015;3:e712–23. 10.1016/S2214-109X(15)00069-8 26475018

[37] Odero W, Garner P, Zwi A Road traffic injuries in developing countries: a comprehensive review of epidemiological studies. Trop Med Int Health 1997;2:445–60. 10.1111/j.1365-3156.1997.tb00167.x 9217700

[38] Goniewicz K, Goniewicz M, Pawłowski W, et al Road accident rates: strategies and programmes for improving road traffic safety. Eur J Trauma Emerg Surg 2016;42:433–8. 10.1007/s00068-015-0544-6 26162937

[39] Gabbe BJ, Lyons RA, Fitzgerald MC, et al Reduced population burden of road transport-related major trauma after introduction of an inclusive trauma system. Ann Surg 2015;261:565–72. 10.1097/SLA.0000000000000522 24424142 PMC4337622

[40] MacKenzie EJ, Rivara FP, Jurkovich GJ, et al A national evaluation of the effect of trauma-center care on mortality. N Engl J Med 2006;354:366–78. 10.1056/NEJMsa052049 16436768

[41] Clement ND, Tennant C, Muwanga C Polytrauma in the elderly: predictors of the cause and time of death. Scand J Trauma Resusc Emerg Med 2010;18:26 10.1186/1757-7241-18-26 20465806 PMC2880283

[42] World Bank GDP per capita (current US$). Available: https://data.worldbank.org/indicator/NY.GDP.PCAP.CD?locations=US-MM&name_desc=false [Accessed 18 Mar 2019].

[43] World Bank The high toll of traffic injuries: unacceptable and preventable. World Bank, 2017.

