# Correction: *Morbidity and mortality from road injuries: results from the global Burden of Disease Study 2017*

**DOI:** 10.1136/injuryprev-2019-043302corr1

**Published:** 2020-09-28

James SL, Lucchesi LR, Bisignano C, *et al*. Morbidity and mortality from road injuries: results from the Global Burden of Disease Study 2017. *Inj Prev* [Epub ahead of print: 08 Jan 2020]. doi: 10.1136/injuryprev-2019-043302.

This article was previously published with errors in authorship.

Author Rakhi Dandona has been added prior to author Kebede Deribe. Affiliations for Rakhi Dandona are below:

Rakhi Dandona^1, 90, 92^

1 Institute for Health Metrics and Evaluation, University of Washington, Seattle, Washington, USA.

90 Department of Health Metrics Sciences, School of Medicine, University of Washington, Seattle, Washington, USA.

92 Public Health Foundation of India, Gurugram, India

