# Supplementary material for: Morbidity and mortality from road injuries: results from the Global Burden of Disease Study 2017
Source: Inj Prev. 2020 Jan 8;26(Suppl 1):i46–56. doi: 10.1136/injuryprev-2019-043302 (PMC7571357; doi:10.1136/injuryprev-2019-043302)
Supplement: Supplementary data [file injuryprev-2019-043302supp003.pdf]

Table 1: Incidence and prevalence for 2017 and percentage change of age-standardised rates between 1990 and 2017 by location for road injuries

Location	Incidence (95% UI)			Prevalence (95% UI)		
	2017 counts	2017 age-standardised rates per 100,000	Percentage change in age-standardised rates between 1990 and 2017	2017 counts	2017 age-standardised rates per 100,000	Percentage change in age-standardised rates between 1990 and 2017
**Global**	**54 192 330 (47 381 583 to 61 645 891)**	**692 (605 to 786)**	**11.3 (6.4 to 15.8)**	**174 209 559 (162 041 952 to 187 472 078)**	**2 162 (2 012 to 2 326)**	**6.1 (4.4 to 7.8)**
**Low SDI**	**5 375 667 (4 618 485 to 6 300 332)**	**441 (382 to 507)**	**5.1 (0.5 to 9.6)**	**12 631 012 (11 768 288 to 13 604 175)**	**1 350 (1 257 to 1 453)**	**3.1 (1.4 to 4.7)**
**Low-middle SDI**	**8 877 424 (7 650 756 to 10 260 632)**	**526 (456 to 601)**	**28.0 (22.4 to 33.4)**	**23 019 357 (21 410 093 to 24 775 486)**	**1 600 (1 488 to 1 723)**	**25.0 (23.4 to 27.1)**
**Middle SDI**	**14 929 437 (13 032 825 to 17 017 698)**	**675 (590 to 768)**	**53.3 (47.1 to 59.4)**	**46 704 019 (43 353 475 to 50 443 635)**	**2 030 (1 886 to 2 190)**	**46.9 (44.5 to 49.4)**
**High-middle SDI**	**14 383 420 (12 443 509 to 16 459 412)**	**958 (838 to 1 088)**	**26.1 (21.0 to 30.8)**	**48 571 716 (45 076 557 to 52 482 617)**	**2 801 (2 604 to 3 022)**	**17.0 (15.2 to 18.7)**
**High SDI**	**10 350 223 (9 153 450 to 11 670 460)**	**925 (811 to 1 072)**	**-16.5 (-21.0 to -11.9)**	**42 346 323 (39 300 706 to 45 846 127)**	**2 689 (2 498 to 2 913)**	**-15.8 (-17.0 to -14.7)**
**Central Europe, Eastern Europe, and Central Asia**	**4 923 859 (4 329 867 to 5 570 520)**	**1 170 (1 026 to 1 340)**	**-12.9 (-17.0 to -8.6)**	**18 465 790 (17 051 913 to 20 166 118)**	**3 461 (3 196 to 3 771)**	**-12.3 (-13.6 to -11.0)**
**Central Asia**	**759 348 (665 702 to 856 937)**	**818 (722 to 920)**	**-5.3 (-12.0 to 1.6)**	**2 078 416 (1 922 852 to 2 256 558)**	**2 385 (2 205 to 2 591)**	**-6.7 (-7.7 to -5.7)**
Armenia	20 268 (17 748 to 22 866)	654 (567 to 751)	-14.3 (-20.7 to -7.1)	69 543 (64 414 to 75 333)	1 877 (1 741 to 2 032)	-16.0 (-17.1 to -15.0)
Azerbaijan	72 627 (62 997 to 84 136)	685 (596 to 792)	-15.6 (-23.0 to -6.1)	213 505 (197 288 to 231 133)	1 986 (1 835 to 2 151)	-16.2 (-18.1 to -14.5)
Georgia	38 439 (34 200 to 42 558)	1 039 (919 to 1 158)	9.8 (1.8 to 18.0)	140 220 (129 273 to 153 114)	2 925 (2 696 to 3 188)	7.8 (6.3 to 9.2)
Kazakhstan	196 365 (172 735 to 219 475)	1 084 (953 to 1 218)	1.3 (-7.5 to 10.9)	564 509 (520 315 to 615 237)	3 078 (2 838 to 3 349)	-0.4 (-1.8 to 1.0)
Kyrgyzstan	50 019 (43 806 to 56 283)	788 (695 to 881)	-19.8 (-26.9 to -11.8)	126 026 (116 646 to 136 699)	2 284 (2 113 to 2 480)	-20.4 (-21.4 to -19.5)
Mongolia	31 285 (27 303 to 35 608)	934 (820 to 1 061)	37.6 (27.4 to 49.8)	83 458 (77 193 to 90 344)	2 751 (2 542 to 2 986)	30.5 (27.8 to 33.2)
Tajikistan	47 454 (41 170 to 55 096)	515 (448 to 588)	-17.6 (-24.5 to -10.3)	113 774 (105 809 to 122 835)	1 557 (1 449 to 1 682)	-17.3 (-18.5 to -16.2)
Turkmenistan	36 929 (31 858 to 43 047)	725 (628 to 842)	-12.2 (-20.3 to -3.5)	99 669 (91 974 to 108 293)	2 113 (1 951 to 2 293)	-13.9 (-15.0 to -12.6)
Uzbekistan	265 962 (231 341 to 303 579)	802 (704 to 911)	6.1 (-3.6 to 16.4)	667 711 (617 655 to 724 065)	2 293 (2 118 to 2 492)	2.9 (1.2 to 4.6)
**Central Europe**	**1 628 842 (1 458 069 to 1 819 420)**	**1 467 (1 297 to 1 687)**	**-7.2 (-14.4 to 0.4)**	**6 668 038 (6 185 225 to 7 288 830)**	**4 220 (3 904 to 4 618)**	**-8.0 (-9.0 to -6.9)**
Albania	36 354 (31 854 to 41 425)	1 286 (1 121 to 1 488)	26.4 (14.3 to 39.1)	128 825 (119 526 to 140 658)	3 692 (3 423 to 4 030)	21.7 (19.5 to 24.0)
Bosnia and Herzegovina	41 883 (37 038 to 47 354)	1 274 (1 116 to 1 484)	74.9 (63.0 to 85.8)	170 361 (157 649 to 186 256)	3 705 (3 421 to 4 054)	69.1 (65.5 to 73.6)
Bulgaria	95 174 (84 225 to 106 564)	1 435 (1 243 to 1 644)	6.1 (-4.9 to 17.1)	419 604 (388 565 to 459 091)	4 105 (3 792 to 4 496)	4.3 (2.5 to 6.1)
Croatia	66 672 (59 485 to 75 079)	1 654 (1 444 to 1 920)	-14.2 (-21.5 to -5.7)	284 734 (264 091 to 312 943)	4 749 (4 397 to 5 249)	-12.2 (-13.6 to -10.8)
Czech Republic	168 633 (149 243 to 187 385)	1 711 (1 487 to 1 951)	-2.5 (-12.0 to 6.9)	726 269 (673 716 to 791 332)	4 949 (4 574 to 5 402)	-0.5 (-2.5 to 1.3)
Hungary	118 866 (105 802 to 133 375)	1 284 (1 118 to 1 502)	-23.5 (-30.8 to -16.4)	504 484 (466 719 to 551 937)	3 700 (3 417 to 4 050)	-22.3 (-23.7 to -20.9)
Macedonia	26 616 (23 377 to 30 016)	1 244 (1 084 to 1 436)	16.3 (7.8 to 26.0)	101 669 (94 054 to 111 451)	3 606 (3 329 to 3 953)	12.8 (11.2 to 14.6)
Montenegro	8 790 (7 771 to 9 942)	1 412 (1 231 to 1 630)	5.3 (-3.8 to 15.6)	32 575 (30 218 to 35 540)	4 052 (3 751 to 4 430)	4.5 (3.0 to 5.9)
Poland	587 099 (523 266 to 657 285)	1 575 (1 385 to 1 812)	-8.4 (-17.2 to 1.1)	2 368 187 (2 197 814 to 2 588 866)	4 517 (4 179 to 4 949)	-8.8 (-10.1 to -7.3)
Romania	262 309 (234 312 to 291 214)	1 375 (1 207 to 1 574)	-8.1 (-15.6 to 1.9)	1 062 776 (984 963 to 1 160 111)	3 900 (3 613 to 4 263)	-10.4 (-12.2 to -8.7)
Serbia	110 270 (98 763 to 123 763)	1 261 (1 106 to 1 457)	-15.5 (-24.4 to -6.2)	428 949 (397 457 to 468 195)	3 611 (3 342 to 3 949)	-17.6 (-18.8 to -16.4)
Slovakia	74 154 (65 623 to 83 358)	1 418 (1 240 to 1 639)	-20.0 (-29.2 to -9.0)	296 765 (274 969 to 325 244)	4 099 (3 789 to 4 494)	-19.0 (-20.2 to -17.5)
Slovenia	32 023 (28 280 to 36 139)	1 680 (1 456 to 1 933)	-26.5 (-34.4 to -20.0)	142 839 (132 582 to 155 753)	4 893 (4 517 to 5 339)	-23.4 (-25.0 to -21.9)
**Eastern Europe**	**2 535 669 (2 164 640 to 2 934 117)**	**1 193 (1 022 to 1 405)**	**-12.5 (-16.8 to -8.4)**	**9 719 337 (8 940 040 to 10 647 866)**	**3 414 (3 137 to 3 738)**	**-12.9 (-14.6 to -11.0)**
Belarus	100 142 (88 843 to 113 176)	1 052 (918 to 1 202)	-16.3 (-25.5 to -5.8)	386 591 (358 358 to 421 610)	2 988 (2 769 to 3 253)	-17.0 (-18.6 to -15.6)
Estonia	13 599 (11 853 to 15 520)	1 074 (924 to 1 254)	-29.5 (-37.5 to -20.9)	57 288 (52 956 to 62 633)	3 103 (2 865 to 3 392)	-28.2 (-29.4 to -26.9)
Latvia	21 609 (18 970 to 24 426)	1 143 (992 to 1 311)	-31.5 (-38.7 to -24.6)	90 654 (83 894 to 99 127)	3 237 (2 986 to 3 542)	-31.0 (-32.1 to -30.0)
Lithuania	34 454 (30 264 to 38 650)	1 212 (1 051 to 1 397)	-22.6 (-31.0 to -13.1)	140 291 (129 970 to 153 982)	3 415 (3 159 to 3 748)	-22.5 (-23.8 to -20.9)
Moldova	33 474 (29 335 to 37 731)	884 (771 to 1 012)	-30.4 (-37.0 to -22.0)	123 859 (114 426 to 134 541)	2 543 (2 353 to 2 758)	-31.5 (-32.5 to -30.4)
Russian Federation	1 815 796 (1 542 761 to 2 111 685)	1 218 (1 036 to 1 434)	-14.2 (-18.3 to -10.1)	6 856 229 (6 309 419 to 7 521 276)	3 493 (3 210 to 3 828)	-14.5 (-16.5 to -12.3)
Ukraine	516 594 (438 209 to 602 891)	1 177 (997 to 1 397)	-1.9 (-7.8 to 3.9)	2 064 424 (1 892 239 to 2 260 648)	3 354 (3 078 to 3 670)	-3.2 (-4.6 to -1.7)
**High-income**	**8 841 525 (7 810 602 to 10 005 336)**	**840 (737 to 975)**	**-19.9 (-24.0 to -15.6)**	**36 168 567 (33 543 888 to 39 185 495)**	**2 462 (2 286 to 2 672)**	**-19.2 (-20.3 to -18.2)**
**Australasia**	**365 586 (326 635 to 409 673)**	**1 304 (1 157 to 1 480)**	**-20.3 (-25.6 to -14.6)**	**1 413 829 (1 309 726 to 1 533 369)**	**3 825 (3 541 to 4 145)**	**-19.3 (-20.9 to -18.1)**
Australia	285 446 (251 293 to 324 837)	1 214 (1 058 to 1 401)	-23.2 (-30.3 to -15.7)	1 111 015 (1 028 964 to 1 203 990)	3 579 (3 315 to 3 882)	-21.8 (-23.6 to -20.3)
New Zealand	80 140 (74 271 to 86 535)	1 798 (1 656 to 1 960)	-5.9 (-11.9 to 0.5)	302 814 (279 258 to 328 615)	5 166 (4 760 to 5 603)	-7.5 (-9.1 to -6.2)
**High-income Asia-Pacific**	**1 335 884 (1 176 156 to 1 502 607)**	**686 (594 to 806)**	**-28.3 (-33.2 to -23.5)**	**5 792 577 (5 400 516 to 6 282 687)**	**2 011 (1 868 to 2 178)**	**-24.0 (-25.5 to -22.5)**
Brunei	4 670 (4 053 to 5 289)	1 054 (927 to 1 181)	-28.3 (-34.5 to -21.5)	12 145 (11 302 to 13 097)	2 898 (2 703 to 3 125)	-29.1 (-30.2 to -28.2)
Japan	859 980 (745 042 to 973 415)	664 (566 to 791)	-22.7 (-27.7 to -17.5)	4 113 525 (3 829 491 to 4 464 379)	2 002 (1 859 to 2 167)	-18.0 (-20.0 to -15.9)
South Korea	433 491 (387 384 to 487 353)	757 (667 to 871)	-40.6 (-46.6 to -33.3)	1 529 429 (1 429 084 to 1 655 718)	2 086 (1 950 to 2 257)	-40.7 (-41.8 to -39.6)
Singapore	37 742 (32 788 to 43 547)	681 (587 to 807)	-7.3 (-16.1 to 1.0)	137 479 (127 554 to 148 350)	2 026 (1 879 to 2 185)	-2.0 (-4.7 to 0.9)
**High-income North America**	**3 399 537 (2 941 015 to 3 919 587)**	**950 (814 to 1 115)**	**-21.7 (-25.8 to -17.6)**	**13 171 952 (12 026 067 to 14 553 077)**	**2 806 (2 558 to 3 106)**	**-22.3 (-23.7 to -20.5)**
Canada	290 062 (257 524 to 329 723)	865 (754 to 1 013)	-24.2 (-32.0 to -15.4)	1 217 767 (1 121 708 to 1 330 128)	2 533 (2 333 to 2 772)	-22.8 (-24.5 to -21.4)
Greenland	272 (236 to 316)	495 (425 to 587)	-23.9 (-30.5 to -16.8)	956 (882 to 1 042)	1 466 (1 350 to 1 598)	-25.1 (-27.2 to -23.0)
USA	3 109 143 (2 677 861 to 3 582 413)	960 (820 to 1 134)	-21.3 (-25.8 to -16.8)	11 952 998 (10 896 374 to 13 225 377)	2 840 (2 584 to 3 146)	-22.1 (-23.7 to -20.1)
**Southern Latin America**	**460 533 (410 553 to 514 073)**	**690 (613 to 778)**	**40.0 (28.9 to 51.8)**	**1 458 078 (1 358 268 to 1 574 354)**	**1 965 (1 827 to 2 122)**	**33.6 (31.1 to 36.0)**
Argentina	326 577 (287 719 to 369 475)	727 (639 to 829)	46.7 (33.0 to 62.8)	1 021 089 (948 414 to 1 105 770)	2 096 (1 945 to 2 268)	41.1 (38.4 to 43.9)
Chile	106 550 (97 081 to 117 415)	579 (525 to 641)	19.8 (10.6 to 28.9)	342 469 (319 790 to 367 834)	1 610 (1 505 to 1 727)	13.6 (11.3 to 15.7)

Location	Incidence (95% UI)			Prevalence (95% UI)		
	2017 counts	2017 age-standardised rates per 100,000	Percentage change in age-standardised rates between 1990 and 2017	2017 counts	2017 age-standardised rates per 100,000	Percentage change in age-standardised rates between 1990 and 2017
Uruguay	27 386 (24 186 to 30 631)	790 (693 to 896)	50.7 (37.5 to 65.9)	94 455 (87 817 to 102 133)	2 241 (2 085 to 2 416)	44.9 (41.2 to 48.5)
**Western Europe**	**3 279 986 (2 912 763 to 3 707 329)**	**803 (699 to 939)**	**-21.1 (-27.3 to -15.0)**	**14 332 131 (13 309 926 to 15 526 258)**	**2 378 (2 203 to 2 573)**	**-19.0 (-20.7 to -17.6)**
Andorra	669 (589 to 762)	893 (772 to 1 045)	-1.6 (-9.4 to 6.4)	2 906 (2 697 to 3 161)	2 654 (2 454 to 2 880)	0.0 (-1.4 to 1.3)
Austria	68 018 (59 892 to 77 750)	836 (718 to 985)	-23.9 (-32.2 to -14.8)	302 178 (280 561 to 328 511)	2 483 (2 296 to 2 699)	-20.9 (-23.2 to -19.0)
Belgium	88 023 (78 734 to 98 989)	794 (694 to 915)	-30.3 (-36.9 to -22.9)	361 442 (335 975 to 391 875)	2 311 (2 142 to 2 500)	-29.2 (-31.3 to -27.4)
Cyprus	13 212 (11 741 to 14 867)	1 050 (921 to 1 221)	-22.9 (-31.1 to -14.4)	49 232 (45 667 to 53 358)	3 049 (2 826 to 3 303)	-22.6 (-24.0 to -21.4)
Denmark	43 292 (38 055 to 49 758)	808 (696 to 960)	-18.9 (-27.5 to -10.3)	184 313 (170 906 to 199 986)	2 402 (2 222 to 2 604)	-16.2 (-17.7 to -14.7)
Finland	44 338 (39 083 to 50 521)	869 (751 to 1 016)	-7.0 (-16.1 to 2.1)	197 040 (183 171 to 214 406)	2 570 (2 373 to 2 793)	-3.7 (-4.9 to -2.4)
France	540 656 (477 251 to 615 954)	882 (768 to 1 032)	-25.4 (-32.8 to -17.8)	2 309 036 (2 148 566 to 2 506 643)	2 617 (2 428 to 2 839)	-23.3 (-25.9 to -21.1)
Germany	623 851 (548 376 to 711 948)	820 (702 to 968)	-18.4 (-26.7 to -9.5)	2 902 944 (2 694 020 to 3 149 456)	2 429 (2 247 to 2 632)	-16.3 (-18.3 to -14.6)
Greece	103 199 (91 742 to 115 242)	1 044 (916 to 1 210)	-12.3 (-20.1 to -3.6)	468 839 (436 740 to 508 641)	3 051 (2 833 to 3 299)	-10.8 (-12.6 to -9.3)
Iceland	2 616 (2 286 to 3 015)	805 (689 to 950)	-9.9 (-18.0 to -0.9)	9 953 (9 224 to 10 774)	2 397 (2 219 to 2 591)	-7.3 (-9.5 to -5.6)
Ireland	36 379 (31 497 to 42 515)	790 (675 to 941)	-12.0 (-21.0 to -0.9)	138 737 (128 464 to 150 663)	2 354 (2 173 to 2 551)	-8.7 (-10.4 to -7.0)
Israel	69 443 (60 792 to 80 687)	783 (682 to 915)	-6.1 (-15.1 to 3.1)	219 581 (204 030 to 237 948)	2 274 (2 110 to 2 463)	-5.3 (-7.0 to -3.8)
Italy	493 255 (446 890 to 543 000)	830 (732 to 943)	-24.4 (-30.6 to -17.7)	2 203 371 (2 054 997 to 2 382 958)	2 403 (2 237 to 2 594)	-22.7 (-24.0 to -21.3)
Luxembourg	5 586 (4 890 to 6 364)	979 (847 to 1 143)	-18.7 (-28.5 to -9.4)	22 208 (20 647 to 24 128)	2 883 (2 673 to 3 129)	-16.4 (-19.1 to -14.2)
Malta	2 835 (2 496 to 3 226)	719 (621 to 843)	10.6 (1.8 to 19.8)	12 939 (12 031 to 13 990)	2 147 (1 991 to 2 319)	13.9 (12.3 to 15.6)
Netherlands	112 157 (104 349 to 121 958)	695 (638 to 769)	-17.3 (-24.9 to -8.7)	470 159 (438 032 to 506 556)	2 035 (1 890 to 2 190)	-15.7 (-18.1 to -13.3)
Norway	37 682 (32 398 to 43 940)	742 (629 to 889)	-4.2 (-9.2 to 1.4)	155 360 (143 705 to 169 408)	2 263 (2 089 to 2 462)	-1.7 (-3.7 to 0.6)
Portugal	81 004 (72 071 to 91 485)	768 (667 to 894)	-38.8 (-45.5 to -31.9)	354 662 (329 629 to 384 046)	2 238 (2 079 to 2 419)	-38.5 (-40.9 to -36.6)
Spain	368 356 (324 617 to 419 664)	855 (738 to 1 010)	-26.0 (-33.6 to -17.1)	1 661 725 (1 541 448 to 1 802 218)	2 540 (2 352 to 2 750)	-23.4 (-25.8 to -21.4)
Sweden	65 182 (56 194 to 75 588)	690 (586 to 832)	-13.0 (-18.6 to -7.4)	286 076 (264 406 to 310 997)	2 117 (1 951 to 2 297)	-10.7 (-12.5 to -9.0)
Switzerland	67 985 (61 335 to 75 217)	824 (732 to 948)	-20.7 (-27.2 to -13.8)	289 283 (269 723 to 312 082)	2 409 (2 239 to 2 594)	-19.6 (-21.0 to -18.3)
United Kingdom	408 849 (351 584 to 476 468)	653 (556 to 784)	-10.7 (-16.1 to -5.2)	1 715 296 (1 587 660 to 1 865 592)	1 955 (1 807 to 2 124)	-8.5 (-10.1 to -7.0)
**Latin America and Caribbean**	**2 420 944 (2 125 463 to 2 732 730)**	**406 (356 to 457)**	**31.1 (25.3 to 36.7)**	**7 020 885 (6 520 321 to 7 519 492)**	**1 178 (1 096 to 1 262)**	**29.1 (25.6 to 32.7)**
**Andean Latin America**	**194 102 (170 730 to 216 867)**	**321 (284 to 358)**	**18.4 (9.7 to 27.4)**	**518 087 (485 803 to 552 945)**	**903 (847 to 963)**	**11.3 (9.4 to 13.0)**
Bolivia	31 319 (27 214 to 35 937)	289 (252 to 329)	-3.9 (-11.8 to 4.4)	79 987 (74 978 to 85 493)	821 (771 to 877)	-9.4 (-11.5 to -7.2)
Ecuador	66 229 (57 960 to 74 230)	400 (352 to 446)	27.8 (15.6 to 41.3)	175 503 (164 222 to 187 216)	1 117 (1 045 to 1 192)	21.3 (18.7 to 23.9)
Peru	96 554 (84 405 to 108 468)	293 (256 to 330)	20.1 (9.7 to 31.7)	262 597 (246 143 to 280 722)	823 (771 to 880)	11.8 (9.8 to 13.6)
**Caribbean**	**151 296 (132 325 to 170 382)**	**319 (278 to 359)**	**17.2 (8.5 to 26.0)**	**450 765 (419 736 to 485 368)**	**906 (844 to 975)**	**11.2 (10.2 to 12.5)**
Antigua and Barbuda	231 (201 to 262)	254 (222 to 293)	18.0 (7.9 to 28.7)	741 (692 to 796)	731 (682 to 785)	17.6 (15.3 to 20.0)
The Bahamas	1 213 (1 060 to 1 370)	313 (274 to 353)	2.4 (-6.4 to 12.1)	3 576 (3 328 to 3 853)	885 (825 to 951)	2.1 (1.0 to 3.2)
Barbados	860 (755 to 961)	284 (245 to 322)	21.1 (10.2 to 32.1)	3 149 (2 948 to 3 402)	805 (752 to 867)	21.1 (18.7 to 23.4)
Belize	1 249 (1 088 to 1 419)	319 (280 to 358)	22.6 (12.1 to 34.9)	3 075 (2 858 to 3 310)	938 (872 to 1 013)	20.3 (18.5 to 22.3)
Bermuda	272 (241 to 306)	405 (354 to 467)	-0.3 (-9.9 to 9.3)	1 090 (1 012 to 1 175)	1 149 (1 067 to 1 235)	2.1 (-1.4 to 6.1)
Cuba	29 644 (26 125 to 33 042)	248 (216 to 283)	-16.2 (-25.0 to -7.8)	107 278 (100 090 to 115 621)	693 (645 to 745)	-19.4 (-21.2 to -17.8)
Dominica	206 (182 to 231)	288 (251 to 323)	32.3 (19.6 to 45.9)	679 (633 to 729)	836 (778 to 899)	34.7 (32.9 to 36.6)
Dominican Republic	51 695 (45 152 to 58 133)	488 (428 to 545)	83.7 (65.1 to 103.9)	137 379 (127 517 to 148 529)	1 373 (1 273 to 1 486)	69.6 (66.8 to 72.6)
Grenada	262 (228 to 295)	228 (196 to 259)	12.1 (1.4 to 24.6)	871 (817 to 937)	670 (628 to 721)	13.0 (10.6 to 15.3)
Guyana	1 825 (1 579 to 2 081)	245 (214 to 278)	40.2 (24.0 to 56.0)	5 014 (4 653 to 5 397)	720 (669 to 774)	36.8 (34.4 to 38.9)
Haiti	28 760 (24 235 to 33 719)	255 (218 to 293)	-3.8 (-11.1 to 4.6)	71 792 (66 846 to 76 834)	779 (727 to 837)	-8.0 (-10.1 to -6.2)
Jamaica	7 785 (6 712 to 8 941)	267 (232 to 307)	93.2 (78.4 to 112.5)	22 749 (21 192 to 24 492)	773 (720 to 832)	87.1 (83.2 to 91.4)
Puerto Rico	14 014 (12 413 to 15 742)	363 (316 to 418)	8.0 (-1.7 to 18.0)	51 871 (48 483 to 56 062)	1 016 (948 to 1 094)	8.0 (6.0 to 10.4)
Saint Lucia	528 (463 to 596)	286 (250 to 325)	7.4 (-2.6 to 18.3)	1 681 (1 569 to 1 814)	825 (770 to 889)	6.3 (5.1 to 7.5)
Saint Vincent and the Grenadines	270 (235 to 305)	231 (199 to 264)	41.3 (26.9 to 56.7)	883 (823 to 949)	686 (640 to 737)	43.3 (40.7 to 46.1)
Suriname	1 922 (1 682 to 2 177)	331 (290 to 376)	19.8 (7.9 to 32.5)	5 727 (5 328 to 6 175)	955 (889 to 1 031)	16.4 (15.2 to 17.8)
Trinidad and Tobago	4 744 (4 187 to 5 332)	328 (288 to 371)	28.8 (16.9 to 43.3)	15 653 (14 623 to 16 934)	935 (874 to 1 008)	26.8 (25.0 to 28.9)
Virgin Islands	376 (334 to 419)	337 (294 to 381)	21.1 (11.7 to 31.2)	1 347 (1 258 to 1 456)	925 (866 to 997)	16.3 (14.5 to 18.1)
**Central Latin America**	**983 527 (855 913 to 1 116 551)**	**384 (334 to 436)**	**17.3 (11.7 to 22.9)**	**2 707 080 (2 524 017 to 2 897 252)**	**1 092 (1 019 to 1 169)**	**14.4 (12.7 to 16.4)**
Colombia	146 782 (128 483 to 165 597)	284 (247 to 320)	-8.6 (-16.2 to -1.0)	416 002 (390 216 to 446 029)	780 (732 to 836)	-11.5 (-14.0 to -9.4)
Costa Rica	17 097 (15 088 to 19 127)	354 (312 to 396)	16.9 (4.9 to 28.1)	48 087 (45 092 to 51 268)	963 (903 to 1 026)	14.4 (13.1 to 15.7)
El Salvador	17 591 (15 611 to 19 763)	295 (263 to 331)	7.0 (-4.0 to 18.8)	45 555 (42 602 to 48 684)	781 (730 to 836)	-0.7 (-2.7 to 1.2)
Guatemala	38 593 (33 078 to 44 513)	251 (218 to 282)	25.2 (15.2 to 36.9)	90 799 (84 936 to 97 345)	700 (656 to 749)	18.2 (16.5 to 19.8)
Honduras	23 900 (20 626 to 27 233)	284 (247 to 321)	35.4 (24.0 to 45.9)	57 310 (53 678 to 61 415)	804 (752 to 861)	28.4 (25.7 to 30.9)
Mexico	587 603 (507 029 to 675 684)	461 (399 to 528)	35.9 (29.7 to 42.6)	1 639 241 (1 522 198 to 1 763 199)	1 325 (1 232 to 1 426)	32.1 (29.5 to 35.3)
Nicaragua	12 883 (11 240 to 14 728)	212 (186 to 240)	-13.4 (-21.5 to -4.2)	31 598 (29 629 to 33 780)	592 (556 to 633)	-17.8 (-19.3 to -16.2)

Location	Incidence (95% UI)			Prevalence (95% UI)		
	2017 counts	2017 age-standardised rates per 100,000	Percentage change in age-standardised rates between 1990 and 2017	2017 counts	2017 age-standardised rates per 100,000	Percentage change in age-standardised rates between 1990 and 2017
Panama	12 479 (11 093 to 13 984)	315 (279 to 353)	1.3 (-7.1 to 10.9)	34 821 (32 453 to 37 293)	873 (814 to 935)	-1.5 (-2.8 to -0.4)
Venezuela	126 599 (109 908 to 143 915)	406 (354 to 460)	-3.2 (-12.2 to 7.1)	343 668 (321 169 to 368 190)	1 130 (1 057 to 1 210)	-5.6 (-7.3 to -4.0)
**Tropical Latin America**	**1 092 019 (946 059 to 1 248 763)**	**475 (413 to 542)**	**50.5 (42.1 to 58.5)**	**3 344 953 (3 033 427 to 3 671 892)**	**1 393 (1 264 to 1 526)**	**50.2 (42.9 to 57.8)**
Brazil	1 063 263 (921 016 to 1 217 395)	477 (414 to 546)	50.2 (41.7 to 58.1)	3 271 170 (2 965 341 to 3 591 705)	1 398 (1 268 to 1 533)	50.0 (42.5 to 57.6)
Paraguay	28 756 (24 785 to 33 188)	410 (356 to 468)	70.2 (49.7 to 91.1)	73 782 (67 955 to 79 992)	1 195 (1 101 to 1 294)	63.5 (60.3 to 67.2)
**North Africa and Middle East**	**3 647 741 (3 169 298 to 4 151 722)**	**603 (528 to 681)**	**-11.8 (-16.3 to -7.5)**	**9 314 458 (8 670 467 to 10 054 284)**	**1 746 (1 623 to 1 883)**	**-15.5 (-16.4 to -14.5)**
**North Africa and Middle East**	**3 647 741 (3 169 298 to 4 151 722)**	**603 (528 to 681)**	**-11.8 (-16.3 to -7.5)**	**9 314 458 (8 670 467 to 10 054 284)**	**1 746 (1 623 to 1 883)**	**-15.5 (-16.4 to -14.5)**
Afghanistan	120 735 (101 019 to 144 842)	410 (350 to 474)	-12.6 (-19.6 to -5.1)	243 780 (226 944 to 261 891)	1 305 (1 214 to 1 403)	-16.1 (-17.3 to -14.9)
Algeria	266 520 (229 931 to 304 344)	650 (563 to 736)	-23.1 (-28.3 to -17.3)	731 389 (679 604 to 787 831)	1 907 (1 774 to 2 055)	-25.9 (-26.9 to -24.9)
Bahrain	8 512 (7 383 to 9 678)	555 (490 to 622)	-31.9 (-38.8 to -25.2)	26 295 (24 448 to 28 394)	1 629 (1 516 to 1 751)	-33.1 (-34.8 to -31.5)
Egypt	632 244 (545 004 to 721 060)	675 (585 to 761)	-7.4 (-15.1 to -0.0)	1 527 082 (1 415 912 to 1 650 722)	1 967 (1 822 to 2 130)	-12.5 (-14.0 to -11.0)
Iran	578 111 (488 467 to 680 061)	673 (575 to 782)	-23.5 (-27.0 to -19.9)	1 610 188 (1 496 935 to 1 742 511)	1 934 (1 799 to 2 087)	-27.6 (-28.9 to -26.5)
Iraq	144 667 (123 156 to 167 646)	340 (293 to 385)	-40.4 (-45.2 to -34.5)	342 614 (320 955 to 365 974)	1 052 (984 to 1 126)	-38.5 (-40.4 to -37.1)
Jordan	45 306 (39 054 to 51 777)	427 (372 to 482)	-30.4 (-38.0 to -23.3)	105 098 (98 289 to 112 582)	1 230 (1 151 to 1 319)	-32.9 (-34.3 to -31.8)
Kuwait	32 117 (27 991 to 36 359)	725 (642 to 810)	-27.6 (-35.1 to -18.9)	88 118 (82 196 to 95 164)	2 105 (1 963 to 2 263)	-28.1 (-29.2 to -27.0)
Lebanon	48 469 (41 667 to 57 351)	550 (474 to 643)	11.7 (1.9 to 22.1)	119 686 (111 474 to 128 769)	1 583 (1 474 to 1 703)	8.8 (7.1 to 10.7)
Libya	55 945 (47 720 to 64 764)	785 (678 to 894)	-14.2 (-20.8 to -6.7)	139 417 (128 767 to 151 549)	2 238 (2 069 to 2 432)	-17.6 (-18.7 to -16.5)
Morocco	208 224 (178 746 to 237 422)	572 (493 to 648)	-10.0 (-17.3 to -2.6)	594 570 (553 473 to 642 870)	1 694 (1 577 to 1 831)	-14.0 (-15.2 to -12.8)
Palestine	15 154 (12 770 to 17 873)	318 (272 to 367)	-10.2 (-17.7 to -2.5)	34 146 (31 879 to 36 544)	958 (894 to 1 026)	-9.6 (-10.8 to -8.3)
Oman	56 754 (48 500 to 65 770)	1 129 (994 to 1 269)	-29.0 (-35.1 to -23.3)	125 723 (116 140 to 137 359)	3 146 (2 918 to 3 413)	-31.2 (-32.4 to -30.0)
Qatar	30 692 (26 304 to 35 541)	966 (850 to 1 083)	-16.7 (-25.1 to -8.5)	71 734 (66 519 to 77 939)	2 728 (2 535 to 2 946)	-19.7 (-20.8 to -18.4)
Saudi Arabia	400 922 (342 397 to 462 774)	1 045 (912 to 1 183)	-11.3 (-21.3 to -1.1)	890 743 (818 669 to 979 730)	2 920 (2 686 to 3 198)	-15.6 (-17.1 to -14.1)
Sudan	203 304 (172 244 to 236 657)	553 (479 to 627)	-17.4 (-23.7 to -11.7)	454 834 (422 501 to 489 485)	1 717 (1 594 to 1 852)	-20.0 (-21.2 to -18.8)
Syria	68 376 (58 019 to 79 057)	389 (333 to 446)	-10.1 (-19.0 to -0.4)	172 789 (161 593 to 185 391)	1 126 (1 054 to 1 208)	-13.2 (-14.5 to -12.0)
Tunisia	65 575 (56 466 to 75 506)	553 (477 to 638)	0.6 (-7.2 to 9.1)	202 123 (187 136 to 218 662)	1 608 (1 490 to 1 738)	-3.8 (-5.3 to -2.1)
Turkey	367 899 (321 637 to 416 795)	443 (386 to 504)	17.2 (8.3 to 26.5)	1 129 132 (1 050 999 to 1 213 776)	1 275 (1 188 to 1 369)	9.1 (7.1 to 10.9)
United Arab Emirates	123 480 (103 823 to 145 886)	1 132 (1 002 to 1 275)	-15.7 (-22.9 to -7.7)	327 381 (303 368 to 355 742)	3 182 (2 956 to 3 443)	-17.8 (-18.9 to -16.7)
Yemen	171 330 (147 283 to 198 218)	624 (546 to 709)	-21.1 (-26.5 to -14.6)	368 918 (344 033 to 396 299)	1 890 (1 761 to 2 036)	-23.6 (-24.9 to -22.5)
**South Asia**	**8 457 957 (7 169 469 to 10 031 040)**	**462 (393 to 545)**	**49.0 (42.3 to 55.6)**	**23 048 088 (21 253 672 to 25 062 773)**	**1 437 (1 327 to 1 567)**	**43.1 (40.6 to 46.2)**
**South Asia**	**8 457 957 (7 169 469 to 10 031 040)**	**462 (393 to 545)**	**49.0 (42.3 to 55.6)**	**23 048 088 (21 253 672 to 25 062 773)**	**1 437 (1 327 to 1 567)**	**43.1 (40.6 to 46.2)**
Bangladesh	488 246 (416 666 to 570 648)	298 (255 to 346)	68.2 (54.9 to 85.0)	1 371 652 (1 264 865 to 1 487 123)	950 (878 to 1 032)	60.3 (57.1 to 63.7)
Bhutan	3 851 (3 235 to 4 536)	367 (312 to 429)	0.9 (-8.5 to 11.1)	10 086 (9 280 to 10 959)	1 168 (1 077 to 1 267)	-2.8 (-5.1 to -0.4)
India	6 596 837 (5 538 899 to 7 879 123)	463 (390 to 549)	45.0 (37.4 to 53.2)	18 375 144 (16 927 415 to 20 047 566)	1 445 (1 332 to 1 577)	40.3 (37.6 to 43.3)
Nepal	132 834 (113 152 to 156 543)	437 (375 to 508)	36.0 (27.7 to 45.0)	353 306 (325 678 to 383 730)	1 382 (1 276 to 1 504)	32.0 (29.5 to 34.8)
Pakistan	1 236 189 (1 057 252 to 1 460 214)	568 (490 to 663)	61.3 (50.0 to 73.3)	2 937 900 (2 695 517 to 3 198 542)	1 815 (1 670 to 1 981)	58.5 (55.5 to 62.0)
**Southeast Asia, East Asia, and Oceania**	**20 809 598 (18 003 124 to 23 852 398)**	**882 (772 to 999)**	**92.9 (85.1 to 100.8)**	**69 014 270 (63 694 029 to 74 704 932)**	**2 627 (2 431 to 2 840)**	**81.3 (77.7 to 85.1)**
**East Asia**	**15 572 911 (13 389 614 to 17 938 779)**	**939 (811 to 1 072)**	**111.2 (101.4 to 120.8)**	**53 552 607 (49 383 606 to 58 086 220)**	**2 741 (2 531 to 2 972)**	**94.9 (90.7 to 99.4)**
China	14 840 576 (12 727 729 to 17 118 937)	940 (810 to 1 075)	113.9 (103.7 to 123.6)	50 992 074 (47 007 604 to 55 308 501)	2 744 (2 534 to 2 975)	97.0 (92.6 to 101.8)
North Korea	229 875 (198 667 to 265 267)	824 (717 to 950)	110.0 (95.2 to 126.4)	765 861 (709 715 to 830 351)	2 462 (2 285 to 2 663)	107.4 (103.7 to 111.4)
Taiwan (Province of China)	251 589 (222 814 to 280 305)	990 (876 to 1 115)	23.0 (10.4 to 37.8)	931 969 (862 516 to 1 007 055)	2 820 (2 614 to 3 042)	19.0 (14.4 to 23.7)
**Oceania**	**90 566 (77 855 to 104 811)**	**744 (647 to 853)**	**68.2 (56.5 to 81.5)**	**213 271 (198 008 to 231 651)**	**2 271 (2 110 to 2 472)**	**66.5 (64.2 to 69.1)**
American Samoa	344 (298 to 393)	632 (547 to 722)	59.6 (46.0 to 74.2)	945 (877 to 1 018)	1 887 (1 755 to 2 035)	56.0 (53.6 to 58.9)
Federated States of Micronesia	730 (623 to 848)	708 (606 to 824)	83.6 (69.1 to 99.8)	1 919 (1 777 to 2 080)	2 172 (2 007 to 2 356)	78.7 (75.5 to 81.9)
Fiji	5 541 (4 815 to 6 347)	602 (525 to 688)	83.8 (64.7 to 103.3)	15 887 (14 786 to 17 120)	1 836 (1 709 to 1 979)	81.0 (77.8 to 84.9)
Guam	1 569 (1 370 to 1 782)	933 (816 to 1 063)	66.6 (51.7 to 81.6)	4 826 (4 470 to 5 219)	2 700 (2 502 to 2 913)	65.5 (61.7 to 69.7)
Kiribati	543 (458 to 635)	465 (397 to 539)	103.5 (86.3 to 123.1)	1 377 (1 280 to 1 488)	1 471 (1 366 to 1 591)	101.2 (96.6 to 107.4)
Marshall Islands	425 (364 to 491)	755 (650 to 866)	93.3 (77.2 to 112.2)	1 048 (971 to 1 137)	2 299 (2 132 to 2 497)	89.9 (86.6 to 93.9)
Northern Mariana Islands	359 (314 to 409)	773 (674 to 881)	26.0 (15.9 to 37.0)	1 206 (1 120 to 1 306)	2 222 (2 071 to 2 397)	23.4 (21.5 to 25.6)
Papua New Guinea	68 220 (58 294 to 79 140)	769 (665 to 883)	60.9 (48.2 to 75.8)	154 930 (143 456 to 168 865)	2 368 (2 192 to 2 586)	59.6 (57.1 to 62.1)
Samoa	1 244 (1 069 to 1 454)	677 (585 to 784)	85.8 (70.9 to 103.7)	3 277 (3 044 to 3 534)	2 072 (1 923 to 2 235)	81.9 (79.1 to 85.2)
Solomon Islands	3 966 (3 381 to 4 637)	662 (571 to 766)	78.2 (64.6 to 92.0)	9 450 (8 773 to 10 244)	2 081 (1 934 to 2 257)	75.7 (72.9 to 79.6)
Tonga	583 (505 to 665)	585 (508 to 664)	86.6 (71.9 to 104.2)	1 571 (1 463 to 1 694)	1 772 (1 652 to 1 910)	81.0 (78.1 to 84.9)
Vanuatu	2 052 (1 747 to 2 396)	762 (652 to 879)	88.9 (76.8 to 102.6)	5 082 (4 729 to 5 492)	2 394 (2 220 to 2 596)	86.9 (84.4 to 89.8)
**Southeast Asia**	**5 146 121 (4 493 905 to 5 855 603)**	**748 (657 to 851)**	**50.6 (42.9 to 58.5)**	**15 248 393 (14 122 438 to 16 448 412)**	**2 289 (2 123 to 2 466)**	**44.6 (41.7 to 47.7)**
Cambodia	116 215 (99 543 to 134 834)	719 (620 to 825)	63.4 (51.5 to 76.5)	298 196 (276 989 to 320 552)	2 149 (1 997 to 2 311)	55.8 (52.4 to 59.4)
Indonesia	1 882 125 (1 592 917 to 2 213 048)	703 (600 to 822)	38.7 (30.4 to 47.5)	5 621 101 (5 189 045 to 6 077 804)	2 200 (2 033 to 2 375)	32.3 (28.3 to 36.7)

Location	Incidence (95% UI)			Prevalence (95% UI)		
	2017 counts	2017 age-standardised rates per 100,000	Percentage change in age-standardised rates between 1990 and 2017	2017 counts	2017 age-standardised rates per 100,000	Percentage change in age-standardised rates between 1990 and 2017
Laos	55 544 (47 543 to 63 808)	788 (680 to 893)	50.4 (39.3 to 63.8)	139 068 (129 223 to 149 092)	2 449 (2 276 to 2 628)	45.2 (42.0 to 48.2)
Malaysia	367 736 (321 285 to 416 265)	1 134 (996 to 1 273)	68.5 (51.8 to 85.9)	967 238 (896 405 to 1 040 041)	3 254 (3 012 to 3 504)	59.2 (56.6 to 61.8)
Maldives	3 472 (2 894 to 4 173)	670 (572 to 786)	42.1 (31.7 to 52.8)	8 382 (7 804 to 9 042)	1 946 (1 812 to 2 097)	30.5 (27.8 to 33.8)
Mauritius	9 898 (8 636 to 11 206)	743 (646 to 857)	86.8 (70.4 to 103.2)	34 165 (31 704 to 36 865)	2 159 (2 005 to 2 327)	84.7 (80.1 to 89.7)
Myanmar	355 327 (307 093 to 402 527)	645 (559 to 728)	52.3 (39.7 to 65.8)	1 006 447 (935 340 to 1 080 868)	1 960 (1 823 to 2 102)	44.7 (41.3 to 48.1)
Philippines	530 282 (461 890 to 606 945)	513 (449 to 582)	98.2 (79.6 to 120.1)	1 361 830 (1 268 947 to 1 460 207)	1 527 (1 422 to 1 641)	90.7 (87.2 to 95.2)
Sri Lanka	149 394 (131 503 to 169 925)	669 (587 to 769)	129.3 (107.9 to 154.5)	463 477 (430 905 to 498 611)	1 913 (1 782 to 2 055)	121.0 (116.9 to 126.3)
Seychelles	824 (724 to 928)	774 (682 to 868)	104.2 (88.3 to 120.1)	2 536 (2 355 to 2 735)	2 228 (2 073 to 2 399)	95.4 (91.9 to 99.0)
Thailand	816 798 (721 207 to 915 096)	1 084 (956 to 1 212)	36.0 (23.0 to 51.6)	2 886 211 (2 657 406 to 3 128 610)	3 122 (2 876 to 3 381)	29.0 (26.4 to 32.1)
Timor-Leste	6 849 (5 778 to 8 067)	566 (481 to 662)	92.6 (79.2 to 106.9)	16 622 (15 476 to 17 780)	1 759 (1 638 to 1 886)	84.0 (80.9 to 87.9)
Vietnam	844 886 (737 516 to 962 944)	821 (718 to 932)	72.7 (56.9 to 89.7)	2 423 058 (2 246 430 to 2 608 362)	2 376 (2 204 to 2 554)	62.3 (59.7 to 65.6)
**Sub-Saharan Africa**	**5 090 705 (4 385 621 to 5 908 108)**	**562 (493 to 638)**	**-16.8 (-20.3 to -13.6)**	**11 177 501 (10 428 363 to 12 021 346)**	**1 703 (1 582 to 1 834)**	**-18.6 (-19.8 to -17.7)**
**Central sub-Saharan Africa**	**856 081 (738 450 to 985 218)**	**751 (660 to 849)**	**-16.7 (-21.9 to -11.5)**	**1 873 278 (1 737 190 to 2 023 688)**	**2 334 (2 166 to 2 534)**	**-18.4 (-19.7 to -17.3)**
Angola	227 429 (196 264 to 260 998)	887 (775 to 996)	-20.7 (-26.0 to -14.5)	475 233 (439 997 to 515 624)	2 734 (2 535 to 2 973)	-23.7 (-25.5 to -22.2)
Central African Republic	33 517 (28 489 to 39 491)	738 (634 to 854)	-13.6 (-19.6 to -6.4)	78 179 (71 888 to 86 166)	2 352 (2 160 to 2 587)	-12.5 (-13.5 to -11.5)
Congo (Brazzaville)	40 694 (35 363 to 46 934)	845 (742 to 962)	-19.7 (-25.6 to -13.8)	98 194 (91 108 to 106 533)	2 620 (2 431 to 2 845)	-19.8 (-21.1 to -18.5)
DR Congo	526 541 (450 018 to 611 636)	693 (605 to 786)	-16.0 (-22.5 to -10.0)	1 158 233 (1 074 163 to 1 251 269)	2 161 (2 004 to 2 343)	-17.4 (-18.7 to -16.2)
Equatorial Guinea	10 954 (9 381 to 12 812)	867 (756 to 989)	-4.9 (-11.9 to 2.6)	20 760 (19 263 to 22 427)	2 523 (2 337 to 2 738)	-13.7 (-16.1 to -11.6)
Gabon	16 947 (14 727 to 19 342)	1 007 (880 to 1 141)	-22.4 (-27.4 to -16.9)	42 679 (39 609 to 46 196)	3 092 (2 872 to 3 345)	-22.4 (-23.5 to -21.4)
**Eastern sub-Saharan Africa**	**1 886 499 (1 620 923 to 2 208 244)**	**567 (497 to 642)**	**-17.8 (-21.4 to -14.5)**	**4 020 143 (3 736 311 to 4 323 682)**	**1 701 (1 583 to 1 838)**	**-19.3 (-20.4 to -18.4)**
Burundi	63 310 (54 770 to 72 428)	691 (606 to 781)	-28.0 (-32.5 to -22.6)	134 988 (125 354 to 145 823)	2 098 (1 948 to 2 280)	-27.4 (-28.6 to -26.3)
Comoros	4 699 (4 095 to 5 336)	697 (609 to 784)	-28.1 (-33.0 to -23.2)	11 822 (10 993 to 12 791)	2 099 (1 952 to 2 272)	-30.2 (-31.5 to -29.2)
Djibouti	8 028 (6 899 to 9 160)	777 (678 to 879)	-16.6 (-22.0 to -11.0)	19 361 (17 968 to 20 922)	2 328 (2 166 to 2 523)	-17.8 (-18.9 to -16.7)
Eritrea	34 840 (29 933 to 40 052)	670 (586 to 760)	-17.8 (-22.8 to -11.2)	72 707 (67 380 to 78 742)	1 963 (1 816 to 2 134)	-18.9 (-20.1 to -17.7)
Ethiopia	339 143 (280 396 to 413 509)	390 (331 to 458)	-38.7 (-42.0 to -35.7)	727 670 (676 923 to 782 426)	1 204 (1 119 to 1 297)	-39.4 (-40.9 to -38.1)
Kenya	298 792 (252 730 to 354 710)	711 (614 to 819)	1.8 (-2.6 to 6.2)	655 045 (604 853 to 708 350)	2 104 (1 944 to 2 281)	0.0 (-0.8 to 0.8)
Madagascar	145 408 (125 723 to 167 745)	657 (574 to 737)	-23.6 (-29.1 to -17.8)	313 009 (291 101 to 338 099)	1 975 (1 835 to 2 144)	-26.1 (-27.7 to -24.9)
Malawi	64 910 (55 340 to 75 495)	435 (379 to 490)	-21.8 (-27.6 to -15.5)	138 709 (129 065 to 148 847)	1 292 (1 205 to 1 388)	-23.7 (-25.2 to -22.6)
Mozambique	153 527 (131 995 to 177 226)	619 (542 to 698)	2.5 (-6.0 to 11.3)	307 480 (286 096 to 333 054)	1 822 (1 692 to 1 981)	-3.4 (-4.7 to -2.1)
Rwanda	78 549 (68 285 to 91 071)	718 (632 to 814)	-32.0 (-36.6 to -26.9)	173 639 (161 246 to 187 387)	2 119 (1 968 to 2 289)	-33.5 (-35.0 to -32.3)
Somalia	103 607 (87 747 to 122 270)	731 (630 to 837)	-9.7 (-15.5 to -3.1)	220 858 (203 693 to 241 505)	2 246 (2 070 to 2 462)	-9.8 (-10.6 to -8.9)
South Sudan	61 813 (53 075 to 71 879)	744 (654 to 845)	-0.1 (-6.1 to 6.2)	131 650 (121 512 to 143 175)	2 276 (2 106 to 2 483)	0.9 (-0.1 to 1.8)
Tanzania	239 805 (206 054 to 278 628)	516 (453 to 581)	-12.2 (-18.8 to -5.4)	527 488 (491 490 to 565 814)	1 549 (1 448 to 1 661)	-13.1 (-14.2 to -12.2)
Uganda	203 889 (175 213 to 236 519)	640 (563 to 721)	2.0 (-4.2 to 8.6)	406 134 (376 766 to 436 297)	1 903 (1 774 to 2 048)	2.5 (1.1 to 3.7)
Zambia	84 994 (73 106 to 98 497)	577 (507 to 648)	-24.6 (-30.0 to -18.6)	177 060 (164 618 to 190 481)	1 720 (1 604 to 1 854)	-25.4 (-26.6 to -24.3)
**Southern sub-Saharan Africa**	**590 513 (502 359 to 689 187)**	**768 (659 to 888)**	**-25.1 (-27.9 to -21.8)**	**1 515 647 (1 399 072 to 1 650 498)**	**2 264 (2 091 to 2 468)**	**-29.3 (-31.2 to -27.5)**
Botswana	14 551 (12 662 to 16 605)	650 (570 to 737)	8.1 (1.1 to 15.4)	36 839 (34 022 to 40 036)	1 927 (1 780 to 2 096)	6.4 (4.9 to 8.0)
Lesotho	15 658 (13 560 to 18 023)	810 (708 to 923)	30.5 (19.5 to 42.1)	37 615 (34 666 to 41 058)	2 390 (2 206 to 2 614)	26.1 (24.2 to 28.2)
Namibia	17 469 (15 134 to 20 194)	758 (663 to 863)	-11.1 (-17.7 to -3.8)	41 635 (38 458 to 45 396)	2 240 (2 072 to 2 445)	-14.1 (-15.3 to -12.8)
South Africa	456 862 (384 538 to 535 410)	811 (687 to 946)	-32.5 (-35.7 to -28.9)	1 209 288 (1 112 684 to 1 319 123)	2 363 (2 176 to 2 582)	-36.4 (-38.4 to -34.5)
Swaziland	9 465 (8 130 to 11 009)	864 (750 to 986)	6.6 (-1.1 to 14.5)	20 916 (19 275 to 22 850)	2 535 (2 336 to 2 779)	2.4 (1.0 to 4.0)
Zimbabwe	76 508 (65 357 to 89 540)	576 (500 to 662)	14.9 (6.9 to 23.9)	169 354 (157 377 to 182 613)	1 714 (1 589 to 1 855)	17.6 (15.9 to 19.5)
**Western sub-Saharan Africa**	**1 757 612 (1 498 547 to 2 054 475)**	**458 (400 to 519)**	**-7.9 (-12.7 to -3.1)**	**3 768 432 (3 517 353 to 4 025 686)**	**1 371 (1 285 to 1 467)**	**-11.0 (-11.9 to -10.1)**
Benin	67 150 (57 257 to 79 132)	658 (572 to 750)	-11.6 (-17.0 to -6.3)	144 255 (134 385 to 154 874)	2 010 (1 874 to 2 157)	-14.5 (-15.9 to -13.1)
Burkina Faso	53 686 (44 262 to 66 002)	261 (222 to 306)	38.4 (27.5 to 50.9)	117 504 (109 112 to 125 900)	841 (785 to 898)	35.3 (33.3 to 37.4)
Cameroon	114 580 (98 517 to 132 814)	467 (411 to 525)	-24.8 (-30.4 to -19.2)	246 554 (229 864 to 263 621)	1 400 (1 305 to 1 503)	-26.5 (-27.5 to -25.6)
Cape Verde	2 393 (2 085 to 2 768)	438 (385 to 503)	31.8 (23.5 to 40.3)	6 340 (5 940 to 6 759)	1 275 (1 194 to 1 361)	25.8 (24.0 to 27.4)
Chad	57 988 (48 621 to 67 513)	446 (391 to 504)	8.0 (1.0 to 15.9)	117 762 (109 923 to 125 837)	1 387 (1 294 to 1 483)	7.5 (6.5 to 8.9)
Cote d'Ivoire	106 983 (91 902 to 124 287)	473 (414 to 536)	-13.1 (-18.9 to -7.0)	240 537 (224 993 to 256 907)	1 451 (1 355 to 1 552)	-14.1 (-14.9 to -13.2)
The Gambia	8 862 (7 589 to 10 423)	464 (406 to 529)	-8.1 (-13.7 to -2.4)	19 272 (17 996 to 20 614)	1 396 (1 304 to 1 497)	-11.2 (-12.1 to -10.3)
Ghana	162 014 (141 204 to 187 500)	582 (510 to 661)	22.3 (12.9 to 32.1)	370 734 (345 920 to 397 129)	1 702 (1 588 to 1 825)	16.6 (15.4 to 17.9)
Guinea	48 438 (41 744 to 56 133)	467 (411 to 525)	-11.7 (-17.9 to -5.0)	108 046 (101 078 to 115 441)	1 440 (1 345 to 1 542)	-14.5 (-16.3 to -12.9)
Guinea-Bissau	7 435 (6 375 to 8 643)	450 (396 to 510)	-24.1 (-29.0 to -19.0)	15 813 (14 726 to 16 919)	1 375 (1 281 to 1 472)	-26.0 (-27.2 to -24.8)
Liberia	14 598 (12 341 to 17 145)	343 (298 to 389)	-23.8 (-29.3 to -18.1)	32 633 (30 423 to 34 931)	1 053 (982 to 1 126)	-26.7 (-27.9 to -25.5)
Mali	77 152 (65 109 to 90 060)	431 (374 to 490)	-22.6 (-27.2 to -18.0)	161 714 (150 438 to 172 933)	1 323 (1 233 to 1 416)	-25.0 (-26.2 to -23.8)
Mauritania	13 807 (11 870 to 16 273)	385 (336 to 440)	-26.1 (-30.8 to -21.1)	31 461 (29 411 to 33 652)	1 166 (1 092 to 1 251)	-27.3 (-28.3 to -26.5)

Location	Incidence (95% UI)			Prevalence (95% UI)		
	2017 counts	2017 age-standardised rates per 100,000	Percentage change in age-standardised rates between 1990 and 2017	2017 counts	2017 age-standardised rates per 100,000	Percentage change in age-standardised rates between 1990 and 2017
Niger	72 137<br>(60 106 to 85 255)	405<br>(352 to 462)	-19.5<br>(-24.6 to -13.6)	143 522<br>(134 190 to 153 819)	1 243<br>(1 163 to 1 332)	-23.1<br>(-24.4 to -21.8)
Nigeria	830 941<br>(702 111 to 977 830)	457<br>(398 to 523)	-7.1<br>(-13.0 to -1.3)	1 741 697<br>(1 622 897 to 1 862 143)	1 347<br>(1 263 to 1 441)	-11.0<br>(-11.9 to -10.0)
Sao Tome and Principe	906<br>(781 to 1 060)	487<br>(426 to 556)	4.9<br>(-3.0 to 12.8)	2 113<br>(1 973 to 2 260)	1 458<br>(1 360 to 1 560)	-0.5<br>(-2.1 to 1.2)
Senegal	56 480<br>(48 779 to 65 999)	428<br>(375 to 484)	-16.1<br>(-22.0 to -10.2)	129 375<br>(120 848 to 138 165)	1 301<br>(1 217 to 1 393)	-18.9<br>(-20.2 to -17.8)
Sierra Leone	30 804<br>(26 702 to 35 625)	441<br>(387 to 496)	-15.8<br>(-20.9 to -9.9)	68 075<br>(63 508 to 72 926)	1 339<br>(1 248 to 1 436)	-19.0<br>(-20.4 to -17.7)
Togo	31 240<br>(26 760 to 36 278)	461<br>(403 to 523)	-12.9<br>(-18.7 to -6.8)	70 988<br>(66 283 to 75 914)	1 381<br>(1 290 to 1 478)	-16.3<br>(-17.8 to -15.1)
