# Supplementary material for: Morbidity and mortality from road injuries: results from the Global Burden of Disease Study 2017
Source: Inj Prev. 2020 Jan 8;26(Suppl 1):i46–56. doi: 10.1136/injuryprev-2019-043302 (PMC7571357; doi:10.1136/injuryprev-2019-043302)
Supplement: Supplementary data [file injuryprev-2019-043302supp004.pdf]

Table 2: Mortality for 2017 and percentage change of age-standardised rates between 1990 and 2017 by location for road injuries

Location	Mortality (95% UI)		
	2017 counts	2017 age-standardised rates per 100,000	Percentage change in age-standardised rates between 1990 and 2017
**Global**	**1 243 068 (1 191 889 to 1 276 940)**	**15.8 (15.2 to 16.3)**	**-29.0 (-33.6 to -25.0)**
**Low SDI**	**210 016 (197 673 to 222 927)**	**20.6 (19.4 to 21.8)**	**-19.0 (-27.2 to -4.8)**
**Low-middle SDI**	**313 285 (289 059 to 331 347)**	**20.6 (18.9 to 21.8)**	**-21.1 (-29.0 to -14.2)**
**Middle SDI**	**376 334 (357 728 to 389 646)**	**17.0 (16.2 to 17.6)**	**-27.4 (-33.7 to -22.7)**
**High-middle SDI**	**234 727 (223 744 to 243 437)**	**15.0 (14.3 to 15.5)**	**-34.6 (-39.8 to -30.5)**
**High SDI**	**103 359 (101 354 to 105 976)**	**7.5 (7.3 to 7.7)**	**-56.0 (-57.1 to -54.8)**
**Central Europe, Eastern Europe, and Central Asia**	**54 869 (53 678 to 56 296)**	**11.9 (11.6 to 12.2)**	**-45.7 (-47.0 to -43.9)**
**Central Asia**	**10 767 (10 146 to 11 452)**	**11.8 (11.2 to 12.5)**	**-39.7 (-43.2 to -35.8)**
Armenia	242 (229 to 258)	7.0 (6.6 to 7.5)	-58.9 (-61.7 to -55.4)
Azerbaijan	645 (562 to 729)	6.2 (5.4 to 6.9)	-61.4 (-67.0 to -56.1)
Georgia	724 (667 to 773)	17.4 (16.1 to 18.6)	-14.0 (-21.0 to -6.9)
Kazakhstan	2 767 (2 555 to 2 999)	14.9 (13.8 to 16.1)	-35.8 (-40.4 to -30.1)
Kyrgyzstan	884 (827 to 953)	14.6 (13.7 to 15.8)	-44.9 (-49.3 to -39.9)
Mongolia	546 (480 to 626)	16.4 (14.5 to 18.7)	10.8 (-9.2 to 35.7)
Tajikistan	645 (577 to 724)	7.6 (6.8 to 8.5)	-46.8 (-53.4 to -39.3)
Turkmenistan	323 (289 to 365)	6.4 (5.8 to 7.3)	-65.8 (-69.9 to -61.4)
Uzbekistan	3 990 (3 483 to 4 535)	12.8 (11.2 to 14.5)	-30.0 (-39.0 to -20.2)
**Central Europe**	**10 977 (10 629 to 11 375)**	**8.0 (7.7 to 8.2)**	**-56.8 (-58.3 to -55.0)**
Albania	248 (201 to 304)	7.9 (6.4 to 9.7)	-39.5 (-51.3 to -24.9)
Bosnia and Herzegovina	269 (244 to 293)	6.4 (5.8 to 7.0)	4.9 (-11.3 to 19.5)
Bulgaria	719 (665 to 782)	8.7 (8.0 to 9.5)	-41.3 (-46.1 to -36.0)
Croatia	379 (353 to 408)	7.3 (6.8 to 7.9)	-61.0 (-63.9 to -57.7)
Czech Republic	853 (794 to 918)	6.6 (6.1 to 7.1)	-53.1 (-56.8 to -49.1)
Hungary	789 (734 to 864)	6.3 (5.9 to 6.9)	-69.1 (-71.4 to -65.9)
Macedonia	161 (145 to 175)	6.5 (5.8 to 7.1)	-29.3 (-38.4 to -21.4)
Montenegro	57 (51 to 63)	7.7 (7.0 to 8.6)	-29.0 (-38.3 to -18.8)
Poland	3 954 (3 704 to 4 234)	8.7 (8.1 to 9.3)	-60.9 (-63.6 to -57.8)
Romania	2 184 (2 044 to 2 330)	9.4 (8.8 to 10.0)	-51.9 (-55.0 to -48.6)
Serbia	780 (721 to 839)	7.1 (6.5 to 7.6)	-52.3 (-57.4 to -45.5)
Slovakia	442 (407 to 493)	7.0 (6.4 to 7.8)	-65.3 (-68.6 to -61.2)
Slovenia	142 (131 to 157)	5.9 (5.4 to 6.5)	-73.9 (-76.3 to -71.2)

Location	Mortality (95% UI)		
	2017 counts	2017 age-standardised rates per 100,000	Percentage change in age-standardised rates between 1990 and 2017
**Eastern Europe**	**33 125 (32 284 to 34 221)**	**14.3 (13.9 to 14.7)**	**-42.0 (-43.7 to -39.7)**
Belarus	990 (913 to 1 085)	8.8 (8.1 to 9.6)	-60.3 (-63.7 to -55.4)
Estonia	90 (78 to 104)	5.8 (5.0 to 6.7)	-78.1 (-81.2 to -74.5)
Latvia	212 (188 to 237)	9.2 (8.1 to 10.4)	-71.9 (-75.4 to -68.1)
Lithuania	325 (302 to 351)	9.2 (8.5 to 10.1)	-67.2 (-69.8 to -63.9)
Moldova	442 (418 to 470)	10.4 (9.8 to 11.0)	-62.5 (-65.0 to -59.9)
Russian Federation	24 385 (23 735 to 25 842)	15.1 (14.7 to 16.0)	-40.4 (-42.2 to -37.1)
Ukraine	6 681 (5 924 to 7 238)	14.0 (12.2 to 15.2)	-36.7 (-44.8 to -31.0)
**High-income**	**99 663 (97 500 to 102 405)**	**7.8 (7.6 to 8.0)**	**-53.0 (-54.2 to -51.6)**
**Australasia**	**2 023 (1 838 to 2 212)**	**6.3 (5.7 to 6.9)**	**-61.7 (-65.3 to -57.8)**
Australia	1 661 (1 482 to 1 847)	6.1 (5.4 to 6.8)	-60.4 (-64.9 to -55.9)
New Zealand	362 (341 to 385)	7.6 (7.2 to 8.1)	-65.3 (-67.5 to -63.0)
**High-income Asia-Pacific**	**14 588 (13 967 to 15 248)**	**5.1 (4.9 to 5.3)**	**-70.4 (-71.8 to -68.8)**
Brunei	68 (60 to 75)	16.5 (14.8 to 18.0)	-49.0 (-55.3 to -42.4)
Japan	7 681 (7 377 to 8 026)	3.8 (3.7 to 4.0)	-65.2 (-66.6 to -63.7)
South Korea	6 643 (6 143 to 7 170)	9.3 (8.6 to 10.0)	-75.8 (-77.7 to -73.6)
Singapore	197 (181 to 213)	3.1 (2.8 to 3.3)	-64.2 (-67.1 to -61.2)
**High-income North America**	**46 958 (45 107 to 48 711)**	**11.6 (11.1 to 12.1)**	**-35.6 (-38.6 to -32.8)**
Canada	2 741 (2 558 to 2 923)	6.7 (6.2 to 7.2)	-54.9 (-58.2 to -51.6)
Greenland	2 (2 to 3)	4.1 (3.7 to 4.6)	-65.6 (-71.3 to -57.2)
USA	44 214 (42 452 to 45 928)	12.2 (11.6 to 12.6)	-33.8 (-37.1 to -30.9)
**Southern Latin America**	**9 348 (8 564 to 10 232)**	**13.1 (12.0 to 14.3)**	**-8.6 (-16.2 to 0.7)**
Argentina	6 457 (5 751 to 7 270)	13.6 (12.2 to 15.3)	1.0 (-10.4 to 14.5)
Chile	2 281 (1 995 to 2 595)	11.2 (9.8 to 12.7)	-29.9 (-38.2 to -20.0)
Uruguay	609 (537 to 681)	15.0 (13.2 to 16.8)	-6.4 (-18.5 to 5.4)
**Western Europe**	**26 747 (25 935 to 27 579)**	**4.9 (4.7 to 5.0)**	**-68.7 (-69.7 to -67.7)**
Andorra	4 (3 to 4)	4.1 (3.5 to 4.8)	-54.4 (-63.2 to -42.5)
Austria	481 (450 to 515)	4.4 (4.1 to 4.8)	-73.6 (-75.5 to -71.3)
Belgium	1 035 (969 to 1 107)	7.0 (6.6 to 7.5)	-64.8 (-67.2 to -62.0)
Cyprus	152 (137 to 167)	10.2 (9.3 to 11.3)	-60.1 (-67.5 to -53.9)
Denmark	283 (263 to 304)	4.0 (3.7 to 4.3)	-69.8 (-72.2 to -67.2)
Finland	289 (270 to 310)	4.2 (3.9 to 4.5)	-69.3 (-71.7 to -66.6)

Location	Mortality (95% UI)		
	2017 counts	2017 age-standardised rates per 100,000	Percentage change in age-standardised rates between 1990 and 2017
France	4 053 (3 815 to 4 346)	5.5 (5.1 to 5.9)	-70.4 (-72.4 to -68.0)
Germany	4 716 (4 218 to 5 274)	4.5 (4.0 to 5.0)	-67.9 (-71.3 to -63.8)
Greece	1 221 (1 144 to 1 302)	9.9 (9.2 to 10.6)	-49.7 (-53.0 to -46.2)
Iceland	14 (13 to 15)	3.7 (3.4 to 4.0)	-67.3 (-70.1 to -64.3)
Ireland	188 (174 to 203)	3.5 (3.2 to 3.8)	-74.2 (-76.3 to -71.8)
Israel	632 (590 to 678)	6.3 (5.9 to 6.8)	-42.5 (-46.6 to -38.1)
Italy	5 710 (5 333 to 6 090)	6.1 (5.7 to 6.5)	-63.6 (-66.0 to -60.9)
Luxembourg	38 (34 to 42)	5.4 (4.8 to 6.0)	-71.7 (-74.8 to -68.5)
Malta	17 (16 to 19)	3.4 (3.2 to 3.7)	-52.9 (-56.9 to -48.2)
Netherlands	856 (807 to 909)	3.7 (3.5 to 4.0)	-63.3 (-65.8 to -60.5)
Norway	215 (208 to 223)	3.4 (3.3 to 3.6)	-64.9 (-66.3 to -63.1)
Portugal	961 (892 to 1 032)	6.5 (6.0 to 7.1)	-79.7 (-81.3 to -77.9)
Spain	2 452 (2 299 to 2 625)	4.2 (3.9 to 4.5)	-79.6 (-81.0 to -77.9)
Sweden	390 (365 to 418)	3.1 (2.9 to 3.4)	-67.9 (-70.2 to -65.5)
Switzerland	334 (310 to 365)	3.1 (2.8 to 3.3)	-77.5 (-79.3 to -75.2)
United Kingdom	2 679 (2 618 to 2 766)	3.5 (3.4 to 3.6)	-63.2 (-64.2 to -61.9)
**Latin America and Caribbean**	**111 293 (106 737 to 115 259)**	**18.6 (17.9 to 19.3)**	**-35.8 (-38.5 to -32.9)**
**Andean Latin America**	**11 169 (10 159 to 12 189)**	**18.8 (17.1 to 20.4)**	**-28.4 (-35.7 to -20.6)**
Bolivia	2 128 (1 567 to 2 609)	20.8 (15.8 to 25.4)	-53.4 (-65.4 to -40.7)
Ecuador	4 465 (4 018 to 4 977)	27.2 (24.5 to 30.3)	-10.4 (-19.8 to 0.2)
Peru	4 577 (3 901 to 5 316)	14.0 (12.0 to 16.3)	-26.2 (-37.8 to -12.4)
**Caribbean**	**10 539 (8 887 to 12 161)**	**22.1 (18.6 to 25.4)**	**-22.7 (-30.7 to -12.9)**
Antigua and Barbuda	7 (6 to 7)	7.0 (6.3 to 7.8)	-37.0 (-44.4 to -29.0)
The Bahamas	60 (54 to 67)	15.1 (13.6 to 16.9)	-29.0 (-37.3 to -19.3)
Barbados	31 (28 to 34)	8.9 (8.1 to 9.9)	-32.0 (-39.1 to -23.8)
Belize	72 (65 to 78)	19.2 (17.4 to 20.7)	-20.1 (-34.4 to -7.3)
Bermuda	7 (6 to 7)	8.0 (7.1 to 9.0)	-59.7 (-64.5 to -54.3)
Cuba	1 121 (998 to 1 276)	8.1 (7.2 to 9.2)	-61.7 (-66.1 to -56.4)
Dominica	12 (11 to 13)	15.7 (14.3 to 17.3)	-13.5 (-22.8 to -3.3)
Dominican Republic	3 152 (2 644 to 3 659)	30.0 (25.2 to 34.9)	35.5 (11.9 to 64.0)
Grenada	12 (11 to 13)	9.6 (8.7 to 10.4)	-41.6 (-47.4 to -35.0)
Guyana	118 (101 to 137)	15.9 (13.7 to 18.5)	-15.5 (-28.3 to -1.2)

Location	Mortality (95% UI)		
	2017 counts	2017 age-standardised rates per 100,000	Percentage change in age-standardised rates between 1990 and 2017
Haiti	4 487 (3 029 to 6 032)	42.9 (28.3 to 58.7)	-39.8 (-52.0 to -23.7)
Jamaica	277 (222 to 330)	9.3 (7.5 to 11.1)	94.2 (52.3 to 134.1)
Puerto Rico	446 (411 to 483)	10.1 (9.3 to 11.0)	-47.9 (-52.5 to -43.0)
Saint Lucia	25 (22 to 28)	12.9 (11.5 to 14.3)	-39.9 (-47.0 to -31.9)
Saint Vincent and the Grenadines	12 (11 to 13)	9.5 (8.6 to 10.4)	-18.2 (-27.3 to -7.9)
Suriname	99 (86 to 112)	16.8 (14.7 to 19.0)	-28.6 (-39.1 to -17.6)
Trinidad and Tobago	210 (171 to 255)	13.9 (11.3 to 16.8)	-13.8 (-29.6 to 5.1)
Virgin Islands	15 (13 to 17)	11.2 (9.8 to 12.7)	-36.1 (-46.0 to -24.2)
**Central Latin America**	**41 811 (39 363 to 43 874)**	**16.4 (15.4 to 17.2)**	**-38.4 (-42.5 to -35.3)**
Colombia	7 437 (6 572 to 8 381)	14.2 (12.6 to 16.0)	-41.6 (-49.0 to -33.8)
Costa Rica	782 (704 to 860)	15.7 (14.1 to 17.2)	-20.1 (-28.1 to -11.1)
El Salvador	1 282 (1 061 to 1 554)	21.4 (17.7 to 25.9)	-34.4 (-46.2 to -19.6)
Guatemala	2 692 (2 351 to 3 087)	17.4 (15.1 to 19.8)	-8.7 (-21.1 to 4.6)
Honduras	1 294 (979 to 1 581)	16.5 (12.3 to 20.2)	-30.7 (-46.8 to -11.2)
Mexico	20 170 (19 427 to 20 909)	16.0 (15.4 to 16.6)	-41.5 (-44.3 to -39.2)
Nicaragua	654 (558 to 766)	11.0 (9.5 to 12.8)	-50.6 (-58.2 to -42.5)
Panama	512 (469 to 557)	12.9 (11.8 to 14.0)	-41.8 (-47.2 to -36.3)
Venezuela	6 988 (5 856 to 8 378)	22.0 (18.5 to 26.3)	-30.0 (-41.6 to -16.7)
**Tropical Latin America**	**47 773 (45 698 to 49 554)**	**20.4 (19.6 to 21.2)**	**-37.0 (-39.8 to -33.6)**
Brazil	46 282 (44 196 to 47 990)	20.4 (19.5 to 21.1)	-37.9 (-40.7 to -34.4)
Paraguay	1 491 (1 221 to 1 816)	22.3 (18.3 to 27.1)	31.1 (5.1 to 63.6)
**North Africa and Middle East**	**131 692 (115 130 to 152 258)**	**23.2 (20.1 to 27.0)**	**-43.9 (-50.8 to -35.6)**
**North Africa and Middle East**	**131 692 (115 130 to 152 258)**	**23.2 (20.1 to 27.0)**	**-43.9 (-50.8 to -35.6)**
Afghanistan	8 692 (6 911 to 10 727)	33.3 (26.8 to 40.8)	-36.3 (-55.9 to 83.5)
Algeria	6 905 (5 516 to 11 141)	17.4 (13.8 to 28.4)	-50.8 (-58.3 to -41.9)
Bahrain	128 (113 to 147)	9.8 (8.7 to 11.0)	-60.4 (-65.0 to -54.0)
Egypt	26 946 (19 277 to 33 875)	31.9 (21.7 to 41.0)	-39.5 (-53.4 to -26.9)
Iran	21 124 (20 681 to 22 147)	26.1 (25.6 to 27.4)	-51.7 (-59.6 to -46.7)
Iraq	3 773 (3 433 to 4 205)	9.5 (8.7 to 10.5)	-64.6 (-70.6 to -49.8)
Jordan	1 110 (989 to 1 249)	11.7 (10.4 to 13.1)	-54.9 (-61.7 to -46.2)
Kuwait	529 (477 to 575)	13.9 (12.4 to 15.1)	-48.5 (-52.8 to -43.6)
Lebanon	562 (376 to 689)	6.9 (4.5 to 8.3)	-47.8 (-67.0 to -33.8)

Location	Mortality (95% UI)		
	2017 counts	2017 age-standardised rates per 100,000	Percentage change in age-standardised rates between 1990 and 2017
Libya	1 701 (871 to 2 607)	25.3 (13.0 to 39.2)	-27.9 (-55.1 to -5.6)
Morocco	7 264 (5 384 to 11 891)	20.6 (15.2 to 33.9)	-43.3 (-57.3 to -28.0)
Palestine	355 (313 to 407)	8.5 (7.7 to 9.5)	-50.1 (-58.1 to -39.6)
Oman	1 950 (1 572 to 2 346)	47.1 (38.4 to 56.1)	-52.4 (-63.2 to -38.6)
Qatar	574 (462 to 704)	24.8 (20.1 to 30.4)	-33.4 (-48.8 to -13.4)
Saudi Arabia	12 039 (8 422 to 14 884)	36.7 (25.9 to 44.1)	-28.2 (-56.2 to -4.2)
Sudan	10 692 (8 170 to 15 862)	30.4 (22.9 to 47.3)	-55.5 (-66.0 to -38.1)
Syria	1 748 (1 418 to 2 110)	11.6 (9.3 to 13.8)	-45.6 (-60.7 to -29.1)
Tunisia	3 669 (2 913 to 4 525)	30.2 (24.1 to 37.1)	-38.7 (-55.2 to -20.6)
Turkey	8 604 (7 763 to 9 520)	10.3 (9.3 to 11.4)	-38.7 (-47.5 to -28.0)
United Arab Emirates	3 649 (2 803 to 4 596)	49.9 (39.5 to 61.1)	-17.2 (-45.1 to 17.2)
Yemen	9 556 (7 228 to 13 629)	38.4 (28.8 to 56.0)	-44.8 (-60.1 to 12.0)
**South Asia**	**290 540 (253 208 to 313 209)**	**17.9 (15.4 to 19.3)**	**-2.8 (-14.0 to 7.0)**
**South Asia**	**290 540 (253 208 to 313 209)**	**17.9 (15.4 to 19.3)**	**-2.8 (-14.0 to 7.0)**
Bangladesh	11 798 (9 120 to 13 748)	8.2 (6.5 to 9.6)	4.9 (-16.6 to 35.8)
Bhutan	70 (51 to 86)	7.9 (5.9 to 9.6)	-57.4 (-68.0 to -43.7)
India	218 876 (201 734 to 231 141)	17.2 (15.7 to 18.1)	-9.2 (-18.3 to -0.6)
Nepal	6 787 (3 920 to 10 235)	26.6 (15.2 to 39.3)	12.7 (-21.9 to 55.9)
Pakistan	53 009 (32 258 to 69 115)	29.7 (17.9 to 38.8)	29.8 (2.8 to 63.0)
**Southeast Asia, East Asia, and Oceania**	**393 363 (374 675 to 408 773)**	**16.3 (15.6 to 16.9)**	**-27.2 (-34.3 to -22.1)**
**East Asia**	**275 976 (261 267 to 288 588)**	**15.6 (14.9 to 16.3)**	**-22.4 (-31.0 to -15.9)**
China	261 802 (247 924 to 273 651)	15.6 (14.8 to 16.2)	-21.8 (-30.7 to -14.9)
North Korea	5 744 (3 772 to 8 746)	20.1 (13.5 to 30.5)	36.2 (-1.6 to 80.1)
Taiwan (Province of China)	3 984 (3 752 to 4 257)	13.1 (12.4 to 14.0)	-66.6 (-68.6 to -64.4)
**Oceania**	**3 373 (2 747 to 4 069)**	**29.9 (24.7 to 35.7)**	**-19.7 (-35.2 to -2.2)**
American Samoa	4 (4 to 5)	8.9 (8.1 to 9.6)	-27.7 (-40.3 to -15.7)
Federated States of Micronesia	16 (11 to 21)	17.6 (12.2 to 22.1)	-17.9 (-43.0 to 8.7)
Fiji	84 (73 to 97)	10.0 (8.7 to 11.4)	-21.1 (-38.4 to -3.7)
Guam	23 (20 to 25)	13.6 (12.2 to 15.0)	-16.5 (-29.5 to -4.1)
Kiribati	12 (9 to 15)	11.2 (8.8 to 13.5)	-7.7 (-29.3 to 16.7)
Marshall Islands	12 (9 to 15)	24.5 (19.4 to 29.4)	-14.4 (-31.3 to 5.5)
Northern Mariana Islands	5 (4 to 6)	11.1 (9.7 to 12.4)	-37.5 (-52.0 to -20.3)

Location	Mortality (95% UI)		
	2017 counts	2017 age-standardised rates per 100,000	Percentage change in age-standardised rates between 1990 and 2017
Papua New Guinea	2 831<br>(2 243 to 3 484)	34.7<br>(28.0 to 42.2)	-26.6<br>(-42.1 to -8.6)
Samoa	18<br>(14 to 25)	10.9<br>(8.7 to 15.2)	-16.0<br>(-34.5 to 5.0)
Solomon Islands	117<br>(91 to 149)	21.7<br>(16.8 to 27.8)	-16.3<br>(-35.0 to 5.3)
Tonga	11<br>(9 to 13)	12.1<br>(10.1 to 14.0)	-7.8<br>(-32.2 to 14.5)
Vanuatu	52<br>(36 to 72)	21.0<br>(14.8 to 28.6)	-5.2<br>(-30.5 to 31.7)
**Southeast Asia**	**114 014<br>(106 883 to 120 762)**	**17.3<br>(16.3 to 18.4)**	**-41.1<br>(-46.3 to -36.4)**
Cambodia	3 981<br>(3 155 to 5 142)	27.7<br>(22.1 to 35.2)	-40.5<br>(-52.3 to -22.6)
Indonesia	35 626<br>(32 587 to 38 492)	14.5<br>(13.2 to 15.7)	-51.2<br>(-56.4 to -46.5)
Laos	1 690<br>(1 275 to 2 112)	26.8<br>(20.5 to 33.5)	-45.1<br>(-58.2 to -28.4)
Malaysia	6 946<br>(6 127 to 7 794)	23.3<br>(20.6 to 26.1)	-26.2<br>(-42.8 to -11.9)
Maldives	33<br>(25 to 60)	8.4<br>(6.3 to 14.7)	-64.7<br>(-70.1 to -52.0)
Mauritius	165<br>(150 to 181)	11.4<br>(10.4 to 12.5)	-22.5<br>(-30.2 to -14.3)
Myanmar	10 942<br>(9 203 to 13 089)	20.7<br>(17.6 to 24.6)	-47.5<br>(-58.0 to -34.7)
Philippines	10 940<br>(9 482 to 12 600)	11.6<br>(10.1 to 13.3)	4.8<br>(-10.5 to 23.1)
Sri Lanka	2 795<br>(2 318 to 3 320)	12.3<br>(10.2 to 14.6)	-19.9<br>(-33.8 to -3.0)
Seychelles	14<br>(12 to 16)	13.4<br>(11.6 to 15.0)	15.0<br>(1.2 to 28.8)
Thailand	19 183<br>(16 832 to 21 609)	24.7<br>(21.8 to 27.5)	-41.9<br>(-52.6 to -31.9)
Timor-Leste	118<br>(67 to 200)	10.5<br>(6.2 to 18.3)	-31.8<br>(-56.6 to -11.3)
Vietnam	21 431<br>(17 934 to 24 368)	21.4<br>(18.2 to 24.2)	-24.3<br>(-39.7 to -6.9)
**Sub-Saharan Africa**	**161 647<br>(150 086 to 173 753)**	**22.0<br>(20.5 to 23.6)**	**-31.3<br>(-38.7 to -22.7)**
**Central sub-Saharan Africa**	**32 666<br>(27 105 to 38 467)**	**31.0<br>(26.4 to 36.3)**	**-29.0<br>(-40.3 to -7.3)**
Angola	6 781<br>(5 592 to 8 207)	28.6<br>(23.5 to 34.9)	-50.1<br>(-61.8 to -16.5)
Central African Republic	3 495<br>(1 985 to 4 570)	85.5<br>(50.7 to 111.2)	5.6<br>(-21.9 to 62.3)
Congo (Brazzaville)	1 229<br>(944 to 1 552)	28.6<br>(22.4 to 35.4)	-43.6<br>(-56.3 to -26.7)
DR Congo	20 502<br>(15 783 to 25 532)	28.9<br>(23.1 to 35.8)	-20.8<br>(-36.3 to 9.4)
Equatorial Guinea	225<br>(154 to 319)	20.9<br>(14.3 to 30.2)	-68.0<br>(-78.2 to -54.1)
Gabon	435<br>(342 to 548)	28.5<br>(22.7 to 35.7)	-34.7<br>(-49.4 to -18.5)
**Eastern sub-Saharan Africa**	**52 980<br>(46 622 to 58 814)**	**21.5<br>(18.9 to 23.5)**	**-35.0<br>(-43.8 to -23.2)**
Burundi	2 287<br>(1 799 to 3 045)	35.1<br>(27.7 to 46.6)	-33.8<br>(-47.7 to -15.3)
Comoros	113<br>(94 to 137)	20.7<br>(17.2 to 24.8)	-42.9<br>(-55.0 to -27.9)
Djibouti	175<br>(125 to 251)	23.0<br>(16.6 to 32.0)	-27.2<br>(-47.9 to 4.7)
Eritrea	1 287<br>(913 to 1 627)	33.0<br>(24.6 to 40.6)	-40.2<br>(-54.2 to -10.5)

Location	Mortality (95% UI)		
	2017 counts	2017 age-standardised rates per 100,000	Percentage change in age-standardised rates between 1990 and 2017
Ethiopia	9 742<br>(8 867 to 10 837)	15.4<br>(14.0 to 16.9)	-57.2<br>(-64.7 to -40.4)
Kenya	5 503<br>(5 015 to 6 334)	18.2<br>(16.5 to 20.6)	-12.9<br>(-41.0 to 2.7)
Madagascar	3 475<br>(2 827 to 4 271)	20.8<br>(17.0 to 25.1)	-35.8<br>(-47.5 to -21.7)
Malawi	2 227<br>(1 865 to 2 628)	19.4<br>(16.7 to 22.4)	-45.6<br>(-63.4 to 13.2)
Mozambique	5 078<br>(4 228 to 5 948)	27.5<br>(23.3 to 31.9)	-17.0<br>(-36.1 to 4.8)
Rwanda	2 661<br>(1 822 to 3 743)	33.3<br>(22.7 to 44.5)	-45.2<br>(-57.4 to -27.8)
Somalia	5 154<br>(2 772 to 7 410)	51.1<br>(27.8 to 72.0)	0.8<br>(-34.2 to 86.7)
South Sudan	1 761<br>(1 291 to 2 405)	28.3<br>(20.9 to 39.0)	-11.2<br>(-38.3 to 48.2)
Tanzania	5 560<br>(4 790 to 6 411)	15.4<br>(13.4 to 17.7)	-32.6<br>(-47.5 to 1.0)
Uganda	5 826<br>(4 239 to 7 496)	26.1<br>(18.9 to 33.3)	1.3<br>(-29.9 to 34.6)
Zambia	2 098<br>(1 794 to 2 435)	19.8<br>(17.2 to 22.6)	-45.1<br>(-57.3 to -23.3)
**Southern sub-Saharan Africa**	**20 157<br>(18 838 to 21 651)**	**27.6<br>(26.0 to 29.5)**	**-37.0<br>(-46.3 to -30.3)**
Botswana	299<br>(253 to 341)	15.1<br>(12.7 to 17.1)	-32.7<br>(-45.8 to -15.2)
Lesotho	803<br>(635 to 981)	45.0<br>(35.7 to 54.4)	20.9<br>(-18.9 to 60.7)
Namibia	451<br>(357 to 572)	22.0<br>(17.7 to 27.5)	-36.2<br>(-50.1 to -19.7)
South Africa	15 504<br>(14 441 to 16 826)	28.2<br>(26.5 to 30.4)	-44.2<br>(-50.5 to -38.8)
Swaziland	371<br>(284 to 460)	37.7<br>(28.8 to 46.2)	1.5<br>(-33.3 to 35.6)
Zimbabwe	2 730<br>(1 939 to 3 715)	25.4<br>(18.4 to 34.2)	7.8<br>(-32.2 to 42.0)
**Western sub-Saharan Africa**	**55 845<br>(49 708 to 62 806)**	**18.7<br>(16.8 to 21.0)**	**-24.1<br>(-34.2 to -12.6)**
Benin	3 093<br>(1 880 to 4 280)	41.6<br>(25.0 to 56.0)	-31.0<br>(-43.6 to -15.1)
Burkina Faso	3 497<br>(2 885 to 4 178)	24.3<br>(20.2 to 28.1)	-12.4<br>(-29.6 to 9.9)
Cameroon	4 108<br>(3 215 to 5 192)	23.0<br>(18.3 to 28.4)	-34.1<br>(-49.8 to -16.7)
Cape Verde	43<br>(37 to 49)	8.4<br>(7.2 to 9.5)	12.3<br>(-4.4 to 32.1)
Chad	2 602<br>(2 107 to 3 328)	25.5<br>(20.7 to 32.6)	10.9<br>(-10.6 to 35.9)
Cote d'Ivoire	3 631<br>(3 055 to 4 329)	22.1<br>(18.8 to 25.8)	-20.0<br>(-36.9 to -1.3)
The Gambia	286<br>(218 to 360)	21.1<br>(16.8 to 25.3)	-8.0<br>(-30.2 to 16.8)
Ghana	5 381<br>(4 579 to 6 363)	24.4<br>(21.1 to 28.0)	-3.7<br>(-28.5 to 20.7)
Guinea	1 978<br>(1 667 to 2 329)	24.4<br>(20.7 to 28.7)	-20.9<br>(-36.6 to -2.7)
Guinea-Bissau	388<br>(304 to 488)	32.6<br>(27.2 to 39.3)	-42.1<br>(-54.0 to -27.6)
Liberia	499<br>(406 to 641)	16.0<br>(13.1 to 19.2)	-39.1<br>(-51.3 to -24.0)
Mali	3 109<br>(2 493 to 3 997)	20.1<br>(16.5 to 26.8)	-46.1<br>(-56.1 to -31.4)
Mauritania	674<br>(571 to 785)	23.4<br>(19.3 to 27.4)	-44.7<br>(-53.5 to -33.3)

Location	Mortality (95% UI)		
	2017 counts	2017 age-standardised rates per 100,000	Percentage change in age-standardised rates between 1990 and 2017
Niger	2 542 (1 856 to 3 410)	18.1 (13.3 to 24.3)	-34.4 (-45.7 to -17.5)
Nigeria	19 810 (13 745 to 25 758)	13.5 (9.8 to 18.0)	-28.3 (-46.2 to -3.2)
Sao Tome and Principe	24 (15 to 30)	15.9 (9.9 to 20.2)	8.4 (-24.8 to 40.5)
Senegal	1 800 (1 454 to 2 679)	17.7 (14.5 to 24.7)	-19.6 (-33.3 to -1.7)
Sierra Leone	1 245 (1 041 to 1 506)	22.7 (19.2 to 27.5)	-20.5 (-35.4 to 2.4)
Togo	1 135 (917 to 1 406)	22.5 (18.5 to 27.1)	-16.1 (-38.6 to 8.0)
