# Supplementary material for: Morbidity and mortality from road injuries: results from the Global Burden of Disease Study 2017
Source: Inj Prev. 2020 Jan 8;26(Suppl 1):i46–56. doi: 10.1136/injuryprev-2019-043302 (PMC7571357; doi:10.1136/injuryprev-2019-043302)
Supplement: Supplementary data [file injuryprev-2019-043302supp005.pdf]

Table 3: YLLs, YLDs, and DALYs for 2017 and percentage change of age-standardised rates between 1990 and 2017 by location for road injuries

Location	YLLs (95% UI)			YLDs (95% UI)			DALYs (95% UI)		
	2017 counts	2017 age-standardised rates per 100,000	Percentage change in age-standardised rates between 1990 and 2017	2017 counts	2017 age-standardised rates per 100,000	Percentage change in age-standardised rates between 1990 and 2017	2017 counts	2017 age-standardised rates per 100,000	Percentage change in age-standardised rates between 1990 and 2017
Global	57 638 366<br>(55 500 786 to 59 369 191)	745<br>(718 to 767)	-34.4<br>(-38.5 to -30.4)	10 159 667<br>(7 272 042 to 13 618 818)	126<br>(90 to 169)	2.2<br>(0.3 to 4.0)	67 798 033<br>(64 337 599 to 71 454 968)	871<br>(828 to 917)	-30.8<br>(-35.0 to -26.9)
Low SDI	10 859 153<br>(10 166 644 to 11 594 703)	886<br>(834 to 942)	-24.5<br>(-33.2 to -7.0)	881 984<br>(637 927 to 1 173 872)	90<br>(66 to 119)	-2.2<br>(-4.2 to -0.1)	11 741 137<br>(10 984 026 to 12 569 333)	976<br>(917 to 1 041)	-22.8<br>(-31.3 to -6.5)
Low-middle SDI	15 605 391<br>(14 473 161 to 16 583 346)	923<br>(855 to 979)	-27.3<br>(-33.6 to -20.1)	1 490 436<br>(1 076 899 to 1 976 281)	101<br>(73 to 134)	15.1<br>(12.3 to 18.3)	17 095 827<br>(15 944 263 to 18 150 896)	1 023<br>(950 to 1 086)	-24.6<br>(-30.9 to -17.8)
Middle SDI	16 782 061<br>(16 097 262 to 17 329 206)	769<br>(740 to 794)	-34.5<br>(-39.4 to -30.8)	2 768 239<br>(1 984 422 to 3 717 378)	120<br>(86 to 161)	30.2<br>(25.5 to 35.2)	19 550 300<br>(18 517 456 to 20 647 763)	889<br>(844 to 936)	-29.8<br>(-34.6 to -26.0)
High-middle SDI	10 209 177<br>(9 792 901 to 10 567 843)	705<br>(678 to 727)	-40.0<br>(-44.4 to -36.5)	2 663 875<br>(1 915 102 to 3 602 346)	154<br>(111 to 209)	9.2<br>(6.4 to 12.1)	12 873 052<br>(12 004 007 to 13 868 911)	859<br>(806 to 917)	-34.8<br>(-39.0 to -31.1)
High SDI	3 957 305<br>(3 869 315 to 4 056 500)	359<br>(350 to 368)	-59.3<br>(-60.4 to -58.1)	2 302 189<br>(1 647 361 to 3 101 989)	151<br>(107 to 203)	-15.5<br>(-16.4 to -14.6)	6 259 494<br>(5 596 961 to 7 053 358)	509<br>(465 to 562)	-51.9<br>(-54.0 to -49.8)
Central Europe, Eastern Europe, and Central Asia	2 467 734<br>(2 411 509 to 2 533 581)	592<br>(578 to 607)	-46.8<br>(-48.1 to -45.1)	959 498<br>(681 404 to 1 295 813)	184<br>(131 to 248)	-17.2<br>(-18.9 to -15.4)	3 427 231<br>(3 153 810 to 3 762 701)	775<br>(722 to 840)	-41.8<br>(-43.6 to -39.9)
Central Asia	535 500<br>(503 802 to 571 613)	566<br>(534 to 604)	-42.2<br>(-45.6 to -38.2)	118 437<br>(84 146 to 158 821)	133<br>(95 to 179)	-13.2<br>(-15.3 to -11.0)	653 937<br>(605 871 to 705 626)	700<br>(647 to 756)	-38.2<br>(-41.6 to -34.7)
Armenia	10 429<br>(9 780 to 11 166)	328<br>(307 to 351)	-58.9<br>(-62.0 to -55.5)	3 752<br>(2 678 to 5 063)	103<br>(73 to 138)	-22.6<br>(-25.2 to -20.2)	14 181<br>(12 951 to 15 697)	431<br>(395 to 473)	-53.7<br>(-56.8 to -50.5)
Azerbaijan	30 275<br>(26 561 to 34 656)	282<br>(246 to 322)	-63.8<br>(-68.8 to -58.2)	12 131<br>(8 631 to 16 237)	111<br>(79 to 149)	-24.2<br>(-26.7 to -21.5)	42 405<br>(36 829 to 48 269)	394<br>(342 to 447)	-57.5<br>(-62.4 to -52.5)
Georgia	31 281<br>(28 765 to 33 468)	843<br>(774 to 901)	-15.2<br>(-22.5 to -7.7)	7 508<br>(5 328 to 10 142)	161<br>(114 to 217)	3.4<br>(1.3 to 5.5)	38 789<br>(35 490 to 42 105)	1 004<br>(924 to 1 081)	-12.7<br>(-19.2 to -6.2)
Kazakhstan	138 147<br>(126 987 to 150 230)	745<br>(685 to 809)	-37.8<br>(-42.8 to -31.8)	30 789<br>(21 882 to 41 541)	167<br>(118 to 225)	-8.2<br>(-11.0 to -5.4)	168 937<br>(154 680 to 183 426)	911<br>(835 to 990)	-33.9<br>(-38.6 to -28.8)
Kyrgyzstan	44 418<br>(41 468 to 47 903)	690<br>(644 to 742)	-48.4<br>(-52.7 to -43.6)	7 437<br>(5 321 to 9 963)	132<br>(94 to 176)	-23.8<br>(-25.5 to -22.1)	51 855<br>(48 365 to 55 712)	821<br>(763 to 885)	-45.6<br>(-49.6 to -41.2)
Mongolia	28 237<br>(24 670 to 32 344)	815<br>(717 to 930)	6.2<br>(-14.5 to 30.5)	5 105<br>(3 651 to 6 791)	163<br>(116 to 217)	17.6<br>(13.3 to 21.9)	33 342<br>(29 640 to 37 446)	978<br>(871 to 1 093)	8.0<br>(-10.4 to 28.4)
Tajikistan	35 082<br>(31 233 to 39 326)	372<br>(332 to 417)	-47.0<br>(-53.5 to -39.3)	7 213<br>(5 178 to 9 649)	95<br>(68 to 127)	-22.5<br>(-24.6 to -20.5)	42 295<br>(37 880 to 47 158)	468<br>(418 to 521)	-43.3<br>(-49.2 to -36.7)
Turkmenistan	16 870<br>(15 029 to 19 187)	323<br>(289 to 368)	-66.5<br>(-70.7 to -61.7)	5 786<br>(4 103 to 7 796)	120<br>(85 to 162)	-22.2<br>(-24.9 to -19.2)	22 656<br>(19 945 to 25 610)	443<br>(390 to 502)	-60.4<br>(-64.7 to -55.7)
Uzbekistan	200 760<br>(175 455 to 228 368)	599<br>(524 to 681)	-33.8<br>(-42.4 to -24.3)	38 716<br>(27 532 to 51 867)	129<br>(92 to 173)	-5.4<br>(-8.3 to -2.4)	239 476<br>(210 570 to 270 548)	728<br>(643 to 820)	-30.1<br>(-37.8 to -21.7)
Central Europe	431 549<br>(417 252 to 447 596)	377<br>(364 to 392)	-58.5<br>(-60.1 to -56.7)	343 646<br>(245 006 to 461 514)	225<br>(159 to 303)	-12.5<br>(-14.2 to -10.7)	775 195<br>(676 651 to 893 787)	602<br>(537 to 679)	-48.4<br>(-51.2 to -45.3)
Albania	10 926<br>(8 804 to 13 492)	389<br>(313 to 478)	-39.2<br>(-51.6 to -24.1)	6 810<br>(4 848 to 9 162)	198<br>(141 to 267)	7.5<br>(3.4 to 11.9)	17 736<br>(14 573 to 21 288)	588<br>(485 to 704)	-28.7<br>(-39.8 to -16.7)
Bosnia and Herzegovina	9 965<br>(9 003 to 10 955)	285<br>(258 to 315)	10.3<br>(-12.2 to 28.4)	8 953<br>(6 384 to 12 028)	200<br>(141 to 270)	48.4<br>(42.2 to 54.8)	18 917<br>(16 040 to 22 029)	486<br>(419 to 559)	23.4<br>(5.7 to 36.7)
Bulgaria	28 235<br>(26 031 to 30 827)	428<br>(391 to 468)	-42.5<br>(-47.5 to -37.1)	21 295<br>(15 164 to 28 848)	217<br>(154 to 295)	-0.9<br>(-3.4 to 1.6)	49 529<br>(42 889 to 56 993)	646<br>(572 to 730)	-33.0<br>(-37.8 to -28.3)
Croatia	14 460<br>(13 383 to 15 618)	350<br>(323 to 378)	-61.8<br>(-65.0 to -58.4)	14 689<br>(10 353 to 19 625)	254<br>(180 to 342)	-11.2<br>(-12.9 to -9.4)	29 149<br>(24 780 to 34 041)	604<br>(529 to 690)	-49.7<br>(-53.5 to -46.1)
Czech Republic	31 491<br>(29 196 to 33 952)	307<br>(284 to 331)	-56.2<br>(-59.7 to -52.2)	37 482<br>(26 758 to 50 022)	264<br>(187 to 355)	-0.1<br>(-2.1 to 2.0)	68 973<br>(58 226 to 81 722)	571<br>(492 to 663)	-40.8<br>(-45.3 to -36.3)
Hungary	29 004<br>(26 822 to 31 891)	287<br>(265 to 315)	-71.1<br>(-73.4 to -68.1)	25 828<br>(18 491 to 34 770)	197<br>(140 to 266)	-24.0<br>(-25.9 to -22.4)	54 832<br>(46 963 to 64 191)	484<br>(421 to 557)	-61.3<br>(-64.5 to -57.9)
Macedonia	6 879<br>(6 191 to 7 563)	317<br>(284 to 351)	-32.5<br>(-40.4 to -24.3)	5 327<br>(3 784 to 7 176)	193<br>(136 to 260)	3.0<br>(-0.1 to 6.6)	12 206<br>(10 454 to 14 097)	509<br>(444 to 583)	-22.4<br>(-29.1 to -15.8)
Montenegro	2 262<br>(2 039 to 2 527)	355<br>(318 to 397)	-34.8<br>(-43.1 to -24.9)	1 698<br>(1 206 to 2 284)	216<br>(153 to 291)	1.3<br>(-0.7 to 3.2)	3 960<br>(3 408 to 4 605)	571<br>(496 to 659)	-24.7<br>(-31.6 to -16.7)
Poland	160 185<br>(149 273 to 171 897)	414<br>(384 to 446)	-62.2<br>(-65.0 to -58.9)	122 364<br>(87 391 to 164 610)	240<br>(170 to 322)	-14.8<br>(-17.2 to -12.4)	282 549<br>(246 978 to 325 777)	654<br>(579 to 741)	-52.5<br>(-55.9 to -48.9)
Romania	85 556<br>(80 033 to 91 665)	449<br>(419 to 482)	-53.4<br>(-56.7 to -50.2)	54 275<br>(38 542 to 73 053)	206<br>(146 to 278)	-17.4<br>(-20.2 to -14.8)	139 831<br>(122 190 to 159 522)	655<br>(585 to 736)	-46.0<br>(-49.3 to -42.6)
Serbia	28 931<br>(26 570 to 31 268)	313<br>(288 to 339)	-59.8<br>(-64.7 to -52.9)	22 100<br>(15 724 to 29 615)	192<br>(136 to 257)	-21.6<br>(-23.5 to -19.8)	51 032<br>(44 349 to 59 205)	505<br>(446 to 578)	-50.7<br>(-55.6 to -44.5)
Slovakia	18 115<br>(16 556 to 20 200)	335<br>(305 to 373)	-65.7<br>(-69.1 to -61.4)	15 426<br>(10 991 to 20 767)	218<br>(155 to 295)	-20.6<br>(-22.3 to -19.0)	33 541<br>(28 872 to 38 859)	553<br>(485 to 633)	-55.8<br>(-59.5 to -51.6)
Slovenia	5 538<br>(5 066 to 6 083)	293<br>(267 to 322)	-74.6<br>(-77.0 to -71.8)	7 400<br>(5 272 to 9 903)	263<br>(186 to 354)	-22.1<br>(-23.8 to -20.5)	12 939<br>(10 748 to 15 441)	555<br>(474 to 651)	-62.7<br>(-66.3 to -59.1)
Eastern Europe	1 500 685<br>(1 461 098 to 1 549 538)	727<br>(708 to 750)	-42.9<br>(-44.6 to -40.8)	497 415<br>(351 139 to 669 519)	179<br>(126 to 242)	-18.2<br>(-20.3 to -16.1)	1 998 100<br>(1 849 019 to 2 175 598)	906<br>(851 to 972)	-39.2<br>(-41.1 to -37.2)
Belarus	40 870<br>(37 451 to 45 090)	418<br>(381 to 464)	-63.8<br>(-67.1 to -58.6)	19 858<br>(14 097 to 26 702)	157<br>(112 to 212)	-21.4<br>(-23.4 to -19.4)	60 728<br>(54 098 to 68 746)	576<br>(517 to 645)	-57.5<br>(-60.8 to -52.3)
Estonia	3 585<br>(3 095 to 4 126)	283<br>(244 to 326)	-79.8<br>(-82.6 to -76.5)	2 949<br>(2 082 to 3 954)	166<br>(117 to 223)	-29.3<br>(-30.9 to -27.8)	6 534<br>(5 586 to 7 652)	448<br>(388 to 518)	-72.6<br>(-75.5 to -69.0)
Latvia	8 407<br>(7 385 to 9 509)	442<br>(387 to 499)	-73.6<br>(-77.1 to -69.8)	4 590<br>(3 240 to 6 173)	171<br>(121 to 230)	-33.1<br>(-34.6 to -31.6)	12 998<br>(11 350 to 14 721)	613<br>(539 to 684)	-68.2<br>(-71.7 to -64.8)
Lithuania	12 549<br>(11 548 to 13 781)	434<br>(397 to 482)	-69.1<br>(-71.9 to -65.4)	7 097<br>(5 021 to 9 525)	180<br>(127 to 241)	-24.3<br>(-26.0 to -22.6)	19 645<br>(17 388 to 22 367)	613<br>(552 to 690)	-62.7<br>(-65.6 to -59.3)
Moldova	19 561<br>(18 412 to 20 843)	518<br>(487 to 553)	-64.9<br>(-67.5 to -62.1)	6 670<br>(4 749 to 8 956)	140<br>(99 to 188)	-35.1<br>(-36.9 to -33.3)	26 231<br>(24 041 to 28 695)	658<br>(607 to 715)	-61.1<br>(-63.6 to -58.4)
Russian Federation	1 101 491<br>(1 071 703 to 1 168 545)	762<br>(741 to 809)	-41.4<br>(-43.2 to -37.9)	351 974<br>(248 744 to 474 396)	183<br>(129 to 248)	-19.8<br>(-22.0 to -17.6)	1 453 465<br>(1 348 124 to 1 586 429)	945<br>(887 to 1 017)	-38.2<br>(-40.0 to -35.6)
Ukraine	314 222<br>(272 873 to 342 100)	741<br>(635 to 811)	-36.2<br>(-45.1 to -30.0)	104 277<br>(73 446 to 140 800)	175<br>(122 to 235)	-9.3<br>(-11.6 to -6.9)	418 499<br>(365 862 to 464 710)	916<br>(798 to 1 007)	-32.4<br>(-40.2 to -26.9)
High-income	3 870 249<br>(3 766 283 to 3 980 052)	375<br>(364 to 386)	-56.2<br>(-57.6 to -54.9)	1 968 107<br>(1 404 036 to 2 653 942)	138<br>(98 to 186)	-18.9<br>(-19.7 to -18.0)	5 838 356<br>(5 261 833 to 6 518 216)	513<br>(472 to 561)	-50.1<br>(-52.0 to -48.0)
Australasia	84 307<br>(76 249 to 92 272)	311<br>(281 to 340)	-65.0<br>(-68.4 to -61.5)	74 635<br>(53 002 to 100 734)	206<br>(146 to 278)	-18.3<br>(-19.7 to -16.9)	158 942<br>(135 480 to 186 481)	517<br>(450 to 595)	-54.7<br>(-58.6 to -50.8)
Australia	68 047<br>(60 460 to 76 194)	295<br>(263 to 331)	-63.8<br>(-67.9 to -59.4)	58 789<br>(41 567 to 79 519)	193<br>(137 to 261)	-20.6<br>(-22.3 to -18.9)	126 836<br>(107 609 to 149 812)	489<br>(421 to 567)	-53.8<br>(-58.1 to -49.5)
New Zealand	16 261<br>(15 292 to 17 299)	396<br>(372 to 422)	-68.0<br>(-70.2 to -65.8)	15 846<br>(11 228 to 21 357)	276<br>(195 to 373)	-7.3<br>(-9.2 to -5.6)	32 107<br>(27 383 to 37 691)	672<br>(589 to 773)	-56.3<br>(-59.7 to -52.4)
High-income Asia-Pacific	423 496<br>(404 788 to 443 564)	212<br>(203 to 222)	-75.0<br>(-76.3 to -73.6)	324 999<br>(232 293 to 434 757)	117<br>(83 to 157)	-24.9<br>(-26.3 to -23.5)	748 495<br>(658 877 to 861 019)	329<br>(297 to 370)	-67.3<br>(-69.6 to -64.9)
Brunei	3 313<br>(2 911 to 3 703)	724<br>(642 to 804)	-49.1<br>(-56.1 to -42.0)	667<br>(468 to 901)	156<br>(110 to 212)	-32.9<br>(-34.8 to -31.2)	3 980<br>(3 542 to 4 469)	880<br>(793 to 981)	-46.9<br>(-53.1 to -40.6)
Japan	215 338<br>(206 926 to 225 334)	169<br>(162 to 176)	-67.9<br>(-69.2 to -66.5)	233 743<br>(167 533 to 311 576)	119<br>(85 to 160)	-16.9<br>(-18.6 to -14.9)	449 081<br>(382 427 to 528 530)	288<br>(253 to 330)	-57.0<br>(-59.8 to -53.9)
South Korea	196 375<br>(180 704 to 213 266)	323<br>(299 to 350)	-81.7<br>(-83.2 to -79.8)	83 032<br>(58 866 to 112 426)	115<br>(81 to 155)	-44.0<br>(-45.7 to -42.4)	279 407<br>(250 816 to 310 953)	437<br>(397 to 485)	-77.7<br>(-79.4 to -75.8)
Singapore	8 469<br>(7 770 to 9 217)	139<br>(127 to 151)	-64.8<br>(-68.1 to -61.7)	7 558<br>(5 342 to 10 234)	112<br>(79 to 152)	-3.5<br>(-6.3 to -0.7)	16 027<br>(13 647 to 18 746)	250<br>(215 to 291)	-50.9<br>(-55.6 to -46.4)
High-income North America	2 010 842<br>(1 917 839 to 2 091 726)	574<br>(548 to 597)	-40.5<br>(-43.5 to -37.9)	731 662<br>(528 529 to 977 972)	160<br>(115 to 214)	-21.9<br>(-23.2 to -20.5)	2 742 504<br>(2 526 022 to 3 003 613)	734<br>(682 to 795)	-37.2<br>(-39.9 to -35.0)
Canada	112 125<br>(103 699 to 120 452)	337<br>(311 to 362)	-57.8<br>(-61.2 to -54.4)	64 251<br>(45 490 to 87 044)	137<br>(96 to 185)	-21.9<br>(-23.8 to -20.0)	176 375<br>(156 565 to 199 944)	474<br>(426 to 528)	-51.3<br>(-54.6 to -48.1)
Greenland	109<br>(96 to 125)	198<br>(174 to 228)	-69.6<br>(-75.3 to -59.9)	53<br>(37 to 72)	81<br>(57 to 110)	-28.6<br>(-30.9 to -26.5)	162<br>(142 to 184)	280<br>(246 to 320)	-63.5<br>(-69.3 to -54.4)
USA	1 898 573<br>(1 805 915 to 1 977 120)	600<br>(571 to 625)	-39.0<br>(-42.2 to -36.2)	667 345<br>(482 369 to 891 856)	163<br>(117 to 218)	-21.8<br>(-23.2 to -20.3)	2 565 919<br>(2 365 666 to 2 807 024)	763<br>(709 to 826)	-36.0<br>(-38.8 to -33.7)
Southern Latin America	407 091<br>(371 351 to 447 236)	605<br>(552 to 664)	-10.7<br>(-18.7 to -1.3)	80 149<br>(56 954 to 108 380)	109<br>(77 to 148)	22.3<br>(18.5 to 26.5)	487 240<br>(442 127 to 535 364)	714<br>(649 to 782)	-6.9<br>(-14.1 to 1.7)
Argentina	289 424<br>(257 888 to 327 091)	641<br>(572 to 722)	-0.9<br>(-12.8 to 13.1)	56 713<br>(40 311 to 76 749)	117<br>(83 to 159)	29.2<br>(25.0 to 33.7)	346 137<br>(308 138 to 387 501)	758<br>(676 to 847)	2.8<br>(-7.9 to 15.1)
Chile	94 017<br>(82 122 to 106 924)	497<br>(437 to 564)	-32.8<br>(-40.8 to -23.3)	18 359<br>(13 052 to 24 940)	87<br>(62 to 118)	3.1<br>(-0.6 to 6.8)	112 376<br>(98 851 to 125 819)	584<br>(515 to 654)	-29.1<br>(-36.9 to -20.6)
Uruguay	23 631<br>(20 664 to 26 725)	676<br>(593 to 763)	-10.2<br>(-22.0 to 1.4)	5 074<br>(3 623 to 6 857)	123<br>(87 to 167)	34.1<br>(30.0 to 38.7)	28 705<br>(25 409 to 32 359)	800<br>(707 to 898)	-5.4<br>(-16.6 to 5.0)
Western Europe	944 513<br>(915 348 to 975 076)	228<br>(221 to 236)	-71.7<br>(-72.6 to -70.7)	756 662<br>(537 785 to 1 024 824)	130<br>(92 to 175)	-17.8<br>(-19.3 to -16.6)	1 701 176<br>(1 483 766 to 1 964 595)	358<br>(320 to 404)	-62.9<br>(-65.2 to -60.2)
Andorra	152<br>(129 to 178)	206<br>(175 to 241)	-57.8<br>(-66.8 to -46.4)	155<br>(109 to 210)	144<br>(102 to 196)	0.8<br>(-1.2 to 2.8)	306<br>(257 to 366)	350<br>(299 to 414)	-44.5<br>(-53.3 to -33.5)
Austria	18 049<br>(16 762 to 19 434)	212<br>(196 to 229)	-75.7<br>(-77.6 to -73.4)	15 931<br>(11 286 to 21 555)	135<br>(95 to 182)	-19.6<br>(-21.7 to -17.5)	33 980<br>(29 093 to 39 490)	347<br>(303 to 394)	-66.6<br>(-69.5 to -63.4)
Belgium	35 596<br>(33 108 to 38 177)	321<br>(297 to 345)	-68.7<br>(-71.1 to -66.3)	18 935<br>(13 424 to 25 680)	125<br>(88 to 169)	-28.1<br>(-30.0 to -26.3)	54 531<br>(48 375 to 61 664)	446<br>(403 to 496)	-62.8<br>(-65.5 to -59.9)
Cyprus	6 023<br>(5 434 to 6 685)	466<br>(419 to 518)	-59.5<br>(-65.9 to -53.6)	2 639<br>(1 872 to 3 570)	166<br>(118 to 224)	-24.7<br>(-26.4 to -23.1)	8 662<br>(7 649 to 9 838)	632<br>(562 to 709)	-53.9<br>(-60.3 to -48.5)
Denmark	10 470<br>(9 691 to 11 349)	188<br>(173 to 204)	-71.6<br>(-74.1 to -69.0)	9 666<br>(6 880 to 13 026)	130<br>(92 to 175)	-14.9<br>(-16.8 to -12.9)	20 136<br>(17 104 to 23 563)	318<br>(276 to 367)	-61.0<br>(-64.6 to -57.3)
Finland	10 431<br>(9 675 to 11 279)	200<br>(184 to 217)	-70.8<br>(-73.4 to -67.9)	10 286<br>(7 309 to 13 932)	139<br>(98 to 188)	-2.1<br>(-3.8 to -0.0)	20 717<br>(17 653 to 24 437)	338<br>(293 to 391)	-59.0<br>(-63.0 to -55.0)
France	163 837<br>(152 700 to 176 480)	278<br>(257 to 300)	-71.3<br>(-73.4 to -69.0)	121 899<br>(86 503 to 165 672)	143<br>(101 to 193)	-21.9<br>(-24.4 to -19.9)	285 736<br>(247 705 to 329 689)	420<br>(372 to 476)	-63.5<br>(-66.3 to -60.6)
Germany	172 459<br>(153 981 to 193 858)	219<br>(195 to 246)	-71.1<br>(-74.4 to -67.4)	151 476<br>(107 764 to 205 183)	132<br>(93 to 178)	-14.8<br>(-16.8 to -13.1)	323 934<br>(276 644 to 381 551)	351<br>(307 to 403)	-61.6<br>(-65.3 to -57.6)
Greece	46 452<br>(43 450 to 49 616)	488<br>(454 to 525)	-51.1<br>(-54.5 to -47.4)	24 437<br>(17 392 to 33 214)	165<br>(116 to 224)	-10.1<br>(-12.0 to -8.2)	70 890<br>(63 146 to 79 491)	653<br>(596 to 717)	-44.7<br>(-48.1 to -41.1)
Iceland	582<br>(538 to 628)	177<br>(162 to 192)	-69.5<br>(-72.6 to -65.9)	532<br>(378 to 720)	131<br>(92 to 177)	-5.8<br>(-8.0 to -3.6)	1 114<br>(948 to 1 302)	307<br>(266 to 356)	-57.2<br>(-61.6 to -52.6)
Ireland	7 423<br>(6 793 to 8 140)	160<br>(146 to 177)	-76.2<br>(-78.5 to -73.7)	7 409<br>(5 254 to 10 019)	128<br>(90 to 173)	-7.2<br>(-9.3 to -5.1)	14 832<br>(12 585 to 17 501)	288<br>(246 to 337)	-64.5<br>(-68.5 to -60.6)
Israel	22 677<br>(21 143 to 24 440)	255<br>(237 to 275)	-49.5<br>(-53.5 to -45.3)	11 844<br>(8 372 to 16 067)	124<br>(87 to 168)	-6.2<br>(-8.3 to -4.1)	34 521<br>(30 884 to 39 035)	378<br>(340 to 427)	-40.5<br>(-44.6 to -36.4)
Italy	158 685<br>(147 495 to 170 172)	264<br>(246 to 284)	-67.1<br>(-69.5 to -64.5)	116 181<br>(82 801 to 156 972)	131<br>(93 to 178)	-21.3<br>(-22.9 to -19.6)	274 866<br>(241 324 to 316 404)	396<br>(354 to 447)	-59.3<br>(-62.1 to -56.2)
Luxembourg	1 467<br>(1 308 to 1 637)	253<br>(227 to 282)	-74.9<br>(-77.8 to -71.8)	1 177<br>(833 to 1 588)	156<br>(110 to 210)	-14.8<br>(-17.2 to -12.6)	2 643<br>(2 262 to 3 094)	409<br>(355 to 468)	-65.7<br>(-69.3 to -61.8)
Malta	686<br>(634 to 748)	170<br>(157 to 186)	-53.3<br>(-58.1 to -47.7)	681<br>(485 to 921)	116<br>(82 to 157)	14.8<br>(12.5 to 17.2)	1 367<br>(1 162 to 1 607)	286<br>(249 to 328)	-38.5<br>(-44.3 to -32.5)
Netherlands	27 490<br>(25 783 to 29 467)	161<br>(150 to 174)	-68.8<br>(-71.1 to -66.1)	24 765<br>(17 672 to 33 364)	110<br>(78 to 149)	-15.0<br>(-17.3 to -12.6)	52 255<br>(44 586 to 61 052)	272<br>(236 to 310)	-58.0<br>(-61.6 to -54.6)
Norway	8 290<br>(7 990 to 8 619)	164<br>(157 to 171)	-69.6<br>(-71.0 to -67.7)	8 859<br>(6 328 to 11 872)	132<br>(94 to 177)	-0.3<br>(-2.3 to 1.8)	17 149<br>(14 647 to 20 149)	296<br>(259 to 343)	-55.9<br>(-59.6 to -52.0)
Portugal	31 716<br>(29 181 to 34 370)	290<br>(266 to 316)	-82.4<br>(-84.0 to -80.7)	18 545<br>(13 137 to 25 090)	121<br>(86 to 164)	-40.3<br>(-42.2 to -38.4)	50 261<br>(44 310 to 57 039)	411<br>(367 to 457)	-77.8<br>(-79.9 to -76.0)
Spain	87 642<br>(81 548 to 94 215)	195<br>(182 to 211)	-81.9<br>(-83.3 to -80.3)	88 107<br>(62 455 to 119 208)	139<br>(98 to 188)	-22.0<br>(-24.2 to -19.9)	175 749<br>(149 104 to 205 750)	334<br>(292 to 380)	-73.4<br>(-75.9 to -70.6)
Sweden	14 171<br>(13 062 to 15 302)	147<br>(134 to 159)	-70.6<br>(-72.9 to -68.2)	16 377<br>(11 745 to 21 921)	125<br>(89 to 168)	-9.2<br>(-11.2 to -7.2)	30 548<br>(25 745 to 36 035)	272<br>(233 to 316)	-57.3<br>(-61.2 to -53.3)
Switzerland	11 763<br>(10 831 to 12 907)	142<br>(131 to 154)	-80.1<br>(-81.8 to -78.0)	15 366<br>(10 896 to 20 812)	131<br>(92 to 177)	-18.3<br>(-19.8 to -16.6)	27 128<br>(22 469 to 32 566)	273<br>(232 to 321)	-68.7<br>(-72.3 to -64.9)

Location	YLLs (95% UI)			YLDs (95% UI)			DALYs (95% UI)		
	2017 counts	2017 age-standardised rates per 100,000	Percentage change in age-standardised rates between 1990 and 2017	2017 counts	2017 age-standardised rates per 100,000	Percentage change in age-standardised rates between 1990 and 2017	2017 counts	2017 age-standardised rates per 100,000	Percentage change in age-standardised rates between 1990 and 2017
United Kingdom	107 474 (104 867 to 110 771)	170 (166 to 176)	-66.3 (-67.2 to -65.1)	90 613 (64 656 to 122 645)	106 (75 to 144)	-8.0 (-9.2 to -6.7)	198 087 (171 611 to 230 317)	277 (245 to 314)	-55.4 (-58.3 to -52.3)
**Latin America and Caribbean**	**5 342 067 (5 130 650 to 5 532 635)**	**889 (855 to 920)**	**-37.3 (-39.6 to -34.3)**	**421 977 (305 404 to 562 074)**	**71 (51 to 94)**	**18.9 (15.6 to 22.7)**	**5 764 044 (5 527 287 to 5 975 477)**	**960 (921 to 995)**	**-35.0 (-37.3 to -32.1)**
**Andean Latin America**	**526 055 (475 228 to 576 064)**	**850 (770 to 930)**	**-33.8 (-40.8 to -25.6)**	**30 811 (22 050 to 41 157)**	**53 (38 to 71)**	**-3.6 (-7.8 to 0.9)**	**556 865 (504 790 to 608 324)**	**904 (819 to 987)**	**-32.6 (-39.4 to -24.7)**
Bolivia	97 827 (66 587 to 123 138)	864 (605 to 1 078)	-59.3 (-71.9 to -46.5)	5 122 (3 698 to 6 831)	52 (37 to 68)	-18.8 (-22.2 to -15.1)	102 949 (71 705 to 127 573)	916 (650 to 1 127)	-58.2 (-70.7 to -45.4)
Ecuador	217 207 (195 209 to 242 896)	1 276 (1 148 to 1 427)	-7.6 (-17.6 to 3.9)	10 311 (7 369 to 13 856)	65 (47 to 88)	4.8 (-0.3 to 10.4)	227 518 (205 200 to 253 126)	1 341 (1 210 to 1 490)	-7.1 (-16.8 to 4.0)
Peru	211 020 (177 718 to 246 077)	631 (534 to 735)	-36.2 (-46.6 to -23.1)	15 378 (10 931 to 20 620)	48 (34 to 65)	-4.0 (-8.7 to 0.8)	226 398 (192 770 to 261 109)	679 (580 to 783)	-34.6 (-44.8 to -22.1)
**Caribbean**	**520 168 (435 356 to 600 522)**	**1 120 (936 to 1 289)**	**-25.9 (-34.2 to -15.4)**	**26 379 (18 766 to 35 493)**	**53 (38 to 72)**	**5.0 (2.8 to 7.3)**	**546 547 (462 186 to 627 664)**	**1 174 (989 to 1 343)**	**-24.9 (-32.8 to -14.8)**
Antigua and Barbuda	312 (278 to 348)	342 (304 to 382)	-34.4 (-42.8 to -25.2)	41 (29 to 56)	41 (29 to 55)	9.8 (5.7 to 13.5)	354 (318 to 391)	382 (342 to 425)	-31.5 (-39.7 to -22.5)
The Bahamas	2 942 (2 635 to 3 296)	744 (667 to 830)	-27.1 (-36.2 to -16.6)	202 (143 to 275)	50 (35 to 68)	-3.5 (-6.2 to -0.9)	3 144 (2 828 to 3 487)	794 (713 to 880)	-26.0 (-34.8 to -16.0)
Barbados	1 255 (1 130 to 1 393)	424 (381 to 469)	-33.6 (-41.0 to -25.1)	172 (122 to 235)	45 (32 to 61)	13.5 (9.7 to 17.3)	1 427 (1 292 to 1 575)	469 (424 to 517)	-30.8 (-38.0 to -22.8)
Belize	3 768 (3 405 to 4 082)	923 (835 to 999)	-22.6 (-35.6 to -10.4)	188 (135 to 254)	56 (40 to 75)	12.0 (8.4 to 15.5)	3 956 (3 599 to 4 291)	979 (890 to 1 059)	-21.2 (-33.9 to -9.4)
Bermuda	241 (214 to 274)	363 (317 to 415)	-61.6 (-67.1 to -55.5)	59 (42 to 80)	63 (45 to 86)	-2.9 (-6.8 to 1.2)	300 (267 to 336)	426 (376 to 481)	-57.8 (-63.0 to -52.1)
Cuba	43 395 (38 485 to 49 497)	368 (328 to 416)	-65.7 (-69.5 to -60.9)	5 744 (4 058 to 7 867)	38 (27 to 52)	-23.8 (-26.2 to -21.4)	49 139 (43 890 to 55 382)	406 (364 to 456)	-63.8 (-67.6 to -59.3)
Dominica	549 (496 to 607)	801 (721 to 888)	-10.7 (-20.7 to 0.5)	39 (28 to 52)	49 (35 to 65)	26.7 (22.9 to 30.6)	588 (533 to 648)	850 (768 to 937)	-9.2 (-18.8 to 1.8)
Dominican Republic	151 993 (128 801 to 176 045)	1 399 (1 186 to 1 620)	26.7 (4.6 to 52.8)	8 012 (5 696 to 10 871)	79 (56 to 108)	44.3 (37.3 to 52.1)	160 005 (136 735 to 184 366)	1 478 (1 266 to 1 704)	27.5 (6.6 to 52.2)
Grenada	502 (455 to 549)	444 (401 to 488)	-43.4 (-49.8 to -36.2)	51 (36 to 69)	40 (28 to 54)	6.7 (2.6 to 10.3)	552 (504 to 602)	484 (440 to 528)	-41.1 (-47.5 to -34.0)
Guyana	5 852 (4 985 to 6 832)	763 (652 to 889)	-16.4 (-29.0 to -2.1)	310 (222 to 415)	44 (31 to 59)	28.0 (23.8 to 32.3)	6 162 (5 338 to 7 138)	807 (698 to 933)	-14.8 (-27.2 to -1.0)
Haiti	244 052 (168 317 to 322 144)	2 035 (1 381 to 2 694)	-45.5 (-56.7 to -29.3)	5 091 (3 701 to 6 724)	53 (38 to 69)	-13.7 (-16.5 to -10.9)	249 143 (174 196 to 326 445)	2 088 (1 435 to 2 748)	-45.0 (-56.0 to -29.1)
Jamaica	12 520 (9 933 to 15 070)	424 (336 to 509)	91.4 (48.6 to 134.4)	1 324 (940 to 1 788)	45 (32 to 61)	71.6 (64.9 to 78.5)	13 844 (11 160 to 16 451)	469 (379 to 555)	89.3 (51.0 to 127.4)
Puerto Rico	17 698 (16 174 to 19 405)	482 (440 to 530)	-48.4 (-53.6 to -43.0)	2 748 (1 944 to 3 727)	55 (39 to 75)	5.0 (2.1 to 8.0)	20 445 (18 743 to 22 369)	538 (493 to 589)	-45.6 (-50.5 to -40.3)
Saint Lucia	1 133 (1 003 to 1 265)	606 (539 to 677)	-39.7 (-47.5 to -30.9)	97 (69 to 130)	48 (34 to 64)	-1.4 (-4.7 to 1.9)	1 230 (1 097 to 1 360)	653 (583 to 723)	-37.9 (-45.4 to -29.5)
Saint Vincent and the Grenadines	536 (484 to 592)	460 (415 to 507)	-16.6 (-26.8 to -5.3)	52 (37 to 70)	41 (29 to 55)	35.7 (31.9 to 40.0)	588 (537 to 645)	501 (456 to 548)	-13.9 (-23.7 to -3.1)
Suriname	4 729 (4 139 to 5 345)	814 (714 to 919)	-30.8 (-40.9 to -19.4)	346 (247 to 463)	58 (41 to 77)	7.7 (4.0 to 11.3)	5 075 (4 474 to 5 716)	871 (769 to 979)	-29.1 (-38.8 to -18.2)
Trinidad and Tobago	9 451 (7 662 to 11 532)	669 (547 to 810)	-13.0 (-29.4 to 6.7)	885 (631 to 1 199)	53 (38 to 72)	18.2 (14.5 to 21.9)	10 336 (8 546 to 12 459)	722 (602 to 868)	-11.3 (-26.9 to 7.4)
Virgin Islands	533 (467 to 608)	467 (416 to 533)	-41.1 (-50.0 to -30.0)	71 (50 to 97)	50 (35 to 68)	5.0 (0.9 to 9.4)	604 (535 to 685)	517 (464 to 586)	-38.4 (-46.8 to -28.0)
**Central Latin America**	**2 029 275 (1 913 814 to 2 129 603)**	**773 (730 to 811)**	**-39.0 (-43.0 to -35.9)**	**161 673 (116 325 to 215 968)**	**65 (47 to 87)**	**4.5 (1.5 to 7.8)**	**2 190 948 (2 065 767 to 2 304 297)**	**838 (791 to 882)**	**-37.0 (-40.9 to -33.9)**
Colombia	350 287 (306 836 to 396 762)	677 (593 to 766)	-41.0 (-48.7 to -32.9)	23 680 (16 916 to 32 102)	44 (32 to 60)	-20.0 (-23.2 to -16.7)	373 967 (328 827 to 421 733)	721 (635 to 813)	-40.0 (-47.4 to -32.3)
Costa Rica	33 941 (30 528 to 37 273)	685 (618 to 752)	-18.8 (-26.5 to -9.9)	2 642 (1 873 to 3 583)	53 (37 to 72)	6.0 (2.2 to 9.5)	36 583 (33 135 to 40 213)	738 (672 to 812)	-17.5 (-24.8 to -8.8)
El Salvador	53 702 (44 116 to 66 477)	871 (717 to 1 072)	-41.0 (-52.3 to -26.2)	2 608 (1 854 to 3 526)	45 (32 to 60)	-15.3 (-19.6 to -11.1)	56 311 (46 509 to 68 774)	915 (758 to 1 111)	-40.1 (-50.8 to -25.8)
Guatemala	139 898 (121 714 to 160 967)	806 (706 to 925)	-12.4 (-25.0 to 1.1)	5 714 (4 142 to 7 717)	43 (31 to 58)	6.7 (2.6 to 10.8)	145 612 (127 413 to 167 044)	849 (746 to 968)	-11.6 (-23.6 to 1.3)
Honduras	61 489 (47 296 to 76 060)	675 (515 to 830)	-42.7 (-55.9 to -25.5)	3 753 (2 714 to 5 001)	51 (37 to 68)	15.2 (10.7 to 19.5)	65 242 (51 722 to 80 110)	727 (575 to 886)	-40.6 (-53.5 to -24.1)
Mexico	976 016 (944 208 to 1 009 136)	754 (730 to 780)	-42.4 (-44.9 to -40.2)	99 919 (72 369 to 133 354)	80 (58 to 107)	20.7 (16.7 to 24.7)	1 075 935 (1 033 221 to 1 125 009)	834 (801 to 873)	-39.3 (-42.0 to -37.0)
Nicaragua	32 186 (27 313 to 38 084)	496 (422 to 586)	-54.9 (-61.7 to -47.0)	1 898 (1 359 to 2 539)	35 (25 to 47)	-26.1 (-29.2 to -23.0)	34 083 (29 297 to 39 776)	531 (457 to 617)	-53.7 (-60.3 to -46.2)
Panama	23 781 (21 716 to 25 980)	601 (550 to 658)	-41.9 (-47.5 to -35.7)	1 974 (1 401 to 2 679)	49 (35 to 67)	-10.8 (-14.6 to -7.6)	25 755 (23 433 to 28 055)	651 (593 to 709)	-40.3 (-45.9 to -34.4)
Venezuela	357 975 (298 694 to 431 649)	1 109 (927 to 1 333)	-28.1 (-40.2 to -14.1)	19 486 (13 841 to 26 374)	64 (45 to 86)	-14.5 (-18.0 to -11.2)	377 461 (316 999 to 448 718)	1 172 (986 to 1 391)	-27.5 (-39.1 to -14.2)
**Tropical Latin America**	**2 266 569 (2 166 677 to 2 350 324)**	**983 (942 to 1 019)**	**-38.1 (-40.9 to -34.7)**	**203 116 (146 505 to 271 450)**	**84 (61 to 113)**	**40.1 (34.4 to 46.1)**	**2 469 684 (2 356 260 to 2 573 388)**	**1 068 (1 020 to 1 113)**	**-35.3 (-38.2 to -31.5)**
Brazil	2 191 726 (2 095 556 to 2 271 108)	982 (939 to 1 017)	-39.0 (-41.8 to -35.5)	198 635 (143 352 to 265 657)	85 (61 to 113)	40.0 (34.2 to 46.1)	2 390 361 (2 278 881 to 2 491 383)	1 066 (1 018 to 1 110)	-36.2 (-39.1 to -32.4)
Paraguay	74 842 (60 814 to 91 151)	1 049 (854 to 1 280)	28.6 (2.5 to 61.0)	4 481 (3 198 to 5 998)	71 (51 to 95)	48.0 (42.5 to 53.7)	79 323 (65 439 to 96 006)	1 120 (927 to 1 356)	29.7 (5.2 to 60.3)
**North Africa and Middle East**	**6 854 317 (6 120 320 to 7 888 231)**	**1 117 (993 to 1 287)**	**-47.8 (-52.7 to -39.2)**	**545 368 (391 372 to 732 789)**	**100 (72 to 134)**	**-25.4 (-28.0 to -22.5)**	**7 399 686 (6 614 065 to 8 484 686)**	**1 217 (1 085 to 1 398)**	**-46.5 (-51.2 to -38.4)**
**North Africa and Middle East**	**6 854 317 (6 120 320 to 7 888 231)**	**1 117 (993 to 1 287)**	**-47.8 (-52.7 to -39.2)**	**545 368 (391 372 to 732 789)**	**100 (72 to 134)**	**-25.4 (-28.0 to -22.5)**	**7 399 686 (6 614 065 to 8 484 686)**	**1 217 (1 085 to 1 398)**	**-46.5 (-51.2 to -38.4)**
Afghanistan	512 629 (408 661 to 630 361)	1 654 (1 308 to 2 044)	-38.5 (-57.8 to 114.9)	17 952 (13 146 to 23 624)	88 (65 to 115)	-17.3 (-18.9 to -15.8)	530 582 (424 959 to 649 158)	1 741 (1 400 to 2 127)	-37.7 (-56.6 to 99.1)
Algeria	353 710 (286 415 to 554 862)	852 (690 to 1 349)	-53.4 (-59.8 to -44.9)	42 847 (30 744 to 57 512)	110 (79 to 148)	-34.2 (-37.0 to -31.4)	396 558 (324 692 to 600 532)	963 (790 to 1 460)	-51.8 (-57.8 to -44.1)
Bahrain	6 274 (5 515 to 7 124)	435 (387 to 488)	-59.7 (-64.7 to -53.1)	1 481 (1 054 to 1 993)	90 (64 to 121)	-40.2 (-42.7 to -37.8)	7 755 (6 899 to 8 786)	525 (471 to 587)	-57.3 (-61.9 to -51.4)
Egypt	1 394 257 (1 069 676 to 1 705 353)	1 450 (1 078 to 1 796)	-46.9 (-55.7 to -36.0)	91 138 (65 455 to 121 801)	113 (82 to 151)	-24.8 (-28.3 to -20.9)	1 485 396 (1 157 770 to 1 797 040)	1 563 (1 189 to 1 915)	-45.8 (-54.0 to -35.4)
Iran	1 048 280 (1 022 382 to 1 120 805)	1 261 (1 229 to 1 358)	-54.7 (-60.2 to -50.9)	89 650 (63 929 to 120 908)	106 (76 to 143)	-36.7 (-39.4 to -33.9)	1 137 930 (1 097 622 to 1 207 599)	1 368 (1 318 to 1 463)	-53.7 (-59.1 to -49.8)
Iraq	220 334 (197 196 to 249 504)	487 (441 to 545)	-62.8 (-69.4 to -47.2)	22 071 (15 903 to 29 419)	66 (47 to 87)	-38.6 (-40.2 to -37.1)	242 404 (219 798 to 272 385)	553 (504 to 616)	-61.0 (-67.4 to -46.3)
Jordan	62 672 (55 183 to 71 509)	571 (507 to 647)	-56.7 (-63.4 to -47.4)	5 995 (4 251 to 8 084)	68 (48 to 92)	-39.6 (-41.8 to -37.4)	68 668 (60 876 to 77 619)	640 (568 to 719)	-55.4 (-61.7 to -46.7)
Kuwait	26 056 (23 609 to 28 310)	614 (560 to 664)	-52.4 (-56.7 to -47.8)	4 890 (3 454 to 6 561)	114 (82 to 154)	-30.7 (-32.3 to -28.9)	30 946 (28 127 to 33 684)	728 (663 to 794)	-50.0 (-53.9 to -45.7)
Lebanon	28 968 (19 964 to 35 638)	326 (225 to 400)	-48.5 (-66.8 to -33.8)	6 530 (4 610 to 8 817)	85 (60 to 115)	-3.4 (-7.5 to 0.5)	35 497 (26 408 to 42 882)	411 (309 to 494)	-43.0 (-59.9 to -29.5)
Libya	86 272 (45 129 to 128 825)	1 186 (625 to 1 778)	-29.9 (-56.1 to -9.4)	7 716 (5 520 to 10 314)	121 (86 to 162)	-27.7 (-30.6 to -24.5)	93 987 (52 875 to 137 303)	1 306 (746 to 1 908)	-29.7 (-53.2 to -10.9)
Morocco	360 805 (270 428 to 570 594)	994 (747 to 1 576)	-48.1 (-59.6 to -34.5)	35 353 (25 376 to 47 344)	100 (71 to 133)	-23.6 (-26.4 to -20.3)	396 158 (304 556 to 604 021)	1 093 (841 to 1 672)	-46.5 (-57.2 to -33.7)
Palestine	20 407 (17 605 to 23 941)	405 (355 to 465)	-55.6 (-63.8 to -44.1)	2 104 (1 508 to 2 817)	57 (41 to 76)	-13.8 (-16.0 to -11.6)	22 510 (19 657 to 26 050)	462 (408 to 525)	-52.8 (-60.7 to -41.5)
Oman	101 538 (82 585 to 121 317)	2 058 (1 685 to 2 434)	-55.6 (-65.3 to -42.8)	7 017 (4 967 to 9 471)	168 (119 to 227)	-38.4 (-41.0 to -35.8)	108 555 (89 480 to 128 219)	2 226 (1 853 to 2 606)	-54.6 (-63.9 to -42.4)
Qatar	29 261 (23 711 to 35 821)	1 028 (842 to 1 247)	-40.9 (-54.0 to -24.5)	4 052 (2 872 to 5 455)	148 (105 to 198)	-25.7 (-28.2 to -23.1)	33 312 (27 059 to 40 040)	1 176 (977 to 1 400)	-39.3 (-51.5 to -24.6)
Saudi Arabia	592 079 (416 802 to 733 013)	1 518 (1 086 to 1 848)	-32.6 (-56.4 to -9.7)	49 572 (35 022 to 66 865)	156 (111 to 211)	-26.9 (-30.4 to -23.5)	641 652 (468 435 to 781 641)	1 674 (1 240 to 2 003)	-32.1 (-54.0 to -11.6)
Sudan	646 392 (493 977 to 915 010)	1 523 (1 162 to 2 254)	-59.5 (-69.8 to -38.3)	31 009 (22 738 to 40 606)	112 (82 to 147)	-24.3 (-26.5 to -22.3)	677 401 (523 043 to 945 518)	1 635 (1 271 to 2 380)	-58.2 (-68.0 to -37.4)
Syria	80 124 (64 649 to 96 767)	458 (368 to 552)	-52.3 (-63.5 to -37.9)	9 844 (7 038 to 13 237)	63 (45 to 85)	-23.4 (-26.5 to -20.2)	89 968 (73 930 to 106 694)	521 (430 to 614)	-50.1 (-60.7 to -36.6)
Tunisia	147 868 (118 455 to 182 477)	1 222 (983 to 1 499)	-44.7 (-58.2 to -29.5)	11 256 (8 030 to 15 162)	89 (64 to 120)	-15.5 (-19.1 to -11.8)	159 124 (129 113 to 193 430)	1 312 (1 065 to 1 589)	-43.4 (-56.6 to -28.9)
Turkey	409 862 (368 378 to 457 221)	506 (454 to 565)	-41.5 (-50.6 to -30.6)	61 122 (43 578 to 82 824)	69 (49 to 94)	-9.3 (-14.7 to -3.5)	470 984 (424 967 to 519 859)	576 (517 to 636)	-38.9 (-47.8 to -28.6)
United Arab Emirates	169 076 (129 504 to 212 174)	1 838 (1 463 to 2 254)	-20.4 (-44.9 to 8.7)	18 754 (13 397 to 25 192)	173 (123 to 231)	-27.1 (-30.2 to -24.1)	187 830 (148 382 to 231 627)	2 011 (1 631 to 2 425)	-21.0 (-43.5 to 4.9)
Yemen	551 053 (424 309 to 772 177)	1 827 (1 379 to 2 616)	-49.3 (-63.6 to 15.7)	24 506 (17 937 to 32 505)	119 (87 to 157)	-29.0 (-31.3 to -26.7)	575 560 (446 470 to 797 337)	1 946 (1 488 to 2 732)	-48.4 (-62.3 to 12.0)
**South Asia**	**13 574 498 (12 046 498 to 14 712 207)**	**762 (675 to 824)**	**-9.6 (-18.8 to 0.9)**	**1 513 080 (1 097 334 to 2 024 113)**	**92 (67 to 123)**	**32.2 (28.9 to 35.9)**	**15 087 578 (13 476 788 to 16 265 578)**	**854 (762 to 919)**	**-6.4 (-14.9 to 3.6)**
**South Asia**	**13 574 498 (12 046 498 to 14 712 207)**	**762 (675 to 824)**	**-9.6 (-18.8 to 0.9)**	**1 513 080 (1 097 334 to 2 024 113)**	**92 (67 to 123)**	**32.2 (28.9 to 35.9)**	**15 087 578 (13 476 788 to 16 265 578)**	**854 (762 to 919)**	**-6.4 (-14.9 to 3.6)**
Bangladesh	552 271 (423 294 to 649 550)	350 (270 to 410)	-0.4 (-22.4 to 34.7)	88 360 (63 357 to 117 782)	60 (43 to 80)	37.7 (31.3 to 44.8)	640 631 (517 124 to 742 357)	410 (334 to 472)	3.8 (-16.4 to 35.2)
Bhutan	3 598 (2 551 to 4 386)	359 (264 to 435)	-61.0 (-71.7 to -47.5)	665 (478 to 889)	75 (54 to 100)	-15.4 (-19.5 to -11.1)	4 263 (3 137 to 5 069)	434 (334 to 513)	-57.0 (-67.5 to -44.3)
India	9 861 905 (9 299 930 to 10 395 839)	712 (669 to 751)	-17.4 (-24.5 to -9.2)	1 197 464 (870 274 to 1 601 533)	92 (67 to 123)	29.8 (26.5 to 33.5)	11 059 369 (10 340 644 to 11 722 367)	804 (751 to 853)	-13.8 (-20.7 to -5.7)
Nepal	296 686 (173 127 to 454 931)	1 030 (600 to 1 569)	-5.5 (-36.2 to 39.9)	23 707 (17 105 to 31 644)	90 (65 to 120)	22.0 (17.4 to 26.0)	320 393 (197 878 to 478 580)	1 120 (690 to 1 658)	-3.7 (-32.5 to 39.0)
Pakistan	2 860 037 (1 775 592 to 3 723 256)	1 338 (808 to 1 744)	20.9 (-3.8 to 55.3)	202 885 (146 760 to 272 392)	119 (86 to 160)	48.0 (43.6 to 52.7)	3 062 923 (1 957 133 to 3 916 726)	1 457 (928 to 1 867)	22.8 (-0.4 to 54.8)
**Southeast Asia, East Asia, and Oceania**	**16 774 732 (16 041 143 to 17 411 642)**	**740 (710 to 768)**	**-35.5 (-41.1 to -31.3)**	**3 971 033 (2 835 071 to 5 346 287)**	**151 (108 to 204)**	**58.7 (52.1 to 65.7)**	**20 745 766 (19 364 326 to 22 212 060)**	**891 (836 to 948)**	**-28.3 (-34.2 to -23.4)**
**East Asia**	**11 197 430 (10 631 229 to 11 709 813)**	**704 (672 to 732)**	**-33.3 (-40.2 to -27.9)**	**3 008 883 (2 163 948 to 4 052 098)**	**155 (111 to 209)**	**69.1 (61.3 to 77.5)**	**14 206 313 (13 132 075 to 15 351 722)**	**858 (802 to 919)**	**-25.1 (-31.9 to -19.1)**
China	10 603 693 (10 089 242 to 11 068 342)	699 (669 to 728)	-33.3 (-40.4 to -27.7)	2 863 875 (2 060 723 to 3 858 892)	155 (111 to 209)	70.2 (62.1 to 79.1)	13 467 568 (12 456 217 to 14 568 835)	854 (798 to 914)	-25.0 (-32.5 to -18.8)
North Korea	275 735 (183 449 to 420 361)	1 023 (699 to 1 532)	37.0 (-3.1 to 83.0)	46 651 (33 617 to 62 289)	150 (108 to 201)	101.0 (95.8 to 106.1)	322 386 (228 500 to 466 947)	1 173 (844 to 1 672)	42.9 (5.8 to 84.9)
Taiwan (Province of China)	137 619 (129 512 to 147 237)	540 (509 to 578)	-69.3 (-71.2 to -67.1)	49 886 (35 549 to 67 612)	153 (109 to 208)	10.7 (6.2 to 14.9)	187 504 (170 347 to 207 619)	694 (636 to 758)	-63.5 (-66.0 to -60.6)
**Oceania**	**180 258 (146 861 to 217 705)**	**1 441 (1 170 to 1 734)**	**-20.1 (-36.0 to -2.3)**	**14 204 (10 373 to 18 772)**	**142 (104 to 189)**	**60.7 (57.3 to 63.8)**	**194 462 (160 417 to 232 796)**	**1 583 (1 314 to 1 896)**	**-16.4 (-31.6 to 1.4)**
American Samoa	211 (187 to 234)	390 (348 to 430)	-29.1 (-42.1 to -16.8)	55 (40 to 74)	108 (78 to 145)	43.7 (39.3 to 48.3)	266 (239 to 297)	498 (447 to 556)	-20.3 (-33.2 to -8.3)
Federated States of Micronesia	848 (553 to 1 094)	814 (524 to 1 046)	-19.9 (-47.7 to 7.9)	124 (91 to 165)	135 (99 to 180)	64.0 (58.8 to 69.4)	972 (679 to 1 231)	950 (662 to 1 199)	-13.6 (-39.9 to 13.2)
Fiji	4 334 (3 751 to 5 015)	474 (412 to 549)	-20.7 (-37.4 to -1.6)	991 (719 to 1 320)	112 (81 to 149)	72.6 (67.4 to 77.4)	5 325 (4 656 to 6 049)	586 (514 to 665)	-11.6 (-28.4 to 6.8)
Guam	1 039 (928 to 1 153)	624 (555 to 695)	-14.0 (-27.3 to -0.2)	264 (190 to 356)	147 (106 to 199)	56.3 (51.8 to 60.7)	1 303 (1 164 to 1 445)	772 (688 to 857)	-5.9 (-18.8 to 7.0)
Kiribati	679 (520 to 832)	559 (433 to 683)	-9.6 (-31.6 to 17.1)	98 (71 to 129)	99 (72 to 131)	92.6 (86.3 to 99.4)	777 (623 to 938)	658 (530 to 787)	-1.7 (-23.0 to 24.0)

Location	YLLs (95% UI)			YLDs (95% UI)			DALYs (95% UI)		
	2017 counts	2017 age-standardised rates per 100,000	Percentage change in age-standardised rates between 1990 and 2017	2017 counts	2017 age-standardised rates per 100,000	Percentage change in age-standardised rates between 1990 and 2017	2017 counts	2017 age-standardised rates per 100,000	Percentage change in age-standardised rates between 1990 and 2017
Marshall Islands	643 (496 to 781)	1 136 (884 to 1 375)	-14.7 (-33.3 to 6.4)	67 (49 to 90)	141 (102 to 187)	77.9 (72.9 to 83.4)	710 (565 to 852)	1 277 (1 024 to 1 522)	-9.6 (-27.4 to 10.7)
Northern Mariana Islands	210 (181 to 238)	468 (405 to 532)	-35.7 (-51.3 to -16.7)	65 (47 to 88)	119 (85 to 161)	15.1 (11.7 to 18.5)	275 (241 to 309)	588 (514 to 658)	-29.3 (-44.7 to -12.3)
Papua New Guinea	151 587 (119 994 to 186 373)	1 666 (1 326 to 2 047)	-27.8 (-43.8 to -9.5)	10 443 (7 617 to 13 768)	148 (109 to 196)	53.9 (50.0 to 57.6)	162 030 (130 355 to 197 795)	1 814 (1 468 to 2 205)	-24.5 (-39.9 to -6.0)
Samoa	887 (681 to 1 230)	467 (358 to 654)	-23.6 (-42.5 to -2.7)	210 (152 to 280)	130 (94 to 174)	70.9 (66.2 to 75.5)	1 097 (874 to 1 456)	597 (475 to 794)	-13.2 (-32.2 to 6.8)
Solomon Islands	6 476 (5 041 to 8 202)	1 036 (803 to 1 318)	-17.7 (-36.9 to 6.1)	659 (482 to 866)	137 (100 to 179)	71.7 (67.8 to 75.6)	7 136 (5 619 to 8 884)	1 173 (929 to 1 460)	-12.4 (-31.8 to 10.1)
Tonga	575 (466 to 669)	562 (461 to 654)	-4.0 (-30.9 to 22.4)	98 (71 to 130)	109 (78 to 145)	67.1 (61.5 to 72.6)	673 (562 to 774)	671 (562 to 775)	3.1 (-22.2 to 27.1)
Vanuatu	2 836 (1 954 to 3 958)	994 (683 to 1 394)	-5.2 (-33.5 to 34.9)	347 (255 to 459)	156 (115 to 206)	84.1 (79.9 to 88.1)	3 183 (2 290 to 4 301)	1 150 (842 to 1 546)	1.5 (-25.0 to 39.9)
**Southeast Asia**	**5 397 044 (5 065 758 to 5 724 664)**	**793 (746 to 840)**	**-44.5 (-48.6 to -40.4)**	**947 946 (685 084 to 1 262 382)**	**140 (101 to 187)**	**30.7 (26.6 to 35.1)**	**6 344 990 (5 922 383 to 6 775 553)**	**933 (872 to 997)**	**-39.3 (-43.5 to -34.9)**
Cambodia	189 636 (147 941 to 246 258)	1 177 (927 to 1 521)	-44.4 (-56.1 to -26.4)	20 076 (14 577 to 26 553)	140 (102 to 185)	43.6 (38.8 to 48.7)	209 712 (169 566 to 267 770)	1 317 (1 069 to 1 674)	-40.5 (-51.9 to -22.7)
Indonesia	1 741 795 (1 609 611 to 1 877 236)	666 (614 to 716)	-56.1 (-60.6 to -50.9)	380 609 (277 973 to 500 869)	146 (106 to 192)	25.0 (21.0 to 28.8)	2 122 404 (1 949 446 to 2 298 442)	812 (746 to 878)	-50.4 (-55.2 to -44.7)
Laos	91 533 (67 617 to 112 384)	1 270 (945 to 1 566)	-48.3 (-61.7 to -30.2)	9 663 (7 038 to 12 751)	163 (119 to 216)	35.6 (30.5 to 40.3)	101 196 (77 452 to 123 309)	1 433 (1 099 to 1 738)	-44.4 (-57.4 to -26.0)
Malaysia	318 204 (279 583 to 356 887)	980 (864 to 1 097)	-26.7 (-41.9 to -13.0)	54 667 (38 905 to 73 564)	181 (129 to 244)	37.6 (30.8 to 44.5)	372 872 (329 282 to 417 086)	1 161 (1 028 to 1 294)	-21.0 (-35.8 to -8.3)
Maldives	1 473 (1 093 to 2 696)	320 (241 to 577)	-69.4 (-74.7 to -57.4)	484 (344 to 651)	110 (78 to 148)	12.1 (6.0 to 18.7)	1 957 (1 544 to 3 188)	430 (342 to 690)	-62.5 (-67.8 to -52.5)
Mauritius	7 242 (6 572 to 7 954)	537 (489 to 591)	-17.5 (-26.3 to -7.9)	1 864 (1 337 to 2 504)	119 (85 to 159)	69.8 (63.8 to 76.1)	9 105 (8 260 to 10 032)	656 (596 to 722)	-9.0 (-17.3 to -0.4)
Myanmar	532 120 (440 388 to 644 129)	967 (801 to 1 167)	-50.2 (-60.7 to -36.2)	66 008 (47 763 to 87 499)	126 (91 to 166)	32.3 (27.3 to 37.7)	598 128 (503 422 to 713 242)	1 093 (924 to 1 295)	-46.3 (-56.7 to -32.8)
Philippines	558 459 (480 801 to 643 064)	534 (463 to 614)	13.9 (-3.1 to 33.5)	84 891 (60 737 to 113 513)	93 (66 to 123)	75.0 (68.8 to 81.7)	643 350 (559 029 to 730 170)	627 (547 to 711)	20.1 (4.4 to 38.2)
Sri Lanka	101 980 (82 473 to 124 188)	451 (365 to 550)	-19.0 (-35.3 to -0.0)	25 584 (18 250 to 34 564)	106 (75 to 143)	95.0 (86.4 to 104.8)	127 564 (105 880 to 152 571)	557 (460 to 665)	-8.9 (-24.8 to 9.0)
Seychelles	606 (519 to 688)	567 (486 to 646)	18.7 (1.9 to 35.4)	143 (103 to 193)	125 (89 to 169)	73.0 (65.9 to 80.4)	749 (649 to 848)	693 (602 to 783)	25.8 (10.7 to 40.6)
Thailand	894 374 (785 651 to 1 002 113)	1 261 (1 110 to 1 406)	-40.6 (-51.5 to -30.0)	158 542 (112 432 to 213 026)	173 (122 to 233)	11.4 (6.2 to 17.2)	1 052 917 (930 586 to 1 174 922)	1 434 (1 269 to 1 598)	-37.0 (-47.4 to -26.5)
Timor-Leste	6 303 (3 259 to 10 632)	479 (256 to 821)	-38.4 (-66.2 to -17.9)	1 095 (799 to 1 446)	113 (82 to 149)	63.7 (57.1 to 71.1)	7 398 (4 319 to 11 672)	592 (366 to 928)	-30.1 (-55.1 to -11.0)
Vietnam	946 218 (775 240 to 1 078 257)	931 (769 to 1 059)	-27.3 (-43.1 to -10.2)	143 072 (102 142 to 191 935)	139 (99 to 186)	40.7 (33.6 to 47.5)	1 089 290 (918 886 to 1 225 869)	1 069 (912 to 1 205)	-22.5 (-37.4 to -6.6)
**Sub-Saharan Africa**	**8 754 769 (8 000 791 to 9 513 743)**	**905 (840 to 973)**	**-37.0 (-43.8 to -26.3)**	**780 603 (566 020 to 1 033 301)**	**112 (81 to 147)**	**-22.2 (-23.6 to -20.6)**	**9 535 372 (8 734 873 to 10 336 023)**	**1 017 (944 to 1 099)**	**-35.6 (-41.9 to -25.6)**
**Central sub-Saharan Africa**	**2 000 402 (1 585 674 to 2 403 747)**	**1 564 (1 302 to 1 834)**	**-34.1 (-45.9 to -9.9)**	**134 048 (97 246 to 176 617)**	**156 (114 to 204)**	**-21.0 (-22.7 to -19.3)**	**2 134 450 (1 714 422 to 2 550 412)**	**1 720 (1 448 to 1 999)**	**-33.1 (-44.1 to -11.0)**
Angola	418 203 (339 360 to 508 792)	1 433 (1 183 to 1 734)	-55.9 (-67.3 to -18.2)	33 590 (24 536 to 44 315)	180 (131 to 236)	-28.2 (-30.3 to -26.0)	451 793 (369 867 to 542 841)	1 613 (1 348 to 1 917)	-54.0 (-65.1 to -19.4)
Central African Republic	208 700 (117 092 to 282 178)	4 387 (2 472 to 5 777)	4.9 (-23.6 to 76.5)	5 812 (4 203 to 7 667)	160 (117 to 212)	-11.6 (-13.6 to -9.7)	214 512 (123 337 to 288 408)	4 547 (2 627 to 5 938)	4.2 (-22.9 to 71.6)
Congo (Brazzaville)	68 878 (52 308 to 87 389)	1 387 (1 068 to 1 746)	-46.8 (-59.5 to -28.0)	6 777 (4 917 to 8 967)	170 (124 to 224)	-22.1 (-24.0 to -20.1)	75 655 (58 926 to 94 255)	1 558 (1 227 to 1 923)	-44.9 (-57.0 to -27.6)
DR Congo	1 268 193 (939 349 to 1 614 419)	1 470 (1 130 to 1 836)	-25.7 (-41.5 to 9.7)	83 702 (60 822 to 110 644)	146 (107 to 191)	-19.2 (-21.4 to -17.1)	1 351 895 (1 021 304 to 1 699 895)	1 616 (1 271 to 1 974)	-25.2 (-39.9 to 6.4)
Equatorial Guinea	13 356 (9 177 to 18 797)	989 (672 to 1 414)	-71.9 (-81.2 to -57.8)	1 328 (955 to 1 772)	151 (109 to 200)	-26.4 (-30.5 to -22.1)	14 683 (10 455 to 20 299)	1 139 (824 to 1 577)	-69.4 (-78.7 to -55.8)
Gabon	23 071 (17 677 to 28 877)	1 345 (1 044 to 1 670)	-37.6 (-52.4 to -19.5)	2 841 (2 047 to 3 762)	198 (143 to 262)	-26.5 (-28.4 to -24.6)	25 912 (20 455 to 31 560)	1 543 (1 234 to 1 879)	-36.4 (-49.6 to -20.5)
**Eastern sub-Saharan Africa**	**2 695 814 (2 373 984 to 3 040 037)**	**796 (702 to 884)**	**-38.7 (-48.1 to -20.7)**	**290 273 (210 583 to 384 708)**	**115 (84 to 151)**	**-21.4 (-22.7 to -20.1)**	**2 986 087 (2 669 293 to 3 337 893)**	**911 (817 to 1 006)**	**-36.9 (-45.9 to -20.8)**
Burundi	116 993 (91 887 to 154 123)	1 278 (1 006 to 1 705)	-39.0 (-52.7 to -18.9)	9 970 (7 204 to 13 144)	143 (104 to 189)	-29.1 (-30.6 to -27.5)	126 962 (101 870 to 164 717)	1 421 (1 147 to 1 866)	-38.2 (-50.9 to -19.8)
Comoros	4 932 (4 062 to 6 152)	747 (617 to 918)	-48.7 (-59.3 to -34.6)	840 (610 to 1 104)	144 (105 to 189)	-32.9 (-34.6 to -31.2)	5 772 (4 878 to 6 988)	891 (760 to 1 064)	-46.6 (-56.6 to -34.3)
Djibouti	8 194 (5 921 to 11 964)	826 (594 to 1 182)	-33.9 (-53.2 to 5.1)	1 367 (993 to 1 825)	155 (112 to 205)	-23.2 (-25.4 to -21.1)	9 561 (7 194 to 13 279)	981 (737 to 1 328)	-32.4 (-49.3 to -0.4)
Eritrea	67 258 (47 597 to 85 484)	1 298 (926 to 1 631)	-41.6 (-57.9 to 4.1)	5 303 (3 843 to 7 011)	132 (96 to 172)	-20.6 (-22.5 to -18.9)	72 561 (53 128 to 90 859)	1 430 (1 054 to 1 765)	-40.2 (-55.8 to 1.0)
Ethiopia	499 860 (449 734 to 561 743)	558 (507 to 619)	-61.3 (-69.3 to -41.0)	54 677 (39 606 to 72 351)	85 (62 to 112)	-39.7 (-41.4 to -38.1)	554 537 (501 367 to 619 203)	642 (585 to 707)	-59.4 (-67.2 to -40.6)
Kenya	260 040 (236 654 to 301 931)	638 (582 to 738)	-12.5 (-36.4 to 2.6)	44 930 (32 791 to 59 369)	137 (100 to 181)	-3.4 (-4.7 to -2.2)	304 970 (278 188 to 347 294)	775 (707 to 876)	-11.0 (-32.5 to 1.5)
Madagascar	179 605 (142 982 to 223 515)	790 (643 to 973)	-43.6 (-54.8 to -27.4)	22 849 (16 506 to 30 163)	134 (97 to 177)	-28.5 (-30.5 to -26.7)	202 454 (166 670 to 246 804)	924 (772 to 1 113)	-41.8 (-52.2 to -27.6)
Malawi	111 972 (91 063 to 134 634)	738 (616 to 875)	-48.0 (-66.0 to 41.6)	9 819 (7 137 to 13 009)	86 (63 to 114)	-26.8 (-28.9 to -24.7)	121 790 (100 875 to 145 018)	825 (696 to 964)	-46.3 (-64.1 to 29.4)
Mozambique	261 562 (215 297 to 310 386)	1 059 (881 to 1 241)	-22.1 (-39.5 to -1.4)	21 320 (15 445 to 28 250)	117 (85 to 155)	-14.1 (-17.6 to -10.7)	282 881 (237 162 to 331 690)	1 176 (991 to 1 371)	-21.4 (-37.4 to -2.7)
Rwanda	127 299 (86 756 to 183 785)	1 173 (800 to 1 666)	-49.8 (-61.9 to -29.4)	12 260 (8 881 to 16 285)	142 (103 to 187)	-35.9 (-37.7 to -34.2)	139 559 (98 316 to 196 667)	1 315 (937 to 1 800)	-48.6 (-59.3 to -29.9)
Somalia	267 201 (145 842 to 387 984)	1 888 (1 013 to 2 714)	-4.0 (-42.0 to 132.8)	17 191 (12 519 to 22 700)	160 (117 to 211)	-10.2 (-12.0 to -8.5)	284 392 (162 626 to 404 994)	2 048 (1 199 to 2 865)	-4.5 (-39.9 to 106.7)
South Sudan	94 859 (68 486 to 129 489)	1 074 (780 to 1 476)	-10.8 (-41.4 to 80.6)	9 575 (7 003 to 12 638)	153 (112 to 201)	0.0 (-2.2 to 2.0)	104 435 (78 306 to 139 248)	1 227 (934 to 1 635)	-9.6 (-38.1 to 64.6)
Tanzania	282 868 (236 699 to 344 225)	578 (496 to 668)	-33.8 (-49.9 to 18.5)	37 505 (27 154 to 49 894)	104 (76 to 138)	-15.5 (-17.5 to -13.6)	320 373 (273 598 to 383 828)	682 (592 to 778)	-31.5 (-46.5 to 11.4)
Uganda	302 286 (218 879 to 395 184)	932 (672 to 1 201)	-0.9 (-29.8 to 35.8)	29 551 (21 376 to 39 296)	129 (94 to 170)	6.0 (3.7 to 8.4)	331 837 (250 138 to 427 542)	1 060 (800 to 1 336)	-0.1 (-25.8 to 31.7)
Zambia	109 195 (91 767 to 130 069)	731 (628 to 845)	-48.2 (-61.0 to -16.2)	12 933 (9 295 to 17 119)	116 (84 to 153)	-25.3 (-26.9 to -23.5)	122 128 (104 256 to 144 241)	847 (738 to 968)	-45.9 (-58.0 to -17.5)
**Southern sub-Saharan Africa**	**1 003 130 (929 641 to 1 085 522)**	**1 270 (1 183 to 1 367)**	**-43.8 (-51.0 to -38.0)**	**92 886 (66 831 to 124 411)**	**135 (97 to 180)**	**-33.9 (-35.7 to -32.0)**	**1 096 017 (1 018 645 to 1 183 250)**	**1 405 (1 313 to 1 515)**	**-43.0 (-49.5 to -37.6)**
Botswana	15 369 (13 095 to 17 646)	677 (578 to 775)	-36.3 (-49.6 to -18.0)	2 264 (1 624 to 3 034)	114 (82 to 153)	-2.0 (-5.4 to 1.1)	17 633 (15 293 to 20 078)	791 (688 to 894)	-32.9 (-45.8 to -16.2)
Lesotho	42 944 (33 748 to 53 052)	2 179 (1 722 to 2 662)	23.7 (-18.6 to 67.4)	2 440 (1 777 to 3 238)	147 (108 to 195)	16.4 (12.7 to 19.7)	45 384 (36 129 to 55 617)	2 327 (1 861 to 2 817)	23.2 (-16.8 to 63.5)
Namibia	23 628 (18 332 to 30 536)	1 012 (792 to 1 296)	-37.0 (-51.2 to -18.9)	2 657 (1 913 to 3 541)	138 (99 to 183)	-22.2 (-24.9 to -19.5)	26 286 (20 768 to 33 207)	1 149 (915 to 1 439)	-35.5 (-48.6 to -19.3)
South Africa	761 989 (700 240 to 840 039)	1 316 (1 217 to 1 443)	-51.6 (-56.5 to -46.8)	72 377 (52 264 to 96 965)	139 (100 to 186)	-41.4 (-43.3 to -39.3)	834 366 (767 782 to 914 093)	1 455 (1 349 to 1 586)	-50.8 (-55.3 to -46.3)
Swaziland	20 425 (15 596 to 25 353)	1 823 (1 398 to 2 264)	4.2 (-29.6 to 40.9)	1 335 (963 to 1 766)	152 (111 to 202)	-7.2 (-10.0 to -4.2)	21 760 (17 056 to 26 791)	1 976 (1 556 to 2 415)	3.2 (-28.2 to 36.2)
Zimbabwe	138 775 (98 655 to 187 302)	1 059 (747 to 1 446)	12.0 (-30.3 to 48.3)	11 813 (8 510 to 15 533)	112 (81 to 147)	22.6 (19.3 to 25.8)	150 588 (111 350 to 199 453)	1 171 (862 to 1 552)	12.9 (-26.4 to 46.4)
**Western sub-Saharan Africa**	**3 055 422 (2 655 422 to 3 459 838)**	**735 (653 to 827)**	**-31.3 (-40.4 to -19.9)**	**263 396 (191 519 to 348 909)**	**90 (66 to 119)**	**-16.8 (-18.7 to -14.7)**	**3 318 818 (2 920 903 to 3 732 484)**	**825 (740 to 924)**	**-30.0 (-38.6 to -19.6)**
Benin	161 291 (98 062 to 226 277)	1 558 (939 to 2 171)	-38.3 (-50.7 to -23.0)	10 555 (7 633 to 13 938)	138 (100 to 181)	-18.4 (-21.0 to -16.0)	171 846 (107 762 to 237 755)	1 696 (1 076 to 2 318)	-37.0 (-48.7 to -22.7)
Burkina Faso	190 544 (151 688 to 241 192)	952 (786 to 1 135)	-17.2 (-34.3 to 4.5)	8 697 (6 286 to 11 561)	58 (42 to 77)	30.4 (26.2 to 34.2)	199 241 (160 444 to 249 863)	1 010 (847 to 1 197)	-15.4 (-31.9 to 5.5)
Cameroon	209 089 (161 285 to 266 986)	861 (672 to 1 089)	-38.1 (-53.2 to -20.6)	17 585 (12 676 to 23 301)	93 (68 to 123)	-29.8 (-31.7 to -28.0)	226 673 (178 727 to 286 880)	954 (760 to 1 188)	-37.4 (-51.0 to -21.5)
Cape Verde	1 865 (1 569 to 2 151)	343 (292 to 394)	-1.6 (-18.1 to 17.7)	392 (280 to 525)	78 (56 to 104)	13.1 (8.8 to 17.7)	2 257 (1 957 to 2 581)	421 (367 to 479)	0.9 (-13.4 to 17.0)
Chad	151 052 (120 467 to 191 963)	1 021 (824 to 1 296)	3.6 (-17.4 to 29.8)	8 823 (6 408 to 11 567)	96 (70 to 125)	3.0 (0.7 to 5.4)	159 875 (129 324 to 201 368)	1 117 (919 to 1 406)	3.5 (-16.3 to 26.5)
Cote d'Ivoire	190 270 (158 093 to 231 521)	842 (709 to 1 004)	-22.9 (-38.6 to -4.9)	17 827 (12 884 to 23 458)	100 (72 to 131)	-14.9 (-16.8 to -12.9)	208 098 (175 439 to 248 868)	941 (804 to 1 100)	-22.2 (-36.3 to -5.9)
The Gambia	13 712 (9 937 to 18 129)	755 (574 to 954)	-15.5 (-36.2 to 9.6)	1 346 (982 to 1 774)	92 (67 to 121)	-15.1 (-17.3 to -13.0)	15 058 (11 197 to 19 549)	846 (664 to 1 058)	-15.5 (-34.5 to 6.4)
Ghana	261 092 (217 551 to 314 793)	938 (794 to 1 113)	-9.6 (-30.6 to 13.7)	25 097 (18 173 to 33 320)	109 (80 to 145)	7.7 (4.4 to 11.0)	286 188 (241 971 to 340 475)	1 048 (897 to 1 227)	-8.1 (-27.5 to 12.9)
Guinea	101 824 (83 690 to 123 674)	935 (784 to 1 106)	-33.9 (-46.8 to -15.1)	7 906 (5 777 to 10 441)	99 (72 to 130)	-20.3 (-22.9 to -17.8)	109 730 (91 471 to 131 593)	1 034 (882 to 1 210)	-32.8 (-44.9 to -15.5)
Guinea-Bissau	20 408 (15 360 to 26 445)	1 250 (987 to 1 563)	-45.0 (-57.3 to -28.3)	1 187 (865 to 1 561)	94 (69 to 124)	-28.9 (-30.8 to -27.1)	21 595 (16 576 to 27 527)	1 345 (1 081 to 1 655)	-44.1 (-55.7 to -28.4)
Liberia	26 188 (20 445 to 39 119)	610 (496 to 784)	-49.7 (-60.9 to -31.4)	2 325 (1 686 to 3 067)	70 (51 to 92)	-31.6 (-33.7 to -29.6)	28 513 (22 737 to 41 876)	680 (566 to 856)	-48.3 (-58.9 to -31.4)
Mali	188 987 (146 251 to 244 582)	858 (690 to 1 106)	-50.3 (-60.7 to -33.7)	11 414 (8 293 to 15 041)	88 (64 to 115)	-30.6 (-33.1 to -28.2)	200 402 (157 901 to 255 603)	946 (776 to 1 207)	-49.0 (-58.6 to -33.6)
Mauritania	34 035 (28 829 to 39 469)	929 (784 to 1 085)	-48.4 (-57.2 to -35.5)	2 160 (1 567 to 2 860)	77 (56 to 101)	-32.1 (-34.0 to -30.0)	36 195 (30 991 to 41 625)	1 006 (857 to 1 161)	-47.4 (-55.7 to -35.4)
Niger	149 418 (108 319 to 202 092)	719 (528 to 966)	-45.8 (-58.7 to -26.6)	10 748 (7 763 to 14 143)	86 (63 to 113)	-27.5 (-29.8 to -25.3)	160 166 (118 397 to 213 722)	806 (615 to 1 053)	-44.3 (-56.4 to -26.6)
Nigeria	1 145 211 (757 600 to 1 496 836)	541 (378 to 711)	-34.4 (-50.0 to -11.6)	117 833 (85 621 to 156 640)	86 (62 to 114)	-18.2 (-20.6 to -15.4)	1 263 044 (864 674 to 1 618 968)	627 (461 to 802)	-32.6 (-46.7 to -12.4)
Sao Tome and Principe	1 176 (812 to 1 505)	633 (409 to 801)	-12.1 (-40.2 to 14.5)	146 (106 to 194)	96 (70 to 128)	-9.5 (-12.8 to -6.4)	1 322 (949 to 1 644)	729 (510 to 903)	-11.8 (-36.6 to 11.5)
Senegal	87 552 (68 558 to 139 893)	659 (526 to 992)	-29.1 (-42.7 to -5.3)	9 402 (6 844 to 12 377)	90 (66 to 118)	-21.7 (-23.8 to -19.6)	96 954 (77 126 to 148 265)	748 (609 to 1 087)	-28.3 (-40.6 to -7.3)
Sierra Leone	66 081 (54 172 to 80 829)	895 (747 to 1 083)	-32.9 (-47.1 to -11.3)	4 871 (3 520 to 6 419)	90 (66 to 118)	-23.7 (-26.0 to -21.6)	70 951 (58 303 to 86 148)	984 (828 to 1 183)	-32.2 (-45.3 to -12.5)
Togo	55 597 (44 150 to 70 287)	839 (674 to 1 044)	-23.2 (-44.0 to 3.3)	5 080 (3 676 to 6 699)	93 (67 to 122)	-21.3 (-23.9 to -18.7)	60 678 (49 056 to 75 877)	932 (770 to 1 138)	-23.0 (-42.2 to 0.9)
